# Independent specialisation of myosin II paralogues in muscle *vs*. non-muscle functions during early animal evolution: a ctenophore perspective

**DOI:** 10.1186/1471-2148-12-107

**Published:** 2012-07-02

**Authors:** Cyrielle Dayraud, Alexandre Alié, Muriel Jager, Patrick Chang, Hervé Le Guyader, Michaël Manuel, Eric Quéinnec

**Affiliations:** 1 Université Pierre et Marie Curie - Paris 6, UMR 7138 CNRS MNHN IRD, Case 05, 4ème étage, Bâtiment A, 7 quai St Bernard, Paris 75005, France; 2 Department of Biophysics, Laboratory of Molecular Developmental Biology, Graduate School of Science, Kyoto-University, Kitashirakawa-Oiwake, Sakyo-ku, Kyoto, 606-8502, Japan; 3 Université Pierre et Marie Curie- Paris 6, UMR 7009 CNRS, Observatoire Océanologique, Villefranche-sur-Mer, 06230, France; 4 UPMC Université Paris 6, UMR 7138 Systématique, Adaptation, Evolution CNRS MNHN IRD, Bâtiment A, 4ème étage, Case 05, Université Pierre et Marie Curie, 7 quai St Bernard, Paris 75005, France

## Abstract

**Background:**

Myosin II (or Myosin Heavy Chain II, MHCII) is a family of molecular motors involved in the contractile activity of animal muscle cells but also in various other cellular processes in non-muscle cells. Previous phylogenetic analyses of bilaterian MHCII genes identified two main clades associated respectively with smooth/non-muscle cells (MHCIIa) and striated muscle cells (MHCIIb). Muscle cells are generally thought to have originated only once in ancient animal history, and decisive insights about their early evolution are expected to come from expression studies of Myosin II genes in the two non-bilaterian phyla that possess muscles, the Cnidaria and Ctenophora.

**Results:**

We have uncovered three MHCII paralogues in the ctenophore species *Pleurobrachia pileus*. Phylogenetic analyses indicate that the MHCIIa / MHCIIb duplication is more ancient than the divergence between extant metazoan lineages. The ctenophore MHCIIa gene (*PpiMHCIIa*) has an expression pattern akin to that of "stem cell markers" (*Piwi, Vasa*…) and is expressed in proliferating cells. We identified two MHCIIb genes that originated from a ctenophore-specific duplication. *PpiMHCIIb1* represents the exclusively muscular form of myosin II in ctenophore, while *PpiMHCIIb2* is expressed in non-muscle cells of various types. In parallel, our phalloidin staining and TEM observations highlight the structural complexity of ctenophore musculature and emphasize the experimental interest of the ctenophore tentacle root, in which myogenesis is spatially ordered and strikingly similar to striated muscle formation in vertebrates.

**Conclusion:**

*MHCIIa* expression in putative stem cells/proliferating cells probably represents an ancestral trait, while specific involvement of some *MHCIIa* genes in smooth muscle fibres is a uniquely derived feature of the vertebrates. That one ctenophore MHCIIb paralogue (*PpiMHCIIb2*) has retained MHCIIa-like expression features furthermore suggests that muscular expression of the other paralogue, *PpiMHCIIb1*, was the result of neofunctionalisation within the ctenophore lineage, making independent origin of ctenophore muscle cells a likely option.

## Background

Myosins are a tremendously diverse family of actin-binding ATP-dependent molecular motors that appeared and diversified early during eukaryotic evolution [1-5]. All myosins share a homologous myosin head domain containing the ATPase and actin-binding activities, while particular combinations of additional domains define the various myosin “classes” or “types”. In their recent comprehensive analyses of myosin diversity, Odronitz and Kollmar [4] identified 35 myosin classes at the eukaryote scale, of which 3 were present in the last common eukaryotic ancestor. The most intensively studied myosins, class-II myosins (also called “conventional myosins”, myosin II, or MHCII) are characterised by the insertion of a glycin (at position 507) in the head domain, the presence of a SH3 domain N-terminal to the head domain, and a long C-terminal tail mostly comprised of a coiled-coil domain. Class II myosins originated in unikonts, *i.e.* eukaryotes ancestrally bearing a single flagellum or no flagellum, including the amoebozoans, fungi and holozoans (e.g. choanoflagellates and multicellular animals or Metazoa) [5].

Myosin II is a phylogenetically well-defined and diversified class [6], to which notably belong the well-known myosins that provide the physical force for muscle contraction in animals. Beyond these crucial functions in animal muscle cells, a wide range of non-muscle myosin II functions is documented. Myosin II is for example involved in amoeboid motility in the unicellular life stage of the amoebozoan *Dictyostelium discoideum*[7]. In metazoans, various myosin II proteins play pivotal roles in cytokinesis [8,9], cell migration [10-12], cell-cell adhesion [12,13], or cell polarity [11,14,15]. Therefore, myosins II regulate fundamental aspects of cellular shape morphology [16-18], cytokinesis [19], cell differentiation [20,21] and more generally, many aspects of cellular behaviour [22]. Whatever the context (muscle or non-muscle), MHCII are integrated within macromolecular complexes notably through direct interaction with smaller proteins called “myosin light chains” (MLC) (which lack a head domain).

Previous analyses of myosin II proteins in bilaterian animals have recognised two phylogenetic groups, the first containing genes expressed in smooth muscle cells and in non-muscle cells, while genes of the second group are specifically expressed in striated (skeletal or cardiac) muscle cells [2,23]. This major dichotomy has been thought to reflect independent evolutionary origin from non-muscle cells for each major type of muscle cells, *i.e.* smooth muscle cells and striated muscle cells [1,24], through independent co-option of different myosin II paralogues. However, this suggestion was based on data from bilaterian animals only and remained highly speculative.

Outside from the bilaterian clade, muscle cells are present in two animal phyla, Cnidaria and Ctenophora, and this cell type is classically viewed as a synapomorphy (shared derived character) of the Eumetazoa (Cnidaria + Ctenophora + Bilateria), together with nerve cells. Most cnidarian muscle cells are in fact multifunctional myoepithelial cells [25], integrated within the ectoderm and endoderm [26], although there are some reported instances of mesogleal muscle cells in cnidarians [27-30]. The myoepithelial cell of cnidarians typically comprises a contractile portion (the muscle fibre or myoid) attached to a globular cellular body involved in other functions (e.g. body protection, glandular secretion, fluid circulation through ciliary beating, etc.) [30]. In contrast, ctenophores are commonly considered to have true muscle cells, *i.e.* their muscle fibres lack a cellular body with filament-free cytoplasm ( [31] but see [32] for an alternative point of view). In addition, part of the ctenophore musculature is located in the mesoglea and develops from a mesodermal-like germ layer [33,34]. Therefore, comparative studies using ctenophores have a great potential for improving our understanding of the early evolution of muscle cells and muscular protein families.

The phylogenetic position of ctenophores is still debated. Some recent phylogenomic analyses have placed them as the sister group to all other metazoans [35,36], but this result was probably due to an artefact of long-branch attraction [37-39]. According to the phylogenomic analyses of Philippe *et al.*[37], ctenophores and cnidarians form a coelenterate clade, sister-group to the bilaterians, within monophyletic Eumetazoa.

**Figure 1:**
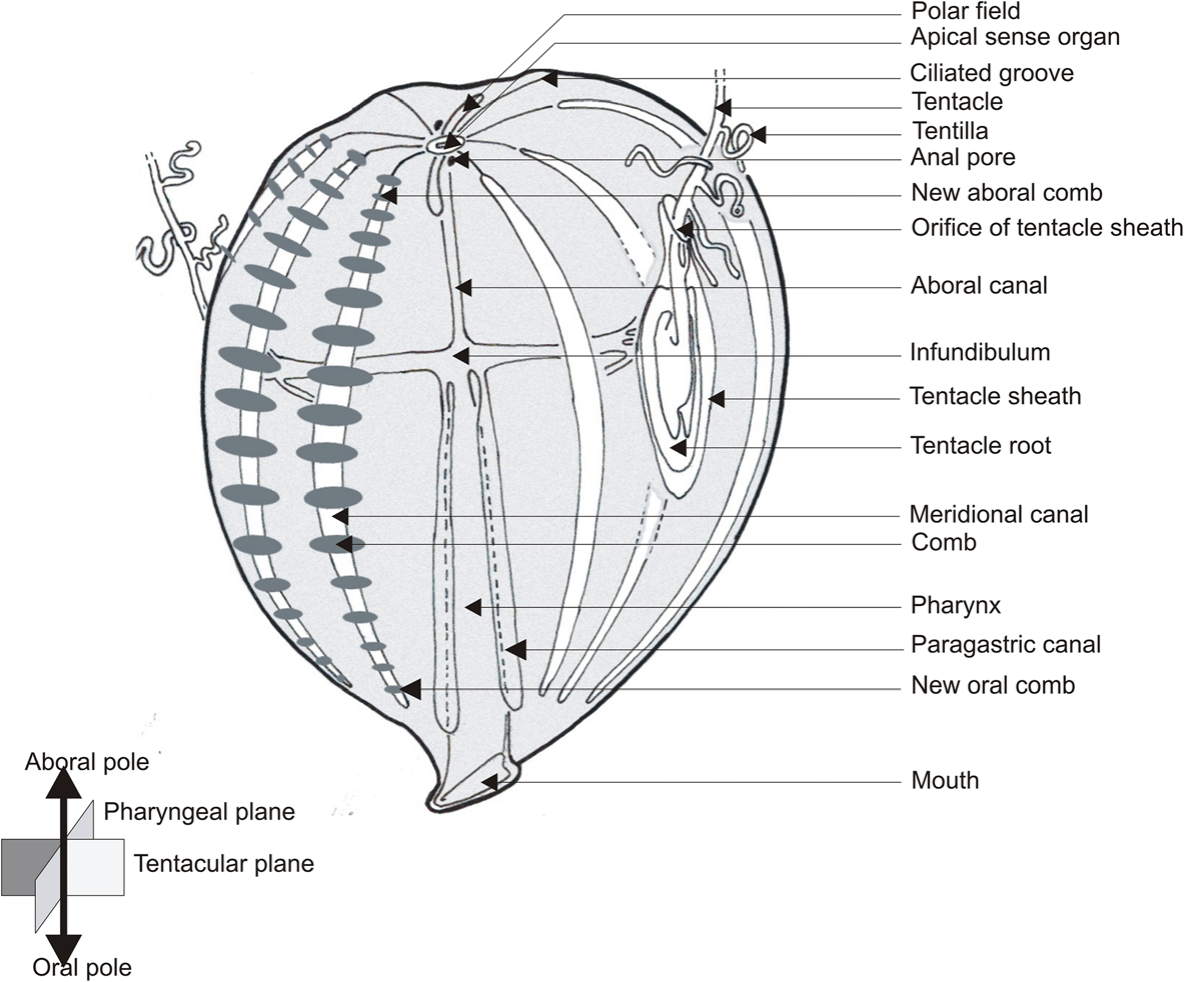
**General organisation of *Pleurobrachia pileus.***

Ctenophores are marine animals with a highly original biradially-symmetrical body plan and featuring unique anatomical traits (Figure 1) [26,31]. Their main distinctive feature is a locomotory system consisting of eight distinctive meridional rows of comb plates (swimming paddles), each made of the many fused giant cilia of “polster cells”. At their aboral pole, ctenophores possess an apical sensory organ involved in equilibration and flanked by two elongated ciliated areas called polar fields. There are two distinct nerve nets extending throughout the body, the epithelial (or polygonal) nerve net and the mesogleal nerve net [40]. The gastro-vascular system, of mainly endodermal origin, opens at one extremity by the mouth and at the other by two anal pores. The ramified gastro-vascular canal system allows water circulation and distribution of nutrients throughout the body. Ctenophores are hermaphrodite, with paired male and female gonads housed in the walls of eight endodermal meridional canals, placed under each of the comb rows. Most ctenophores catch prey by using a pair of long and contractile tentacles which bear lateral branches or tentillae on their oral side. The epidermis of tentacles and tentillae is densely covered with adhesive cells called colloblasts, to which the prey adheres. Tentacles can extend from and retract into a tentacular sheath whose epithelial lining is continuous with the outer epidermis.

**Figure 2:**
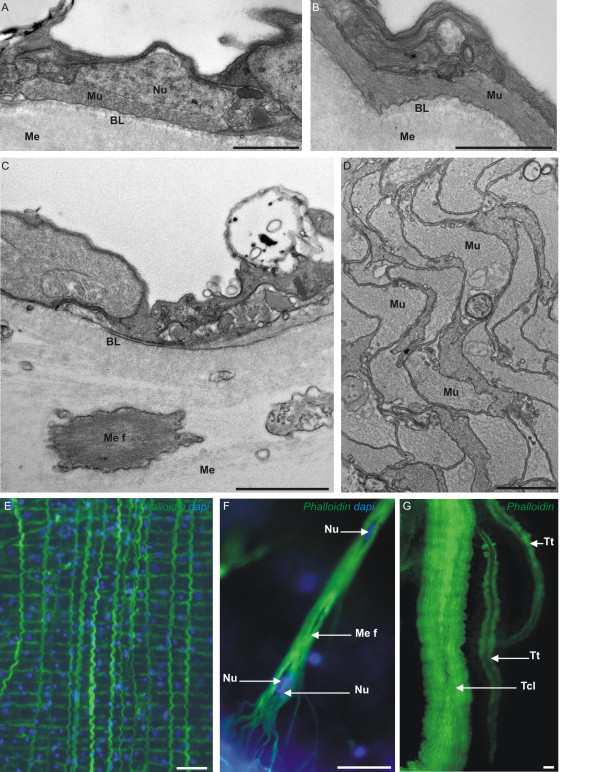
***Pleurobrachia pileus *(ctenophore) muscle cells.** (**A**-**D**) Transmission electron microscopy (TEM) views of * Pleurobrachia pileus * muscle cells. (**A**-**B**) Sections through the external epithelium showing a parietal muscle cell (Mu) in transverse section (**A**) and in longitudinal section (**B**). (**C**) Section showing a portion of mesoglea with a mesogleal muscle fibre (Me f) cut obliquely. (**D**) Tentacle muscle cells (Mu) in transverse section. (E-G) Pictures showing the aspect of the main muscle cell types on phalloidin-stained preparations. (**E**) Parietal muscle fibres. (**F**) Close-up of a mesogleal muscle fibre showing its ramified extremity and its multinucleate organisation. (**G**) Multiple parallel muscle fibres in a tentacle (Tcl) and two tentillae (Tt). BL: Basal Lamina; Me: Mesoglea; Me f: Mesogleal muscle fibre; Mu: Muscle fibre; Nu: Nucleus; Tcl: Tentacle; Tt: Tentilla. Scale bars: A, B: 1 μm; C: 2 μm; D: 5 μm; E, F, G: 20 μm.

In the ctenophore order Cydippida, to which belong our model species *Pleurobrachia pileus*, two fundamental categories of muscle cells can be distinguished: parietal muscle fibres are located in the basal part of the ectodermal epithelium, above the basal lamina (Figure 2A, B, E), while mesogleal muscle fibres are positioned below the basal lamina and run through the mesoglea (Figure 2C, F) [31]. In addition, a special mesogleal musculature is housed in the core of tentacles and tentillae (Figure 2D, G). The very particular “mesogleal giant smooth fibres” of ctenophores belonging to the order Beroida (which lack the parietal musculature [31]) have been the subject of specific studies [41-44], while other muscle cell types, and muscles of other ctenophores, are much less known. Ctenophore muscles are considered to be of the smooth type due to the absence of a striation pattern on electronographies (except in the tentillae of the cydippid ctenophore *Euplokamis*[31,45]), but their mesogleal muscle cells are multinucleated [31], which represents a fundamental difference with the mononucleated smooth muscle cells of bilaterians.

In tentaculate ctenophores, myogenesis is particularly intense throughout the life span in the thickened tentacle base (tentacle root), where histogenesis continuously compensates for the loss of tentillae and tentacle pieces that are damaged upon feeding. Experimental data indicate that 36 hours are sufficient for regeneration of an entire tentacle [46]. Putative stem cells of the colloblasts and muscle cells have been recently characterised in the tentacle root of the ctenophore *Pleurobrachia pileus* by expression analyses of *Piwi* and *Vasa* genes and DNA-labelling experiments (Figure 3 in [47]). The muscle putative stem cells and progenitors are localised along a median ridge in the symmetry plane of the tentacle root on its internal face [47-51]. Thanks to these characteristics, the tentacle root is a particularly suitable model to investigate myogenesis in ctenophores.

**Figure 3:**
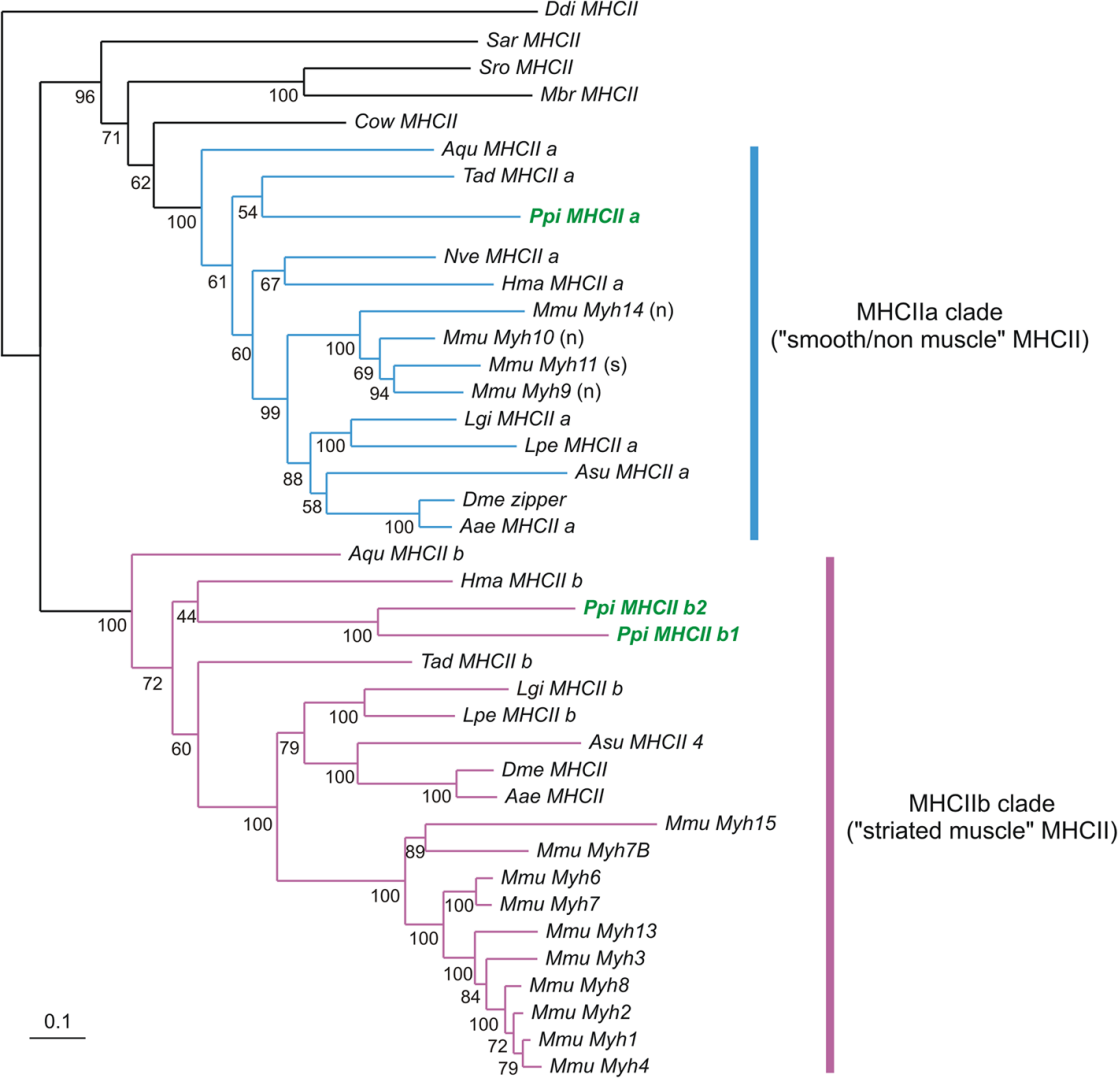
**Phylogenetic analysis of *Pleurobrachia pileus *MHCII sequences.** Amino-acid sequences were analysed using the Maximum likelihood (ML) method. Numbers associated with the branches are ML bootstrap values (1000 replicates). Sequences from * Pleurobrachia pileus * are indicated in bold font and green colour. The MHCIIa clade is indicated in blue and the MHCIIb clade in purple. The letter between parentheses after the name of mouse genes of the MHCa clade indicate whether the gene is expressed in non-muscle cells (n) or in smooth muscle cells (s). Abbreviations for species names: Aae: * Aedes aegypti *; Aqu: *Amphimedon queenslandica*; Asu: *Ascaris suum*; Cow: *Capsaspora owczarzaki*; Ddi: * Dictyostelium discoideum *; Dme: *Drosophila melanogaster*; Hma: *Hydra magnipapillata*; Lgi: *Lottia gigantea*; Lpe: *Loligo pealei*; Mbr: *Monosiga brevicollis*; Mmu: *Mus musculus*; Nve: *Nematostella vectensis*; Ppi: *Pleurobrachia pileus*; Sar: *Sphaeroforma arctica*; Sro: *Salpingoeca rosetta*; Tad: *Trichoplax adhaerens.*

We studied the expression of three paralogous class II myosin (*MHCII*) genes in the adult of the cydippid ctenophore *Pleurobrachia pileus*. In parallel, the complex organisation of muscle fibres throughout the *Pleurobrachia pileus* body was investigated using phalloidin staining to help understand the gene expression patterns. One of the *MHCII* paralogues was specifically expressed in muscle cells (parietal and mesogleal) while the other two had non-muscle expression. Several evolutionary scenarios are proposed to interpret the ctenophore *MHCII* expression data in light of our reconstruction of the *MHCII* gene phylogeny and of the known functions of bilaterian *MHCII* genes.

## Methods

### Specimen collection

Adult specimens of *Pleurobrachia pileus* were collected in Villefranche-sur-Mer and in Roscoff (France) between March and June using specific plankton nets. They were kept at 16°C in filtered natural seawater, under continuous water circulation and fed daily with *Artemia* nauplii.

### Blast searches and phylogenetic analyses

MHCII sequences were retrieved by TBLASTN searches using *Mus musculus MHCII* sequences on two *Pleurobrachia pileus* transcriptome assemblies: (i) Phrap assembly of a collection of about 36 000 ESTs, sequenced from animals collected in Villefranche-sur-Mer (France) by the Sanger method at the Genoscope (Evry, France) (see [47] for details), and publicly available in dbEST ( http://www.ncbi.nlm.nih.gov/nucest?term=pleurobrachia) and (ii) Newbler assembly of one run of 454 sequencing of total RNA extracted from mixed embryonic, larval, and adult stages with starting material obtained from Roscoff (France).

The alignment was constructed using published sequences and sequences retrieved by TblastN searches on public databases for a representative taxon set. Sequences were aligned using MUSCLE [52]. Ambiguous regions of the alignment were removed using Gblocks [53]. The initial alignment (before removal of ambiguously-aligned regions) is provided in Additional file 1. This alignment contains the head domain of several myosins belonging to classes other than class II (outgroups). After pruning of ambiguously-aligned sites, two final sub-alignments were extracted to take into account the absence of sequence data for the head domain in one of the ctenophore sequences (*PpiMHCIIb2*): (i) a first alignment containing the outgroup sequences (non-class II myosins). The *PpiMHCIIb2* gene was excluded from this alignment because its partial sequence contains only the tail and therefore it has no alignable residues with non-class II myosins (alignment in Additional file 2; resulting tree shown in Additional file 3); (ii) a second alignment, in which non-class II myosin sequences have been eliminated, thus allowing inclusion of *PpiMHCIIb2* (Alignment in Additional file 4; resulting tree, rooted on ameobozoan MHCII, shown in Figure 3). Maximum-likelihood analyses were performed using the PhyML program [54], with the WAG model of amino-acid substitution and a BioNJ tree as the input tree. A gamma distribution with eight categories was used. The gamma shape parameter and the proportion of invariant sites were optimised during the searches. The statistical significance of the nodes was assessed by bootstrapping (1000 replicates).

### *In situ* hybridisation

Adults were fixed at 4°C in 4% paraformaldehyde in 50% seawater and 50% PBST (10 nM Na_2_HPO_4_, 150 nM NaCl, pH 7.5, 0.1% Tween 20), for 1 hour then washed three times in PBST and dehydrated through a graded series of ethanol and stored in methanol at −20°C. The *in situ* hybridisation (ISH) protocol was as described in [55] but colour was developed with NBT/BCIP (Roche Diagnostics, Meylan, France). After ISH, samples were stained with DAPI (1 μg/mL) for 15 min to visualise DNA, and then washed 3 times for 15 min in PBST. Before mounting in Citifluor solution (Oxford instruments SAS, Saclay, France), animals were dissected. Longitudinal and transverse sections were performed to clarify the precise distribution of the gene expression patterns. For longitudinal views of the median expansion of the tentacle root, the lateral expansions were removed with forceps. Negative controls (with a sense probe and without any RNA probe) performed in parallel showed no staining after extensive revelation, except occasionally in a few isolated cells in the general epithelium (between adjacent comb rows, around the apical organ). For this reason, staining of isolated epithelial cells occasionally obtained with MHCII probes was not taken into account.

### Immunofluorescence

Animals were fixed in 4% paraformaldehyde in 50% seawater and 50% PBST for 30 min, at room temperature, then samples were washed several times in PBST, dehydrated through a graded series of ethanol and stored in methanol at −20°C. After re-hydration to PBS, samples were permeabilised with Triton-X100 (0,2% in PBS, then 0,01% in PBS, 10 min at room temperature). After blocking with 1% bovine serum albumin, samples were incubated with the rat monoclonal anti-tyrosylated α-tubulin or YL1/2 antibody (1:1000 dilution in PBS-Triton-X100 0.01%, BSA 1%), (Morphosys AbD Gmbh, Düsseldorf, Germany) for 4 hours at room temperature. After washing with PBS triton-X100 0.01%, samples were incubated overnight at 4°C with the Alexa Fluor 568 goat anti- rat IgG secondary antibody. Dilutions of primary and secondary antibodies were made using 1X PBS containing 0.01% Triton-X100.

### Phalloidin staining

For phalloidin staining, specimens were not dehydrated after fixation. They were incubated for 45 min in a dilute Phalloidin-TRITC (Sigma-Aldrich, St-Quentin-Fallavier, France) solution (10 μg/ml in PBST) and rinsed three times in PBST. All specimens were finally stained with DAPI (1 μg/ml) for 15 min for DNA visualisation, and then washed three times for 15 min in PBST. They were micro-dissected and mounted in Citifluor solution (Oxford instruments SAS, Saclay, France).

### EdU labelling of DNA-replicating cells

EdU (ethynyl deoxyuridine) is a thymidine analogue similar to the classical BrdU but quicker and easier to use [56]. EdU incorporation assays were done using the Click-it EdU Alexa Fluor 488 Imaging Kit from Invitrogen (Cergy-Pontoise, France). The protocol was as described in [47]. We performed 12 hours of pulse and no chase to visualise proliferating cells in the aboral region.

### Sections for light and transmission electron microscopy (TEM)

Two types of sections (cryosection and ultra-thin section) were performed. For cryosectioning, tentacle roots extracted after whole mount ISH were incubated for two days in PBST 1X, 15% sucrose at 4°C, then for 2 hours in PBST 1X, 15% sucrose, 7.5% pig gelatine. Then, blocks were frozen at −65°C in 2-methyl-butan. Cryosectioning was done on a Leica CM3050 S cryostat or on a Jung Frigocut 2800E cryostat, at a thickness of 14 μm. Slices were mounted in Citifluor (Oxford instruments SAS, Saclay, France).

*Pleurobrachia pileus* living specimens for ultra thin sections were fixed for 10 minutes at room temperature in 3% glutaraldehyde, 0.1 M sodium cacodylate pH 7.3, 0.3 M sodium chloride, 0.05% OsO_4_ (modified after [57]). They were then transferred to the same solution without OsO_4_ for 2 hours. Specimens were then post-fixed for 1 hour in 1% OsO_4_, 1.5K-ferricyanide, 2.5% NaHCO_3_ pH 7.2 and 0.25 M sodium chloride (modified after [58]). Finally, material was dehydrated through an ethanol series, and embedded in Spurr. Sections were done using a Leica Ultracut R ultra-microtome, at a thickness of 60 nm for ultra thin sections.

### Imaging

Pictures of *Pleurobrachia pileus* entire specimen and tentacle root observed *in toto* were acquired on a stereo-microscope Olympus SZX12 using a Q-imaging QICAM with Image pro software (Mediacybernetics, Bethesda, MD). All fluorescence and DIC images were acquired on an Olympus BX61 microscope using a Q-imaging Camera with Image Pro plus software (Mediacybernetics, Bethesda, MD). To enhance some details, Higauss filter was used on some pictures (from Image Pro). TEM images were acquired on a Jeol JEM-1400 equipped with a Morada (SIS) camera at the “Centre Commun de Microscopie Appliquée” (CCMA) (Université de Nice Sophia-Antipolis, Faculté des Sciences, Nice).

## Results

### Ancestral duplication of class II Myosin Heavy Chain genes in metazoans

By Blast searches on *Pleurobrachia pileus* ESTs and phylogenetic analyses we could identify three ctenophore Myosin Heavy Chain II genes (MHCII). Although these searches were conducted on relatively extensive transcriptomic data, and myosin genes are expected to be expressed at a high level, we cannot exclude the existence of additional paralogues in the *Pleurobrachia pileus* genome. Of these three identified *Pleurobrachia pileus* paralogues, one (*PpiMHCIIa*) falls in a clade containing all bilaterian *MHCII* genes expressed in smooth muscle cells and/or non-muscle cells (MHCIIa clade: blue colour in Figure 3), while the two remaining genes (*PpiMHCIIb1* and *PpiMHCIIb2*) branch with bilaterian *MHCII* genes expressed in striated muscle cells (MHCIIb clade, highlighted in purple colour in Figure 3). We conducted two phylogenetic analyses with different outgroup gene samplings, providing essentially similar results: an analysis rooted using the MHCII sequence of the amoebozoan *Dictyostelium discoideum* (Figure 3) and another analysis, rooted with sequences of myosin classes V, VII and X (Additional file 3) (see Methods for explanations).

The presence of genes from ctenophore, cnidarians (*Hydra magnipapillata* and *Nematostella vectensis*) but also sponge (*Amphimedon queenslandica*) and placozoan (*Trichoplax adhaerens*) in both MHCII clades indicates that the duplication happened before the last common ancestor of metazoans. Other holozoans (*Monosiga brevicollis, Salpingoeca rosetta, Capsaspora owczarzaki* and *Sphaeroforma arctica*) each have one orthologue of *MHCIIa* but no *MHCIIb* gene, suggesting that the MHCIIa/MHCIIb duplication occurred in the last common ancestor of holozoans and was followed by independent *MHCIIb* losses in the various unicellular holozoan lineages. An alternative explanation could be that the duplication took place in a metazoan ancestor, but that sequences of *MHCIIb* are misplaced in the tree due to a higher subsequent sequence divergence. In vertebrates, the subdivision of *MHCII* genes into MHCIIa and MHCIIb orthology groups is clearly correlated with functional specialisation, *MHCIIb* genes being associated with striated muscle cells and *MHCIIa* genes being expressed in either non-muscle or smooth muscle cells. In addition, independent diversification within both groups occurred in vertebrates (Figure 3, Additional file 3). Finally, the two ctenophore *MHCIIb* paralogues *PpiMHCIIb1* and *PpiMHCIIb2* are quite divergent but they clearly originated from a ctenophore-specific duplication within the MHCIIb group (Figure 3).

### The three MHCII paralogous genes are differentially expressed in the *Pleurobrachia pileus* tentacle root

**Figure 4:**
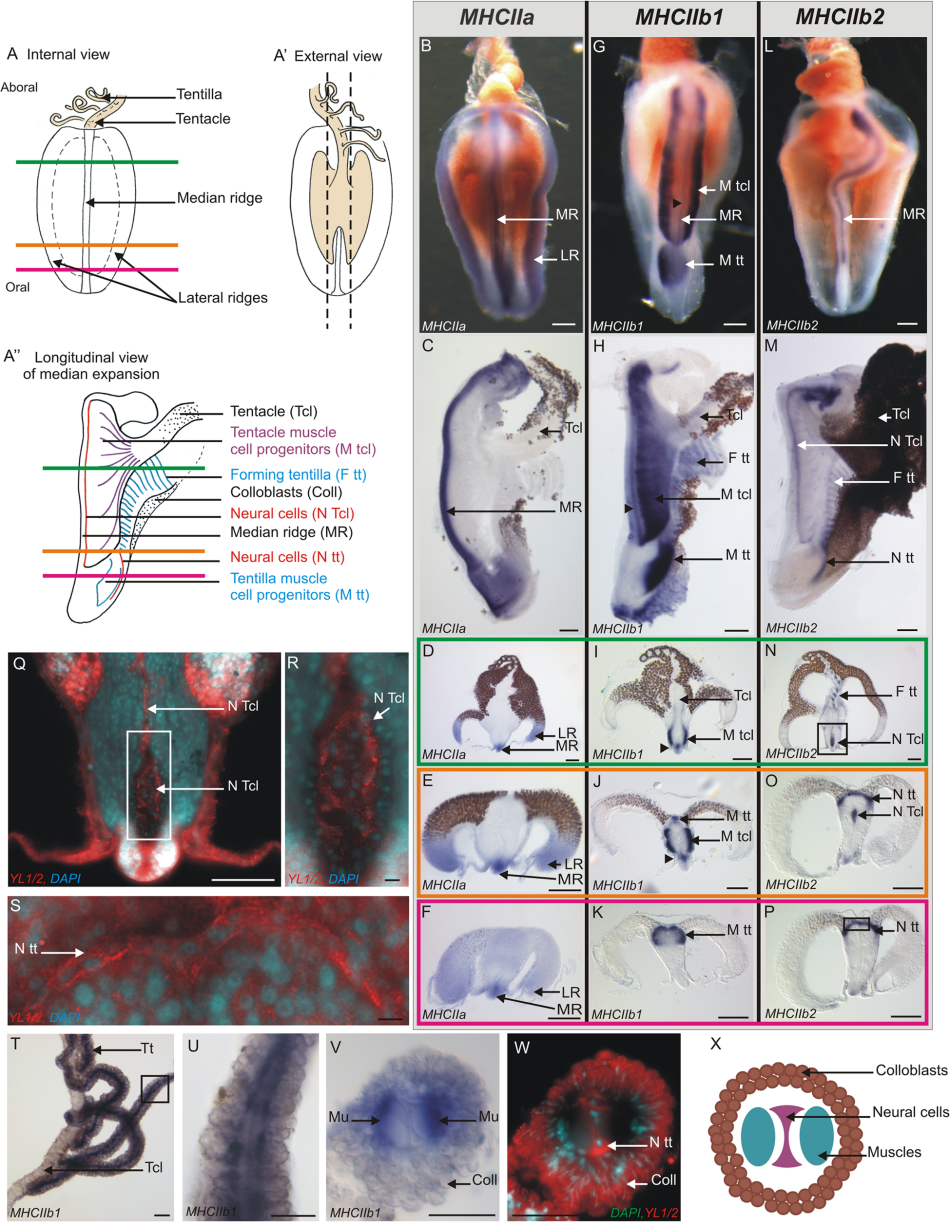
**Expression of *PpiMHCIIa, PpiMHCIIb1 *and *PpiMHCIIb2 *in the tentacular apparatus.** (**A**) Schematic drawing of the tentacle root in internal view. The three coloured horizontal lines materialise the three different planes of transverse cryosections corresponding to panels **D**, **I**, **N** (in green), **E**, **J**, **O** (in orange) and **F**, **K**, **P** (in purple). (**A’**) The tentacle root in external view (corresponding to the side of tentacle insertion). The dotted lines delineate the longitudinal dissections to remove tentacle root lateral expansions in order to obtain the preparations shown in (**C**, **H**, **M**). (**A”**) Schematic drawing of the tentacle root in longitudinal section, after removal of the lateral expansions following the dotted lines in (**A’**). Horizontal lines indicating the three planes of cryosections as in (**A**). (**B**, **G**, **L**) Internal views of isolated tentacle roots showing *PpiMHCIIa* (**B**), *PpiMHCIIb1* (**G**), *PpiMHCIIb2* (**L**) expressions. (**C**, **H**, **M**) Longitudinal sections of the tentacle root stained with *PpiMHCIIa* (**C**), *PpiMHCIIb1* (**H**), *PpiMHCIIb2* (**M**) anti-sense probes. The aboral pole is at the top in panels (**B**, **C**, **G**, **H**, **L**, **M**). (**D**-**F**, **I**-**K**, **N**-**P**) Transverse cryosections of whole-mount ISH for the three genes, with sectioning plane indicated by the colour of the surrounding line according to the colour code outlined in panel (**A**). The black arrowhead in (**G**-**J**) points to a stained line above the median ridge which appears to be more or less in continuity with the two layered bands labelled M Tcl. (**Q**) YL1/2 (anti-tyrosylated-α-tubulin, in red) and DAPI (in blue) counterstaining of the region boxed in (**N**). (**R**) Higher magnification of the region indicated by the box in (**Q**). (**S**) YL1/2 (red) and DAPI (blue) counterstaining of the region boxed in (**P**). (**T**-**W**) Expression of *PpiMHCIIb1* gene in tentillae. (**T**) Whole mount ISH of tentacle and tentillae. (**U**) Higher magnification view of the region boxed in (**T**). (**V**) Transverse cryosection of whole-mount ISH of a tentilla. Two symmetrical muscle fibres are stained. (**W**) YL1/2 (in red) and DAPI (in blue) counterstaining of (**V**). The YL1/2 staining in colloblasts is certainly due to non-specific fixation of the antibody on the sticky colloblast granules. (**X**) Schematic drawing of a tentilla in transverse section. Coll: Colloblasts; F tt: Forming tentillae; LR: Lateral Ridge; MR: Median Ridge; M tcl: Tentacle Muscle progenitors; M tt: Tentilla muscle progenitors; Mu: Muscle fibres; N Tcl: Tentacle Neural cells; N tt: Tentilla Neural cells Tcl: Tentacle; Tt: Tentilla. Scale bars: B, C, G, H, L, M: 100 μm; D-F, I-K, N-P: 200 μm; Q: 100 μm; R, S: 10 μm; T: 50 μm; U, V, W: 25 μm.

In the tentacle root, *PpiMHCIIa* is expressed in the putative stem cells/undifferentiated progenitors of colloblasts and muscle cells. The morphology of the tentacle root (in internal, external and longitudinal views) is summarised in Figure 4A-A”. The expression of *PpiMHCIIa* closely looks like that of the “stem cell genes” investigated in Alié *et al.*[47]. On the internal side of the tentacle root, the *PpiMHCIIa* antisense RNA probe stains three longitudinal lines running from the oral to the aboral pole of the tentacle root (Figure 4B): one median line and two lateral lines. In longitudinal section (Figure 4C), the median staining appears confined to the distal extremity or ridge of the median expansion, although it is more diffuse towards both poles. In transverse sections of the tentacle root after whole-mount *PpiMHCIIa* ISH, transcripts appear clearly confined to the three ridges of the tentacle root expansions (Figure 4D, E), where putative stem cells are localised according to a previous study [47]: muscle stem cells in the median ridge and colloblast stem cells in the lateral ridges. Transcript distribution appears more diffuse when the sectioning plane is closer to the oral pole (Figure 4F), consistent with staining distribution in longitudinal view.

**Figure 5:**
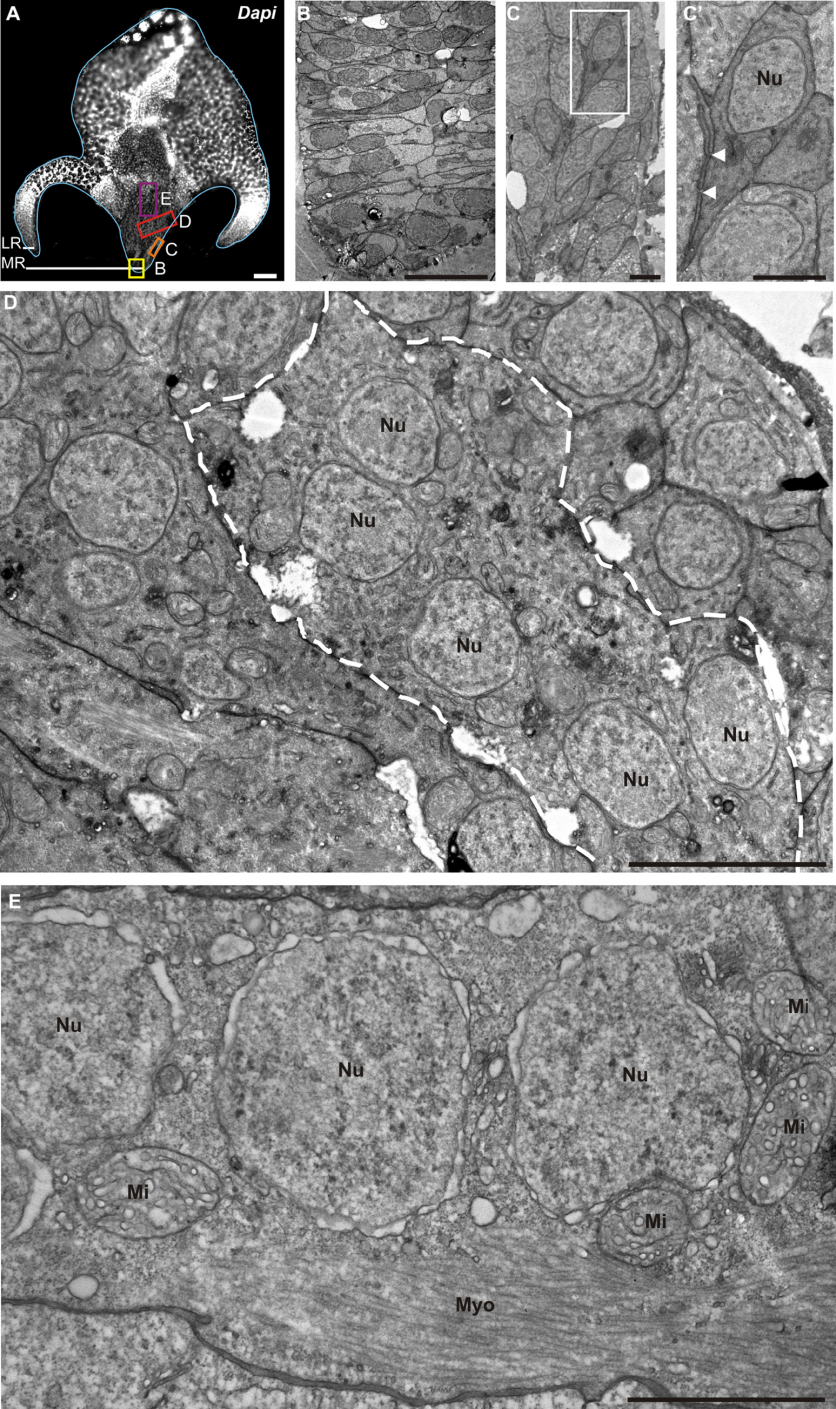
**Stages of tentacle muscle differentiation observed using TEM.** (**A**) DAPI staining of a transverse section of the tentacle root. The light blue line delineates the tentacle root. (**B**-**E**) TEM images of myogenesis stages in the median expansion of the tentacle root. (**B**) TEM view of the region boxed in yellow in (**A**), showing the muscle stem cells / progenitors. (**C**) TEM view of the region boxed in orange in (**A**) showing spindle-shaped muscle cell progenitors. (**C’**) Higher magnification of the region boxed in (**C**). White arrowheads point to cytoplasmic connections and plasma membrane interdigitations. (**D**) TEM view of the region boxed in red in (**A**), showing a muscle cell in differentiation (delineated by the white dotted line), and resulting from the fusion of several muscle cell progenitors, as indicated by the multinucleate organisation of the cell. These cells exhibit a developed endoplasmic reticulum, many mitochondria and are devoid of myofilaments. (**E**) TEM view of the region boxed in purple in (**A**), showing a mature muscle cell, with aligned nuclei (Nu) and distinct myofilaments (Myo). White holes in (**B**-**D**) are fixation artefacts. LR: Lateral Ridge; Mi: Mitochondria; MR: Median Ridge; Myo: Myofilaments; Nu: Nucleus. Scale bars: A: 100 μm; B: 10 μm; C, C’: 2 μm; D: 5 μm; E: 2 μm.

**Figure 6:**
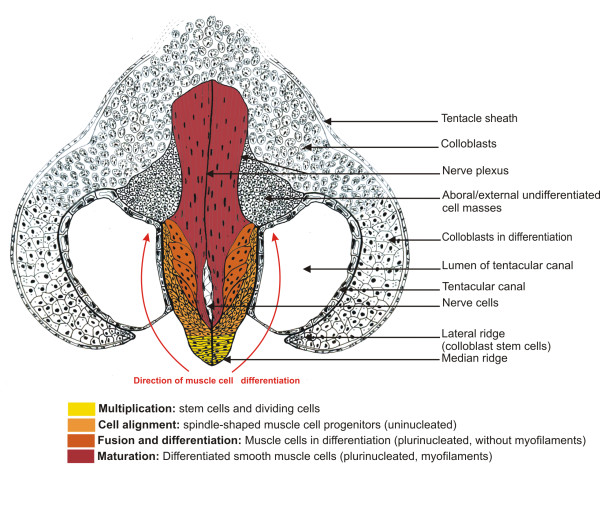
**Dynamic model of myogenesis in the *Pleurobrachia pileus *tentacle root.** Drawing of a tentacle root transverse section at the aboral level illustrating the proximal-distal distribution of myogenesis stages. The four recognised stages along the muscle cell lineage are indicated in bold.

Our TEM observations of the median ridge in transverse section (Figure 5B: picture corresponding to the yellow box in the general DAPI view of Figure 5A) identify muscle putative stem cells as elongated, undifferentiated cells with a high nucleo-cytoplasmic ratio and some mitochondria, as described for muscle cell progenitors by Franc [51]. These cells are organised in two symmetrical and closely tightened adjacent rows (yellow in Figure 6). In the lateral ridges, colloblast putative stem cells are more irregularly arranged (Figure 6); their cytological characteristics have been described elsewhere [47,50]. Differentiating colloblasts, originating from these stem cells, can be traced on transverse sections thanks to their brownish colour which appears approximately where *PpiMHCIIa* expression vanishes (Figure 4D, E).

The *PpiMHCIIb1* gene is expressed in the differentiating muscle cells of the tentacle root. In internal view (Figure 4G), transcripts are detected in two neighbouring but disconnected regions, corresponding respectively to the muscle differentiation zones of the tentacle and of the tentillae: (*i*) two wide bands (M Tcl in Figure 4G) positioned on both sides of the median expansion; (*ii*) an additional domain towards the oral pole (M tt in Figure 4G), where the tentillae are produced. In the longitudinal view section, these two expression domains are also clearly distinct (Figure 4H), with the first here appearing as a wide fan-shaped stained area converging to the base of the tentacle (M tcl in Figure 4A”, H), in addition to a thin stripe located just above the median ridge (black arrowheads in Figure 4G-J). These two *PpiMHCIIb1* expression domains are also shown in transverse section (sectioning planes indicated in Figure 4A): expression in differentiating muscle cells of the tentacle axis is visible on the two aboral-most sections (M tcl in Figure 4I and 4J) and expression in differentiating muscle cells of tentillae is visible on the two oral-most sections (M tt in Figure 4J and 4K).

This interpretation of *PpiMHCIIb1* expression patterns in the tentacle root is supported by our TEM observations, which identify four distinct stages of tentacle muscle differentiation from stem cells to fully differentiated muscle cells (Figure 5B-E; summary drawing in transverse section in Figure 6). The first step corresponds to cell multiplication in the median ridge (Figure 5B and see EdU incorporation assays described in [47]). The 2^nd^ step is represented by spindle-shaped mononucleate progenitors found laterally and aligned in an oblique orientation (Figure 5C-C’; light orange in Figure 6). These cells are connected to each other by cytoplasmic bridges (Figure 5C’, white arrowheads). A more advanced cell stage (step 3) is represented by multinucleated cells (up to 8 nuclei per cell counted), located closer to the symmetry plane of the tentacle root (Figure 5D; dark orange in Figure 6), indicating that progenitors have undergone cell fusion. These cells contain well-developed endoplasmic reticulum and many mitochondria, but are still devoid of myofilaments. Finally, mature muscle cells are observed in two symmetric and median bundles (red in Figure 6), externally surrounded by the two preceding less differentiated cell populations. These differentiated muscle cells are multinucleated and contain dense longitudinal myofilaments as well as many mitochondria (Figure 5E). The first expression domain of *PpiMHCIIb1* described above closely matches the differentiation zone of tentacle muscle (orange colours in Figure 6), an interpretation also supported by the strong expression observed for the muscle differentiation marker gene *Tropomyosin* in exactly the same zone (Additional file 5).

The formation of tentillae is an independent process occurring at the oral pole of the tentacle root. The second domain of *PpiMHCIIb1* expression (M tt in Figure 4G, H, J, K) corresponds to the production zone of the tentillae, and therefore the gene is also expressed in differentiating muscle cells of the tentillae. More aborally, *PpiMHCIIb1* shows a striped pattern in the youngest formed tentillae (F tt in Figure 4H).

In the mature tentacular apparatus, *PpiMHCIIb1* is expressed in the muscle masses housed within the tentillae, but not in those of the tentacle itself (Figure 4T: note the absence of staining in the tentacle, Tcl). In longitudinal and transverse sections of mature tentillae, *PpiMHCIIb1* expression is seen in two parallel bundles corresponding to the position of muscle fibres (Figure 4U-X). These muscle bundles are separated by an unstained central nerve cord, revealed by counter-staining using the structural marker YL1/2 (anti-tyrosylated-α-tubulin antibody) (Figure 4W 4X), known to stain nerve cells with high intensity in *Pleurobrachia pileus*[40]. The expression pattern of *PpiTropomyosin* in the tentilla is identical to that of *PpiMHCIIb1* (compare Additional file 5D with Figure 4V), supporting the muscular nature of *PpiMHCIIb1-*expressing cells*.* The fact that *PpiMHCIIb1* and *PpiTropomyosin* genes (see Tcl in Additional file 5C) are expressed in the mature muscles of tentillae but not of the tentacle (although these genes are expressed in the differentiating muscle of both) indicates that these two kinds of contractile structures have muscle fibres of different types. It is therefore clear that in spite of their topological relationships, tentillae are not just ramifications of the tentacle but instead are original organs, both ontogenetically and structurally.

The expression pattern of the other *MHCIIb* paralogue *PpiMHCIIb2* is sharply different from that of *PpiMHCIIb1* in the tentacle root, as it seems to associate with neural cells instead of muscle cells. In internal and longitudinal views, *PpiMHCIIb2* expression is visible as a thin stripe above the median ridge, which becomes broader at the aboral pole and does not reach the oral extremity (Figure 4L and M). In transverse sections, transcripts were detected in a median group of cells located between the two bundles of differentiated muscle cells (N tcl in Figure 4N and O). Counter-staining of these transverse sections using the anti-tyrosylated-α-tubulin antibody (YL1/2) reveals that the *PpiMHCIIb2*-expressing zone corresponds to a nerve plexus (Figure 4Q). A closer examination of this area reveals the presence of two symmetrical layers of nerve cells sandwiched between tentacle muscles (Figure 4R, Figure 6). This nerve plexus also runs through tentacle and tentillae (data not shown); it is depicted (white area and black line in the median expansion) in Figure 6.

Towards the oral pole, *PpiMHCIIb2* is also expressed in the production zone of the tentillae (N tt and F tt in Figure 4M), in the form of a thin external stripe of positive cells at the interface between the lateral and median expansions (N tt in Figure 4O and P). YL1/2 and DAPI counterstaining reveal the presence of densely packed neural cells with small nuclei on a thin layer corresponding to the zone of *PpiMHCIIb2* expression (Figure 4S, counterstaining of the region boxed in Figure 4P). At the level of the youngest formed tentillae, *PpiMHCIIb2* is expressed in a striped pattern, probably corresponding to the nerve cords of each tentilla (Ftt on Figure 4M and N). However, mature tentillae do not show any *PpiMHCIIb2* expression signal, suggesting that *PpiMHCIIb2* is expressed in a sub-population of neural cells, or at a particular stage of neuron differentiation.

### Other sites of *PpiMHCIIa* expression are associated with putative stem cells and proliferating progenitors

**Figure 7:**
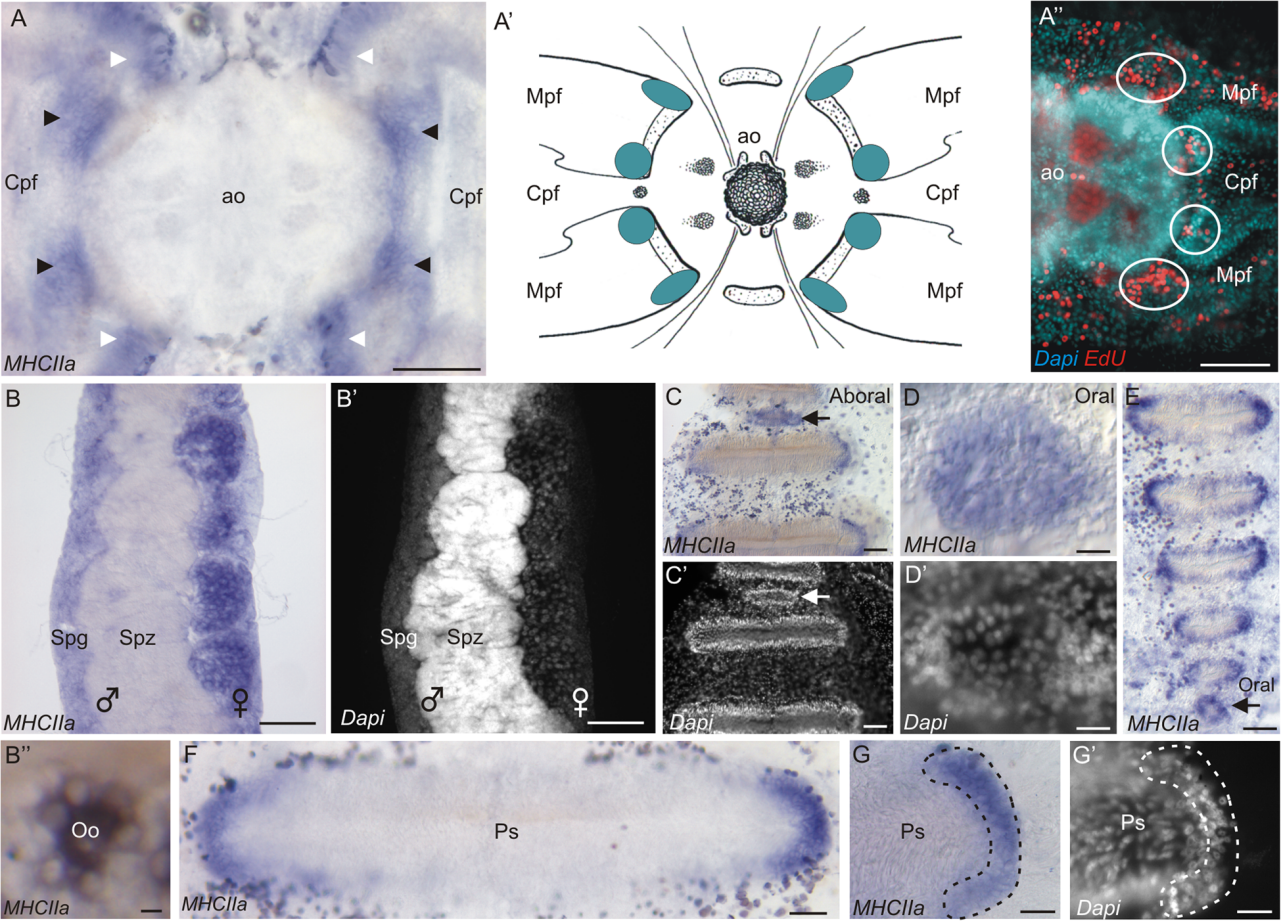
***PpiMHCIIa *expression outside the tentacle root.** (**A**) * PpiMHCIIa * expression in polar fields. Eight patches of *PpiMHCIIa * are identified. The black arrowheads point to the four internal-most patches (thought to correspond to stem cells) and the white arrowheads indicate the 4 external-most patches (thought to correspond to proliferating cells but not stem cells). (**A’**) Summary of the *PpiMHCIIa* expression pattern (blue patches) in polar fields. (**A”**) EdU labelling in the aboral sensory complex after a 12 h pulse and no chase. (**B**) Expression of *PpiMHCIIa* in the gonads (male on the left and female on the right). Spermatozoids (Spz) are not stained. (**B’**) DAPI counter-staining of panel (**B**). (**B”**) Oocyte (Oo) surrounded by its nurse cells, both expressing *PpiMHCIIa*. (**C**) *PpiMHCIIa* expression at the aboral extremity of a comb row (the black arrow points to an aboral baby comb). (**C’**) DAPI counterstaining of panel (**C**). The white arrow points to the aboral baby comb. (**D**) *PpiMHCIIa* expression in the youngest baby comb at the oral extremity of a comb row. (**D’**) DAPI counterstaining of panel (**D**). (**E**) *PpiMHCIIa* expression in the six youngest combs located at the oral extremity of a comb row of another specimen. (**F**) *PpiMHCIIa* expression in a mature comb. (**G**) Detailed view of the lateral extremity of a mature comb showing the localised expression of *PpiMHCIIa*. (**G’**) DAPI counter-staining of panel (**G**). ao: apical organ; Cpf: Central zone of polar field; Mpf: Marginal zones of polar field; Ps: Polster cells; Spg: Spermatogenesis stages; Spz: Spermatozoids. Scale bars: A, A”, C, C’: 50 μm; B, B’: 100 μm; B”: 10 μm; D, D’: 5 μm; E, F: 25 μm; G, G’: 20 μm.

The expression of *PpiMHCIIa* is consistently associated with undifferentiated dividing cells in the adult *Pleurobrachia pileus*. Within the aboral sensory complex, *PpiMHCIIa* is expressed in eight patches of cells at the proximal extremities of the polar field marginal and central zones (Figure 7A and A’). These cells present a high nucleocytoplasmic ratio and are densely packed (data not shown). The four internal-most patches (black arrowheads in Figure 7A) correspond to the four stem cell pools identified by Alié *et al*. [47]. Therefore, *PpiMHCIIa* expression encompasses these stem cell populations but is not restricted to them. In fact, the distribution of *PpiMHCIIa* expression in the aboral sensory complex closely matches the distribution of proliferating cells as revealed by EdU incorporation experiments with a 12-hour pulse and no chase (see Figure 7B’ in [47], and Figure 7A” in this paper).

The *PpiMHCIIa* gene is strongly expressed in both female and male gonads. In *Pleurobrachia pileus*, eight meridional canals located under the eight comb rows contain the gonads in their walls. The male and female germlines are spatially segregated at both sides of each canal. Expression is observed in the entire female gonad (Figure 7B and B’) where young and mature oocytes as well as nurse cells are stained (Figure 7B”). In the male gonad, transcripts are restricted to spermatogenesis stages, in the most peripheral part of the gonad, while the internal-most part of the canal wall (corresponding to mature stages *i.e.* spermatozoids) is not stained (Figure 7B, B’). To conclude, *PpiMHCIIa* is expressed in the germ cells of *Pleurobrachia pileus*, at all stages of oogenesis and during spermatogenesis but not in mature spermatozoids.

Within the comb rows, *PpiMHCIIa* is expressed in the forming combs and at both extremities of the mature combs (Figure 7C-G’). Recognisable by their small size and characteristic position (see [47]), oral and aboral baby combs are entirely stained (Figure 7C, C’, D, D’, E). In mature combs, transcripts are restricted to two crescent shape areas at both extremities of each comb (Figure 7F, G). The observation of DAPI counter-staining indicates that positive cells possess a round nucleus with prominent nucleolus, whereas the unstained poster cells have elongated and twisted nuclei (Figure 7G’). These expression sites correspond to progenitors of the polster cells (ciliated cells of the combs) as identified in a previous work [47].

### *PpiMHCIIb1* is specifically expressed in various kinds of muscle cells

**Figure 8:**
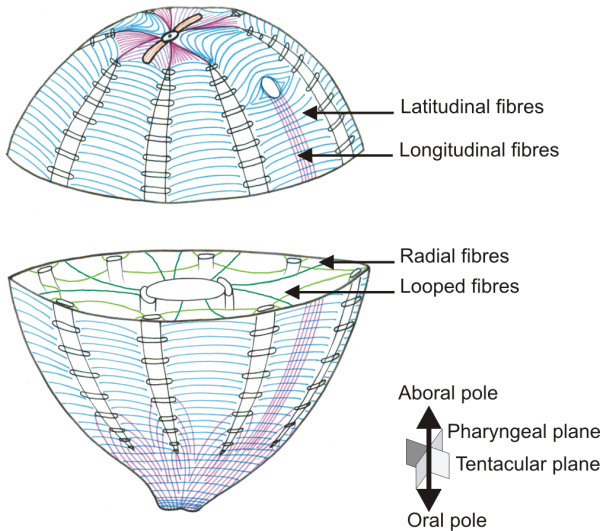
**Arrangement of parietal and a part of mesogleal muscle fibres throughout the *Pleurobrachia pileus *body.** The mesogleal muscle fibres visible on the equatorial plane are represented in green; all other muscle fibres in this drawing are parietal muscle fibres. Among them, latitudinal fibres are in blue and longitudinal fibres are in pink. The tentacle apparatus has been omitted for clarity. See the pictures of phalloidin-stained preparations supporting this drawing in Additional file 6.

*PpiMHCIIb1* is expressed in both kinds of muscle cells described in *Pleurobrachia pileus*: parietal and mesogleal muscle fibres. Using phalloidin-stained preparations, we could reconstruct the complex arrangement of these muscle fibres throughout the *Pleurobrachia pileus* body (Figure 8 and Additional file 6). An important observation (Figure 8) is that the parietal musculature consists mainly of fibres placed in a circular orientation (= latitudinal fibres) and present throughout the body wall, whereas longitudinal parietal fibres are restricted to a few particular regions of the body: (i) aborally, the four areas delineated by the four pairs of adjacent ciliated grooves; (ii) in the tentacular plane, two symmetrical muscle bands extending from each tentacle sheath opening to the mouth region; (iii) in the oral region, the area comprised between the oral extremity of the comb rows and the mouth. At least for the two latter sites, the presence of longitudinal fibres is clearly linked to a functional requirement for strong and relatively rapid contractions allowing changes in mouth orientation and shape during feeding [59]. The arrangement of mesogleal fibres is more complex and is only outlined in Figure 8 for those located in the equatorial plane. These mesogleal fibres can notably connect two adjacent meridional canals (looped fibres on Figure 8 and Additional file 6B), a meridional canal and the external epithelium (Figure 8) or the apical organ and the tentacle sheath (Additional file 6H).

**Figure 9:**
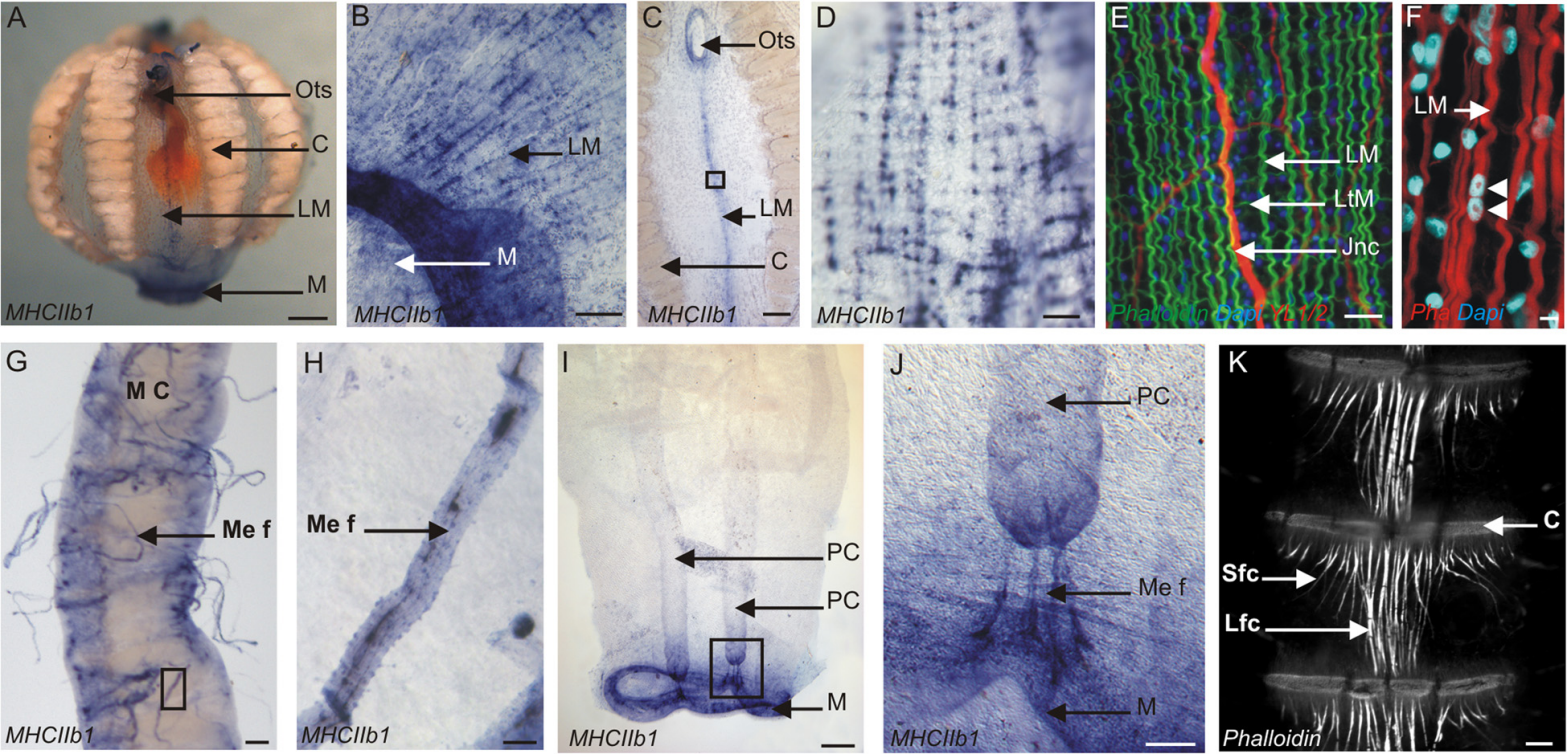
*** PpiMHCIIb1 *expression outside the tentacle root.** (**A**) General view of the *PpiMHCIIb1* expression pattern. (**B**) Detail of the expression of *PpiMHCIIb1* in longitudinal parietal muscle fibres around the mouth. (**C**) Expression of *PpiMHCIIb1* in longitudinal parietal muscle fibres located in the tentacular plane, between the tentacle sheath opening and the mouth. (**D**) Higher magnification of the region boxed in (**C**). (**E**) Phalloidin (in green), YL1/2 (in red) and DAPI (in blue) staining of the same region as shown in panel (**D**) but from another specimen. (**F**) Phalloidin (in red) and DAPI (in blue) staining showing the multinucleation of parietal muscle fibre. (**G**) Expression of *PpiMHCIIb1* in mesogleal muscle fibres (Me F) connected to a meridional canal (M C). (**H**) Higher magnification of one of these *PpiMHCIIb1* expressing mesogleal muscle fibres boxed in (**G**). (**I**) Expression of *PpiMHCIIb1* gene in the mouth rim (**M**) and in mesogleal muscle fibres connecting the two paragastric canals (PC) to the mouth. (**J**) Higher magnification of the region boxed in (**I**) to show the stained mesogleal muscle fibres (Me f) connecting a paragastric canal (PC) to the mouth. (**K**) Portion of a comb row (with 3 successive combs) showing the inter-comb fibres, strongly stained with phalloidin. There are two types of inter-comb fibres: short lateral fibres (Sfc) and long central fibres (Lfc). C: Comb; Jnc: Juxtatentacular nerve cord; Lfc: Long inter-comb fibre cells; LM: Longitudinal muscle fibres; LtM: Latitudinal muscle fibres. M: Mouth; MC: Meridional Canal; Me f: Mesogleal muscle fibre; Ots: Orifice of tentacle sheath; PC: Paragastric canal; Sfc: Short inter-comb fibre cells. Scale bars: A, C, I: 200 μm; B: 100 μm; D, K: 20 μm; E, H: 10 μm; F: 2 μm; G, J: 50 μm.

After a short time of ISH-signal development, *PpiMHCIIb1* expression in the parietal musculature is detected only in longitudinal fibres (Figure 9A, C-D). Notably, *PpiMHCIIb1* is strongly expressed in the two symmetrical muscle bands that run from the tentacle sheath openings to the mouth (Figure 9C-D). A neural condensation called the juxta-tentacular nerve cord [40] is located in the centre of these muscle bands (see YL1/2 immunostaining and phalloidin staining in Figure 9E). There is also strong *PpiMHCIIb1* expression in the numerous short longitudinal fibres that surround the mouth opening (Figure 9B). When the ISH signal is allowed to develop for a longer time, the whole parietal musculature (including the latitudinal fibres throughout the body wall) becomes stained (not shown). The observed difference in terms of staining intensity between latitudinal and longitudinal parietal fibres might reflect either different levels of *PpiMHCIIb1* gene expression, or an optical effect due to the smaller diameter and looser organisation of latitudinal fibres compared to longitudinal ones (well visible in Figure 9E). Our observation of phalloidin and DAPI stained preparations revealed that parietal muscle cells are multinucleated (like the mesogleal muscle cells) (Figure 9F).

*PpiMHCIIb1* is also expressed in mesogleal muscle fibres (Figure 9G-J). Strong *PpiMHCIIb1* staining is notably observed in mesogleal muscle fibres attached to the meridional canals (Figure 9G, H). Some of the *PpiMHCIIb1* expressing mesogleal fibres are shorter and connect the two paragastric canals to the mouth (Figure 9I, J). Phalloidin and DAPI staining confirmed that the mesogleal fibres are multinucleated (with elongated nuclei) (Figure 2F). These observations furthermore indicate that mesogleal muscle fibres are on average much thicker than parietal muscle fibres and are ramified at both extremities where they attach to the epidermis or to the meridional canals (Figure 2F).

Finally, our data on *PpiMHCIIb1* expression provide interesting insights into the nature of an enigmatic type of fibres (here called “inter-comb fibres”) tightly associated with the combs (Figure 9K). These fibres are conspicuously stained with phalloidin and they extend medially from one comb to the following one, with shorter lateral fibres occurring only on the oral side of each comb (Figure 9K). These structures have been observed previously by Chun [49], erroneously interpreted as extensions of the polster cells in *Hormiphora* by Samassa [60], and more recently observed by Hernandez-Nicaise [61]. Although they closely resemble muscle fibres in phalloidin-stained preparations, inter-comb fibres do not show any *PpiMHCIIb1* expression and recent observations suggest that they do not contract following stimulation (L. Moroz, personal communication). Therefore, they are probably not muscular but could represent supporting fibres, possibly maintaining the mechanical cohesion of the comb row against the tension generated by comb beating.

### Outside the tentacle root, *PpiMHCIIb2* is expressed in various types of non-muscle cells

**Figure 10:**
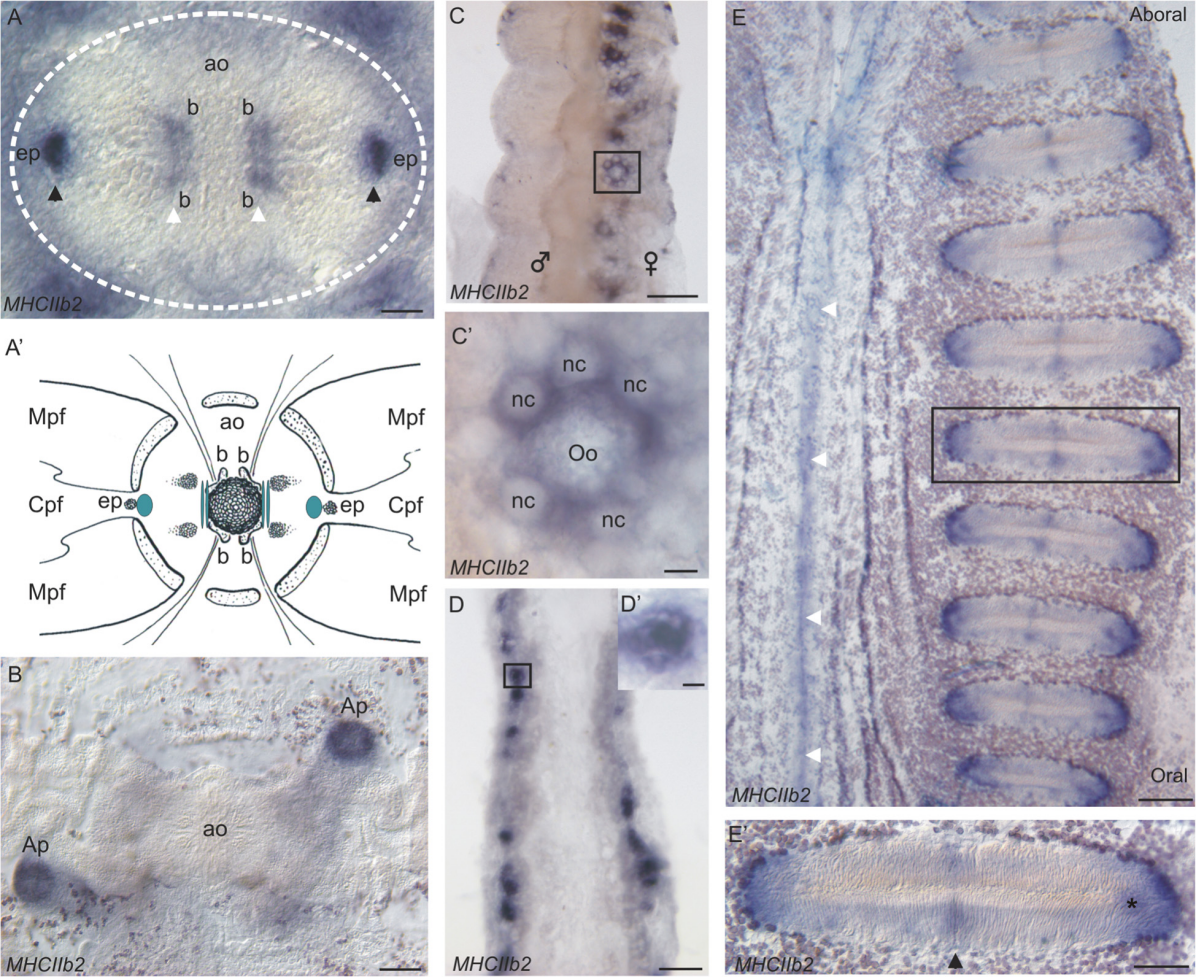
***PpiMHCIIb2 *expression outside the tentacle root.** (**A**) * PpiMHCIIb2 * expression in the apical organ. White arrowheads point to paired structures localised near the balancers (b) and black arrowheads point to paired structures located near the epithelial papillae (ep). (A’) Summary of the *PpiMHCIIb2* expression pattern in the apical organ. (**B**) *PpiMHCIIb2* expression in the anal pores (Ap). (**C**) *PpiMHCIIb2* expression in the female gonad. (**C’**) Higher magnification of the region boxed in (**C**), showing an oocyte (Oo) surrounded by its nurse cells (nc), both expressing *PpiMHCIIb2*. (**D**) Expression of *PpiMHCIIb2* in a paragastric canal. (**E**) Expression of *PpiMHCIIb2* along a comb row and in the neighbouring epithelium. White arrowheads indicate the tentacular plane, where a line of *PpiMHCIIb2* expressing cells is observed. **(E’**) Higher magnification of the region boxed in (**E**). The black arrowhead points to the symmetrical expression spots in the middle of the comb. The star points to the “subterminal spot” of staining near the extremity of the comb. ao: apical organ; Ap: Anal pore; b: balancer cells; Cpf: Central zone of polar field; ep: epithelial papillae Mpf: Marginal zones of polar field; nc: nurse cell; Oo: Oocyte. Scale bars: A: 20 μm; B, D, E’: 50 μm; C, E: 100 μm; C’, D’: 10 μm.

*PpiMHCIIb2* is expressed in different cell types in *Pleurobrachia pileus* but apparently not in muscle cells. Close examination of the floor of the aboral organ reveals the presence of two distinct sites of expression: two peripheral spots close to the epithelial papillae (Figure 10A, black arrowheads; Figure 10A’), and two paired internal stripes, located between the balancers (Figure 10A, white arrowheads; Figure 10A’). Cells surrounding the anal pores are also stained by *PpiMHCIIb2* (Figure 10B).

Moreover, *PpiMHCIIb2* is also expressed in the female gonad (within the body walls of the meridional canals), but here the staining is restricted to growing oocytes and their surrounding nurse cells (Figure 10C-C’) unlike *PpiMHCIIa* which is expressed throughout the ovary (Figure 7B). Oocyte and nurse cells are known to belong to the same cell lineage [33,62,63]. Another site of *PpiMHCIIb2* expression are the paragastric canals, where staining is restricted to particular clusters of cells (of unknown nature) on the edges of the canals (Figure 10D).

In addition, *PpiMHCIIb2* has a complex expression pattern within mature combs, in (i) both lateral extremities of the comb (Figure 10E, E’), which might reflect *PpiMHCIIb2* expression in polster cell stem cells/progenitors, (ii) a subterminal spot (star in Figure 10E’) and (iii) two small symmetrical groups of cells in the centre of each comb (Figure 10E’, black arrowhead). These latter two cell sub-populations have not been previously distinguished morphologically. A last expression site of *PpiMHCIIb2* is observed in the ectodermal epithelium along the tentacular plane (white arrowheads in Figure 10E). This stripe of *PpiMHCIIb2* expression is located close to the juxtatentacular nerve cord and of the longitudinal muscle expressing *PpiMHCIIb1* and *PpiTropomyosin* (Figure 9C-E). However, the *PpiMHCIIb2*-expressing cells in this region are not muscle cells but round-shape epithelial cells of unclear nature (data not shown).

## Discussion and conclusions

### Conserved expression of *MHCIIa* genes in the germ line, in stem cells and in proliferating cells

The bilaterian orthologues of *PpiMHCIIa* are expressed in smooth muscle cells or in non-muscle cells [64-67]. Some of the vertebrate members of this group of myosin II are classically known for their role in the contractile activity of smooth muscle cells, but other members are essential for mitosis, in all cell lineages, being involved in the formation of the contractile ring during cytokinesis. Recent findings demonstrate that smooth/non-muscle myosin II also play an active role in various other cellular activities such as generation of cell polarity, cell migration and cell-cell adhesion (for a review see [11]). Moreover, a recent work based on specific inhibition of non-muscle myosins II demonstrated their involvement in self-renewal programmes in human and mouse pluripotent stem cells [68].

In *Pleurobrachia pileus,* the expression pattern of *PpiMHCIIa* is strikingly similar to that of the “stem cell markers”: *VasaPiwiBruno* and *PL10*[47] and the *SOX* genes *PpiSOX2* and *PpiSOX12*[55]. Its expression territories include the male and female gonads and several spatially restricted pools of stem cells / progenitors involved in somatic cell renewal, within the comb plates, aboral sensory complex and tentacle root. Expression of *PpiMHCIIa* throughout the ctenophore germ line (except in the most mature stages of spermatogenesis) suggests a conservative role for non-muscle myosin II (MHCIIa clade) in gametogenesis, as proposed in different studies of vertebrate meiotic chromosomes [69,70]. In somatic territories, *PpiMHCIIa* is consistently expressed in the various populations of stem cells that were recently identified in the adult ctenophore using a combination of gene expression data and DNA-label incorporation and long-term retention assays [47]. This is particularly obvious in the tentacle root, where *PpiMHCIIa* expression is restricted to the three parallel ridges known to house stem cells. It is furthermore significant that *PpiMHCIIa* expression in the tentacle root is not limited to muscle stem cells (located in the median ridge) but also includes the colloblast stem cells (in the lateral ridges), suggesting a link with stemness/undifferentiated state/cell proliferation rather than with muscle cell identity. This conclusion finds additional support from *PpiMHCIIa* expression in stem cell pools located at both extremities of the combs (and involved in renewal of the ciliated “polster cells”) as well as in the four internal stem cell pools previously identified around the apical organ.

When examined in detail, some aspects of the *PpiMHCIIa* expression pattern further suggest that beyond stem cells, this myosin is associated with cell proliferation, including in progenitors that are already committed to undergo differentiation. In the aboral sensory complex, after a 12 h EdU pulse and no chase, eight discrete patches of nuclei are labelled around the apical organ (in the proximal region of the polar fields) (Figure 7C-E” in [47] and Figure 7A” in this paper). When a 5-day chase is added after the pulse, only four of these patches remain labelled. A likely explanation for this observation is that cells of the four other patches have become differentiated and therefore were proliferating progenitors but not stem cells. Whereas genes like *Piwi* and *Vasa* tend to have their transcripts highly concentrated in the four stem cell patches [47], *PpiMHCIIa* is equally expressed in eight patches whose position matches the eight labelled cell groups obtained after EdU pulse and no chase. *PpiMHCIIa* expression might also extend to proliferating progenitors in other tissues (tentacle root, combs) but the continuous spatial gradient of cell differentiation from stem cells in these tissues makes it more difficult to distinguish expression in stem cells vs. in progenitors. The suggestion that *PpiMHCIIa* expression is associated with all kinds of proliferating cells in *Pleurobrachia pileus* is consistent with its involvement in the movements of the contractile ring upon cytokinesis in bilaterian models.

Finally, whereas vertebrate smooth muscle cells express *MHCIIa* genes [64-67], in ctenophore *PpiMHCIIa* is apparently not expressed in muscle cells, even if all of them are said to be of the smooth type [31]. The only case of *PpiMHCIIa* expression in a muscle cell lineage concerns the muscle progenitors along the median ridge of the tentacle root, but as explained above this has probably nothing to do with muscular identity but rather with stemness and/or cell proliferation.

### Independent functional specialisation of MHCII myosins and the possible convergent origin of ctenophore muscles

At least some of the non-muscular functions played by MHCIIa proteins are certainly very ancient since myosins are cellular motors involved in various cellular processes (e.g. cytokinesis) throughout eukaryotes including members of unicellular lineages [71,72]. The MHCIIa/MHCIIb duplication predated the divergence of sponges and placozoans and therefore occurred well before the origin of muscle cells, implying that both MHCII subfamilies were initially working in a non-muscular context. The ctenophore data furthermore suggest that *MHCIIa* genes were not involved in muscle contraction but had retained the plesiomorphic non-muscular expression in the last common ancestor of ctenophores and bilaterians, although muscle cells are generally thought to have been present in this ancestor ( [37,73]. Expression of some MHCIIa myosins in the smooth muscle cells of vertebrates is certainly the result of a late co-option event, in consistence with the idea that vertebrate smooth muscle cells were independently evolved [1,24]. Accordingly, in scallop (a mollusc) smooth and striated muscles of the foot express two different splicing variants of the same MHCII gene, closely related to vertebrate striated muscle myosin II (MHCIIb) [74], indicating that functional specialisation of myosins according to muscle cell types (smooth *vs.* striated) occurred independently through different paths in different bilaterian lineages.

The expression of one of the ctenophore MHCIIb paralogues, *PpiMHCIIb2,* shows several striking similarities to that of *PpiMHCIIa* and other metazoan myosins of the MHCIIa subfamily. First, in contrast to the other paralogue *PpiMHCIIb1* (expressed in muscle cells of any kind)*, PpiMHCIIb2* has strictly non-muscular expression. Like *PpiMHCIIa,* this gene is expressed in putative stem cells and/or progenitors cells at both extremities of the comb rows, and also in the germ line. Here *PpiMHCIIb2* is expressed in a more restricted territory than *PpiMHCIIa* (i.e. only within large oocytes and their surrounding nurse cells in the ovary). This might reflect functional specialisation between *PpiMHCIIa* and *PpiMHCIIb2* during gametogenesis, perhaps in cell proliferation for the former *vs.* formation of the cytoplasmic bridges that link the oocyte and the nurse cells (both deriving from common precursors [33,63]) and/or cytoplasmic transport from the nurse cells to the oocyte, as documented for non-muscle myosin II in *Drosophila* oogenesis [75,76], for the latter. Additionally, distinct subpopulations of nerve cells express *PpiMHCIIb2* in at least two regions of the body: a nerve plexus in the symmetry plane of the tentacle root, and several territories in the epithelial floor of the apical organ, exactly matching the previously-reported distribution of anti-vasopressin immunoreactive neuro-sensory cells (see Figure 6D-F in [40]). This neural expression of *PpiMHCIIb2* reminds the involvement of some *MHCIIa* genes in the nervous system of various vertebrates and *Drosophila*, where they promote synaptic vesicle assembly and movements through interactions with F-actin partners, neuronal migration, neuronal morphogenesis and growth cone motility [77-80].

The apparently odd MHCIIa-like expression pattern of *PpiMHCIIb2* can be easily explained if ctenophore muscle cells evolved independently from those of cnidarians and bilaterians. Under this scenario, when the ctenophore lineage originated, the two single-copy genes *MHCIIa* and *MHCIIb* would have still retained the plesiomorphic non-muscular functions.

Then *MHCIIb* duplicated in ctenophores, and one of the resulting parologues (*PpiMHCIIb1*) underwent neofunctionalisation in the context of a newly-evolved type of contractile fibre cell. Yet it is possible to imagine alternative scenarios, not assuming independent muscle cell origins. If muscle cells emerged only once in a eumetazoan ancestor with MHCIIb correlatively becoming exclusively muscular, then the observed non-muscular expression of ctenophore *PpiMHCIIb2* must be interpreted as derived, and we would face a unique and rather weird instance of a formerly muscular myosin having shifted to non-muscular function. Furthermore, in that case, shared expression features between *PpiMHCIIb2* and *PpiMHCIIa* find no explanation and must be considered fortuitous. A last plausible scenario assumes that in the last common ancestor of Eumetazoa, MHCIIb had pleiotropic expression in both non-muscle cells (plesiomorphic role) and newly acquired muscle cells (apomorphic role). Then bilaterians lost the non-muscular MHCIIb expression, whereas in the ctenophore lineage the MHCIIb duplication was followed by functional specialisation of the two copies, one taking over the muscular function and the other the non-muscular roles, a scenario conforming to the duplication-degeneration-complementation model [81]. It is finally quite clear that, albeit non-parsimonious with respect to the taxon phylogeny, the hypothesis of convergent muscle origins offers the most straightforward explanation to the observed ctenophore myosin expression patterns.

It is nevertheless important to underline that data from cnidarians will be essential to evaluate these scenarios but are insufficient at the moment. In the hydromedusa *Podocoryne carnea*, a MHCIIb group gene seems to be specifically expressed in the striated muscle layer of the medusa, located on the inner side of the bell [82], but expression data for cnidarian MCHIIa genes are critically lacking.

### The originality and interest of ctenophores for studying muscle differentiation

A first striking characteristic of the ctenophore musculature is the diversity of muscle cell types and the complexity of their spatial arrangement (see Figure 8), as exemplified by our TEM observations combined with expression analyses of the *MHCIIb1* gene and phalloidin staining. The existence in ctenophore of two fundamentally distinct musculatures, epithelial (*i.e.* parietal muscles) and mesogleal, parallels the similar distinction recently confirmed using immunofluorescence [40] for the nervous system, and represents a fundamental difference with cnidarians. While the later are fundamentally epithelial animals, ctenophores have a fully developed mesogleal compartment with proper nervous and muscle systems, a condition more alike that of the triploblastic bilaterians. Furthermore in ctenophores, within each of the two main muscle categories, there is a significant diversity of muscle fibre subtypes differing by their size, position within the body, orientation, organisation and mode of formation (documented only for the tentacle root).

Among these ctenophore muscles, those of the tentacular apparatus (comprised of the tentacle and its attached tentillae) represent an autonomous muscle system not only from a functional point of view, but most importantly in terms of ontogeny and cell lineages. The ctenophore tentacle root is a highly promising model for studying cell differentiation and particularly myogenesis. Indeed, it is the only site of muscle cell production within the body where progenitors are easily identifiable and thereby are amenable to experimental approaches, whereas for parietal muscle cells and for other mesogleal muscle cell, we do not know where are the reservoirs of stem cells and progenitors. TEM observations and histological cryosections, combined with expression analyses of the three *MHCII* and *Tropomyosin* genes lead us to propose an original myogenic model implying spatially ordered regionalisation of muscle cell differentiation stages (Figure 6). This model is furthermore consistent with recent analyses of the progenies of somatic stem cells based on stem cell gene expression and EdU DNA labelling and long-term retention [47]. Whereas the main axis (median expansion) of the tentacle root is essentially a centre of muscle production, the two lateral expansions are mostly dedicated to the differentiation and maturation of colloblasts (adhesive cells), a ctenophore-specific cell type [31,47,48]. At the oral pole of the tentacle root, we identified an additional and independent centre, responsible for the production of tentilla muscles. Thus, the tentacle root contains at least three autonomous cell lineages, each with its particular niche of stem cells (note that the “aboral external cell masses” described in [47], probably represent a fourth cell lineage, of unknown identity).

The high renewal rate of tentacle cells (with complete tentacle regeneration in 36 hours [46]) makes this system highly suitable not only for studying regeneration processes but also for analysing the role of key regulators, such as myogenic genes, yet unknown in ctenophores. The spatial segregation of muscle cell stages in the tentacle root in transverse section should facilitate the identification of genes involved in the various steps of myogenesis. Therefore, the tentacle root is a promising experimental system to study stem cell regulation, cell specification and cell differentiation in ctenophores.

### The limits of the “smooth” *vs.* “striated” classification of muscle cell types

The mode of muscle cell differentiation described here in the ctenophore tentacle root is strikingly similar to that of striated skeletal muscles in vertebrates. In both cases, muscle development passes through four main stages: multiplication of myoblasts, alignment, fusion into myotubes, and finally maturation of the multinucleated muscle cells. In the ctenophore tentacle root, these myogenesis stages are distributed in four distinct areas along the proximal-distal axis on both sides of the tentacle root symmetry plane (Figure 6). A different mechanism operates in vertebrates for smooth muscle cells, which are mononucleated and whose formation does not involve a phenomenon of myoblast fusion. It is therefore surprising that although ctenophore muscle cells are of the smooth type (they lack recognisable sarcomeres), their multinucleate condition and their mode of development is more alike vertebrate striated muscle cells than vertebrate smooth muscle cells. Expression of *MHCIIb* is another shared characteristic of ctenophore muscle cells and vertebrate striated muscle cells. The evolutionary significance of these observations remains unclear: ctenophore muscles could have acquired these characteristics by convergence, or the similarities might reflect ancestral features of eumetazoan muscle cells, consistent with a scenario suggested previously in which smooth vertebrate muscle cells would be a derived (vertebrate-specific) cell type [1,83].

In any case, these observations imply that a classification of muscle cell types based solely on presence or absence of a striation pattern (*i.e.* of Z discs allowing transverse or oblique alignment of the myofilaments) captures only part of the variation existing among animal muscle cells. A more comprehensive classification of muscle cell types at the eumetazoan taxonomic scale therefore remains to be elaborated, by integrating not only cytological characteristics but also molecular and developmental features. In this perspective, it will be crucial to obtain data from a wide sampling of early-branching metazoans concerning the expression and function of myogenic and muscle differentiation genes, including the various forms of myosins.

## Authors’ contributions

The work presented here was carried out in collaboration between all authors. CD performed the experiments, analysed the data and drafted the paper. EQ and MM designed analytical strategies and revised the paper. AA, PC and CD carried out the TEM investigations. MJ assisted CD in the *in-situ* hybridisation and immunofluorescence experiments and revised manuscript. HLG provided all facilities. All authors have read and approved the final manuscript.

## Supplementary Material

Additional file 1 **Alignment of myosin amino-acid sequences.** This file contains the initial alignment (before site pruning by Gblocks) in graphic view. Note that for myosins of classes other than class II (outgroups), only the sequences of the motor domain have been included (the C-terminal part of these proteins is not alignable with the C-terminal part of myosins II). For *Saccaromyces cerevisiae* MHCII, the portion of the protein sequence downstream from position 1300 has been likewise omitted for the same reason. Click here for file

Additional file 2 **Final sequence alignment (after Gblocks) including non-class II myosins.** The corresponding tree is shown in Additional file 3 (see Methods for details). The alignment is provided in Phylip 4 format. Click here for file

Additional file 3 **Phylogenetic analysis rooted on non-class II myosins.** Amino-acid sequences were analysed using the Maximum likelihood (ML) method. Numbers associated with the branches are ML bootstrap values (1000 replicates). Sequences from *Pleurobrachia pileus* are indicated in bold and green. Branches within the MHCIIa clade are in blue and within the MHCIIb clade in purple. The tree was rooted with sequences of myosin classes V, VII and X (outgroups). The letter between parentheses after the name of mouse genes of the MHCa clade indicate whether the gene is expressed in non-muscle cells (n) or in smooth muscle cells (s). The *PpiMHCIIb2* gene was excluded because its partial sequence contains only the tail and therefore it has no alignable residues with non-class II myosins (only the head being homologous between class II and non-class II myosins). Abbreviations for species names: Aae: *Aedes aegypti*; Aqu: *Amphimedon queenslandica*; Asu: *Ascaris suum*; Cow: *Capsaspora owczarzaki*; Ddi: *Dictyostelium discoideum*; Dme: *Drosophila melanogaster*; Hma: *Hydra magnipapillata*; Lgi: *Lottia gigantea*; Lpe: *Loligo pealei*; Mbr: *Monosiga brevicollis*; Mmu: *Mus musculus*; Nve: *Nematostella vectensis*; Ppi: *Pleurobrachia pileus*; Sar: *Sphaeroforma arctica*; Sce: *Saccharomyces cerevisiae*; Sro: *Salpingoeca rosetta*; Tad: *Trichoplax adhaerens*. Click here for file

Additional file 4 **Final sequence alignment (after Gblocks) including only class II myosins.** The corresponding tree is shown in Figure 3 (see Methods for details). The alignment is provided in Phylip 4 format. Click here for file

Additional file 5 **Expression pattern of *PpiTropomyosin *gene in *Pleurobrachia pileus *muscles.** (A) Internal view of a dissected tentacle root stained with the *PpiTropomyosin* antisense probe (CU420922.1). (B) Longitudinal view of the tentacle root (after removal of lateral expansions) showing *PpiTropomyosin* expression in tentacle and tentilla muscle progenitors. Forming tentillae are also stained. (C) Transverse cryosection (according to the dotted line on (B)) of a tentacle root after *PpiTropomyosin* whole-mount ISH. The tentacle base is also sectioned transversally (Tcl). Note that the musculature of the tentacle sheath lining is also stained (TS). (D) Transverse cryosection of a tentilla after *PpiTropomyosin* whole-mount ISH (compare with Figure 4V). (E) Expression of *PpiTropomyosin* in longitudinal muscle fibres (LM) in the tentacular plane. (F) Expression of *PpiTropomyosin* in mesogleal muscle fibres connected to a meridional canal. (G) Higher magnification of *PpiTropomyosin* expression in a mesogleal muscle fibre. C: Comb; Coll: Colloblasts; F tt: Forming tentillae; LM: Longitudinal Muscle fibres; MC: Meridional Canal; Me f: Mesogleal muscle fibre; MR: Median Ridge; M tcl: Tentacle Muscle progenitors; M tt: Tentilla Muscles progenitors; Mu: Muscle fibres; Ots: Orifice of tentacle sheath; Tcl: Tentacle; TS: Tentacular Sheath; Tt: Tentilla. Scale bars: A, B, C, E: 200 μm; F: 100 μm; D: 25 μm; G: 10 μm. Click here for file

Additional file 6 ***Pleurobrachia pileus *musculature (phalloidin staining).** (A) Drawing of the arrangement of *Pleurobrachia pileus* musculature, with areas corresponding to pictures (B-I) indicated by black boxes. (B-J) Phalloidin staining of muscle fibres in selected regions of the body (boxes in (A)). (B) A large mesogleal muscle fibre connecting two meridional canals. Inter-comb fibre cells are also visible. (C) Dense parietal muscle fibres along a ciliated groove. (D) Parietal muscle fibres in the aboral region, in the pharyngeal plane. (E) Parietal muscle fibres in the aboral region, in the tentacular plane. (F) Parietal muscle fibres in the epidermis between two comb rows. All fibres have circular orientation. (G) Parietal muscles fibres in the epidermis between two comb rows in the tentacular plane: a dense band of longitudinal fibres is superimposed on the circular fibres. (H) Epithelial muscle fibres around the opening of the tentacle sheath. Note that some mesogleal fibres are visible connecting the apical organ area to the tentacle sheath. (I) Epithelial muscle fibres around the oral extremity of the comb row. (J) Higher magnification of epithelial muscles fibres at the oral extremity of the comb row. The light coloured structure visible at the top of the picture is the oral extremity of the meridional canal underlying the comb row. C: Comb; Cg: Ciliated groove; CR: Comb row; Fi C: inter-comb fibrous cells; M C: Meridional canal; Me f: Mesogleal muscle fibre; Ots: Opening of tentacle sheath. Scale bars: B-J: 100 μm. Click here for file

## References

[B1] Goodson HV Spudich JA Molecular evolution of the myosin family: relationships derived from comparisons of amino acid sequences Proc Natl Acad Sci USA 1993 90 2 659 663 10.1073/pnas.90.2.659 8421702 PMC45723

[B2] Hodge T Cope MJ A myosin family tree J Cell Sci 2000 19 3353 3354 10984423 10.1242/jcs.113.19.3353

[B3] Thompson RF Langford GM Myosin superfamily evolutionary history Anat Rec 2002 268 276 289 10.1002/ar.10160 12382324

[B4] Odronitz F Kollmar M Drawing the tree of eukaryotic life based on the analysis of 2,269 manually annoted myosins from 328 species Gen Biol 2007 8 R196.1 23 10.1186/gb-2007-8-9-r196 PMC2375034 17877792

[B5] Richards TA Cavalier-Smith T Myosin domain evolution and the primary divergence of eukaryotes Nature 2005 436 7054 1113 1118 10.1038/nature03949 16121172

[B6] Foth BJ Goedecke MC Soldati D New insights into myosin evolution and classification Proc Natl Acad Sci USA 2006 103 10 3681 3686 10.1073/pnas.0506307103 16505385 PMC1533776

[B7] Meili R Alonso-Latorre B del Alamo JC Firtel RA Lasheras JC Myosin II is essential for the spatiotemporal organization of traction forces during cell motility Mol Biol Cell 2010 21 3 405 417 10.1091/mbc.E09-08-0703 19955212 PMC2814786

[B8] Maciver SK Myosin II function in non-muscle cells Bioessays 1996 18 3 179 182 10.1002/bies.950180304 8867731

[B9] Burgess DR Cytokinesis: new roles for myosin Curr Biol 2005 15 8 R310 311 10.1016/j.cub.2005.04.008 15854900

[B10] Even-Ram S Doyle AD Conti MA Matsumoto K Adelstein RS Yamada KM Myosin IIA regulates cell motility and actomyosin-microtubule crosstalk Nat Cell Biol 2007 9 3 299 309 Erratum in: Nat Cell Biol 2007, 9(4):480 10.1038/ncb1540 17310241

[B11] Conti MA Adelstein RS Nonmuscle myosin II moves in new directions J Cell Sci 2007 121 11 18 18096687 10.1242/jcs.007112

[B12] Vicente-Manzanares M Ma X Adelstein RS Horwitz AR Non-muscle myosin II takes centre stage in cell adhesion and migration Nat Rev Mol Cell Biol 2009 10 11 778 790 10.1038/nrm2786 19851336 PMC2834236

[B13] Ivanov AI Bachar M Babbin BA Adelstein RS Nusrat A Parkos CA A unique role for nonmuscle myosin heavy chain IIA in regulation of epithelial apical junctions PLoS One 2007 2 7 e658 17668046 10.1371/journal.pone.0000658 PMC1920554

[B14] Guo S Kemphues KJ A non muscle myosin required for embryonic polarity in Caenorhabditis elegans Nature 1996 382 455 458 10.1038/382455a0 8684486

[B15] Burgess DR Cytokinesis and the establishment of early embryonic cell polarity Biochem Soc Trans 2008 36 Pt 3 384 386 18481964 10.1042/BST0360384

[B16] Franke JD Montague RA Kiehart DP Nonmuscle myosin II generates forces that transmit tension and drive contraction in multiple tissues during dorsal closure Curr Biol 2005 15 24 2208 2221 10.1016/j.cub.2005.11.064 16360683

[B17] Martin AC Kaschube M Wieschaus EF Pulsed contractions of an actin-myosin network drive apical constriction Nature 2009 457 7228 495 499 10.1038/nature07522 19029882 PMC2822715

[B18] Martin AC Pulsation and stabilization: contractile forces that underlie morphogenesis Dev Biol 2010 341 1 114 125 10.1016/j.ydbio.2009.10.031 19874815

[B19] Matsumura F Regulation of myosin II during cytokinesis in higher eukaryotes Trends Cell Biol 2005 15 7 371 377 10.1016/j.tcb.2005.05.004 15935670

[B20] McBeath R Pirone DM Nelson CM Bhadriraju K Chen CS Cell shape, cytoskeletal tension, and RhoA regulate stem cell lineage commitment Dev Cell 2004 6 4 483 495 10.1016/S1534-5807(04)00075-9 15068789

[B21] Engler AJ Sen S Sweeney HL Discher DE Matrix elasticity directs stem cell lineage specification Cell 2006 126 4 677 689 10.1016/j.cell.2006.06.044 16923388

[B22] Clark K Langeslag M Figdor CG Van Leeuwen FN Myosin II and mechanotransduction: a balancing act Trends Cell Biol 2007 17 4 178 186 10.1016/j.tcb.2007.02.002 17320396

[B23] Sellers JR Myosins: a diverse superfamily Biochim Biophys Acta 2000 1496 1 3 22 10.1016/S0167-4889(00)00005-7 10722873

[B24] Korn ED Coevolution of head, neck, and tail domains of myosin heavy chains Proc Natl Acad Sci U S A 2000 97 23 12559 12564 10.1073/pnas.230441597 11058170 PMC18803

[B25] Arendt D The evolution of cell types in animals: emerging principles from molecular studies Nat Rev Genet 2008 9 11 868 882 10.1038/nrg2416 18927580

[B26] Brusca RC Brusca GJ Invertebrates. Sinauer Associated, Inc. (second edition) 2005 1 1

[B27] Chapman DM Microanatomy of the cubopolyp, Trypedalia cystophora (Class Cubozoa) Helgoländer wiss Meeresunters 1978 31 128 148 10.1007/BF02296994

[B28] Chapman DM Microanatomy of the bell rim of Aurelia aurita (cnidaria, Scyphozoa) Can J Zool 1999 77 34 46

[B29] Seipel K Schmid V Evolution of striated muscle; Jellyfish and the origin of triploblasty Dev Biol 2005 282 14 26 10.1016/j.ydbio.2005.03.032 15936326

[B30] Seipel K Schmid V Mesodermal anatomies in cnidarian polyps and medusae Int J Dev Biol 2006 50 589 599 10.1387/ijdb.062150ks 16892172

[B31] Hernandez-Nicaise ML Harrison FW, Westfall JA Ctenophora Microscopic Anatomy of the Invertebrates. Vol. Volume II: Placozoa, Porifera, Cnidaria, and Ctenophora 1991 New York: John Wiley 359 418

[B32] Rieger RM Ladurner P The significance of muscle cells for the origins of mesoderm in Bilateria Integr Comp Biol 2003 43 47 54 10.1093/icb/43.1.47 21680408

[B33] Martindale MQ Henry JQ Gilbert SF, Raunio AM Ctenophorans, the Comb Jellies Embryology: constructing the organism, Sinauer, (Sunderland, MA) 1997 87 111

[B34] Martindale MQ Henry JQ Intracellular fate mapping in a basal metazoan, the ctenophore Mnemiopsis leidyi, reveals the origins of mesoderm and the existence of indeterminate cell lineages Dev Biol 1999 214 243 257 10.1006/dbio.1999.9427 10525332

[B35] Dunn CW Hejnol A Matus DQ Pang K Browne WE Smith SA Seaver E Rouse GW Obst M Edgecombe GD Sørensen MV Haddock SH Schmidt-Rhaesa A Okusu A Kristensen RM Wheeler WC Martindale MQ Giribet G Broad phylogenomic sampling improves resolution of the animal tree of life Nature 2008 452 745 749 10.1038/nature06614 18322464

[B36] Hejnol A Obst M Stamatakis A Ott M Rouse GW Edgecombe GD Martinez P Baguñà J Bailly X Jondelius U Wiens M Müller WE Seaver E Wheeler WC Martindale MQ Giribet G Dunn CW Assessing the root of bilaterian animals with scalable phylogenomic methods Proc Biol Sci 2009 276 1677 4261 4270 10.1098/rspb.2009.0896 19759036 PMC2817096

[B37] Philippe H Derelle R Lopez P Pick K Borchiellini C Boury-Esnault N Vacelet J Renard E Houliston E Quéinnec E Da Silva C Wincker P Le Guyader H Leys S Jackson DJ Schreiber F Erpenbeck D Morgenstern B Wörheide G Manuel M Phylogenomics revives traditional views on deep animal relationships Curr Biol 2009 10 106 113 10.1016/j.cub.2009.02.052 19345102

[B38] Philippe H Brinkmann H Lavrov DV Littlewood DT Manuel M Wörheide G Baurain D Resolving difficult phylogenetic questions: why more sequences are not enough PLoS Biol 2011 9 3 e1000602 10.1371/journal.pbio.1000602 21423652 PMC3057953

[B39] Pick KS Philippe H Schreiber F Erpenbeck D Jackson DJ Wrede P Wiens M Alié A Morgenstern B Manuel M Wörheide G Improved phylogenomic taxon sampling noticeably affects nonbilaterian relationships Mol Biol Evol 2010 27 9 1983 1987 10.1093/molbev/msq089 20378579 PMC2922619

[B40] Jager M Chiori R Alié A Dayraud C Quéinnec E Manuel M New insights on ctenophore neural anatomy: Immunofluorescence study in Pleurobrachia pileus (Müller, 1776) J Exp Zool B Mol Dev Evol 2011 316B 3 171 187 10.1002/jez.b.21386 21462312

[B41] Hernandez-Nicaise ML Amsellem J Ultrastructure of the giant smooth muscle fiber of the ctenophore Beroe ovata J Ultrastruct Res 1980 72 2 151 68 10.1016/S0022-5320(80)90053-2 7191446

[B42] Hernandez-Nicaise ML Mackie GO Meech RW Giant smooth muscle cells of Beroe. Ultrastructure, innervation, and electrical properties J Gen Physiol 1980 75 1 79 105 10.1085/jgp.75.1.79 6102109 PMC2215185

[B43] Hernandez-Nicaise ML Bilbaut A Malaval L Nicaise G Isolation of functional giant smooth muscle cells from an invertebrate: structural features of relaxed and contracted fibers Proc Natl Acad Sci USA 1982 79 6 1884 1888 10.1073/pnas.79.6.1884 6952237 PMC346085

[B44] Bilbaut A Hernandez-Nicaise ML Leech CA Meech RW Membrane currents that govern smooth muscle contraction in a ctenophore Nature 1988 331 6156 533 535 10.1038/331533a0 2448648

[B45] Mackie GO Mills CE Singla CL Structure and function of the prehensile tentilla of Euplokamis (Ctenophora, Cydippida) Zoomorphology 1988 107 319 337 10.1007/BF00312216

[B46] Hernandez-Nicaise ML Franc JM Grassé PP Embranchement des Cténaires Traité de Zoologie 1994 943 1075

[B47] Alié A Leclère L Jager M Dayraud C Chang P Le Guyader H Quéinnec E Manuel M Somatic stem cells express Piwi and Vasa genes in an adult ctenophore: ancient association of “germline genes” with stemness Dev Biol 2011 350 183 197 10.1016/j.ydbio.2010.10.019 21036163

[B48] Hertwig R Ueber den Bau der Ctenophoren Jena Z Naturwiss 1880 14 393 457

[B49] Chun C Die Ctenophoren des Golfes von Neapel und der angrenzenden Meeres-Abschnitte: eine Monographie 1880 Leipzig: Verlag von W. Engelmann 1 311

[B50] Benwitz G Elektronenmikroskopische Untersuchung der Colloblasten-Entwicklung bei der Ctenophore Pleurobrachia pileus (Tentaculifera, Cydippea) Zoomorphologie 1978 89 257 278 10.1007/BF00993952

[B51] Franc JM La mésoglée des cténaires: approches ultrastructurale, biochimique et métabolique PhD Thesis. University Claude Bernard (Lyon I) 1985

[B52] Edgar RC MUSCLE: multiple sequence alignment with high accuracy and high throughput Nucleic Acids Res 2004 32 5 1792 1797 10.1093/nar/gkh340 15034147 PMC390337

[B53] Castresana J Selection of conserved blocks from multiple alignments for their use in phylogenetic analysis Mol Biol Evol 2000 17 540 552 10.1093/oxfordjournals.molbev.a026334 10742046

[B54] Guindon S Gascuel O A simple, fast, and accurate algorithm to estimate large phylogenies by maximum likelihood Syst Biol 2003 52 5 696 704 10.1080/10635150390235520 14530136

[B55] Jager M Quéinnec E Chiori R Le Guyader H Manuel M Insights into the early evolution of SOX genes from expression analyses in a ctenophore J Exp Zoolog B Mol Dev Evol 2008 310B 650 667 10.1002/jez.b.21244 18942104

[B56] Chehrehasa F EdU, a new thymidine analogue for labelling proliferating cells in the nervous system J Neurosci Methods 2009 177 122 10.1016/j.jneumeth.2008.10.006 18996411

[B57] Eisenman EA Alfert M A new fixation procedure for preserving the ultrastructure of marine invertebrate tissues J Microsc 1981 125 117 120

[B58] Sun MG Williams J Munoz-Pinedo C Perkins GA Brown JM Ellisman MH Green DR Frey TG Correlated three-dimensional light and electron microscopy reveals transformation of mitochondria during apoptosis Nat Cell Biol 2007 9 1057 1065 10.1038/ncb1630 17721514

[B59] Tamm SL Shelton GAB Ctenophora Electrical Conduction and Behaviour in "Simple" Invertebrates 1982 Oxford: Clarendon Press 266 358

[B60] Samassa P Zur Histologie der Ctenophoren Arch mikr Anat 1892 40 157 242 10.1007/BF02954492

[B61] Hernandez-Nicaise ML Système nerveux et intégration chez les Cténaires. Etude ultrastructurale et comportementale Ph. D. Thesis. University Claude Bernard (Lyon I) 1974

[B62] Dunlap HL Oogenesis in the Ctenophora Ph.D. Thesis. University of Washington (Seattle, Washington) 1966

[B63] Dunlap-Pianka HL Giese AC, Pearse JS Ctenophora Reproduction of Marine Invertebrates 1974 New-York: Academic Press 201 211

[B64] Kelley CA Characterization of isoform diversity among smooth muscle and nonmuscle myosin heavy chains Comp Biochem Physiol B Biochem Mol Biol 1997 117 1 39 49 10.1016/S0305-0491(96)00313-6 9180013

[B65] Babu GJ Warshaw DM Periasamy M Smooth muscle myosin heavy chain isoforms and their role in muscle physiology Microsc Res Tech 2000 50 6 532 540 10.1002/1097-0029(20000915)50:6<532::AID-JEMT10>3.0.CO;2-E 10998642

[B66] Hooper SL Thuma JB Invertebrate muscles: muscle specific genes and proteins Physiol Rev 2005 85 3 1001 1060 10.1152/physrev.00019.2004 15987801

[B67] Eddinger TJ Meer DP Myosin II isoforms in smooth muscle: heterogeneity and function Am J Physiol Cell Physiol 2007 293 2 C493 508 10.1152/ajpcell.00131.2007 17475667

[B68] Walker A Su H Conti MA Harb N Adelstein RS Sato N Non-muscle myosin II regulates survival threshold of pluripotent stem cells Nat Commun 2010 1 71 10.1038/ncomms1074 20842192 PMC3430968

[B69] De Martino C Capanna E Nicotra MR Natali PG Immunochemical localization of contractile proteins in mammalian meiotic chromosomes Cell Tissue Res 1980 213 1 159 178 7006828 10.1007/BF00236928

[B70] De Lange WJ Halabi CM Beyer AM Sigmund CD Germ line activation of the Tie2 and SMMHC promoters causes noncell-specific deletion of floxed alleles Physiol Genomics 2008 35 1 1 4 10.1152/physiolgenomics.90284.2008 18612081 PMC2574738

[B71] Bosgraaf L Van Haastert PJ The regulation of myosin II in Dictyostelium Eur J Cell Biol 2006 85 969 979 10.1016/j.ejcb.2006.04.004 16814425

[B72] Sun SX Walcott S Wolgemuth CW Cytoskeletal cross-linking and bundling in motor-independent contraction Curr Biol 2010 20 15 R649 54 10.1016/j.cub.2010.07.004 20692617 PMC3633214

[B73] Nielsen C Animal evolution : interrelationships of the living phylums 2001 2 New York: Oxford University Press

[B74] Nyitray L Jancso A Ochiai Y Graf L Szent-Györgyi G Scallop striated and smooth muscle myosin heavy-chain isoforms are produced by alternative RNA splicing from a single gene Proc Natl Acad Sci USA 1994 91 12686 12690 10.1073/pnas.91.26.12686 7809102 PMC45504

[B75] Wheatley S Kulkarni S Karess R Drosophila nonmuscle myosin II is required for rapid cytoplasmic transport during oogenesis and for axial nuclear migration in early embryos Development 1995 121 6 1937 1946 7601006 10.1242/dev.121.6.1937

[B76] Edwards KA Kiehart DP Drosophila nonmuscle myosin II has multiple essential roles in imaginal disc and egg chamber morphogenesis Development 1996 22 5 1499 1511 8625837 10.1242/dev.122.5.1499

[B77] DePina AS Langford GM Vesicle transport: the role of actin filaments and myosin motors Microsc Res Tech 1999 47 2 93 106 10.1002/(SICI)1097-0029(19991015)47:2<93::AID-JEMT2>3.0.CO;2-P 10523788

[B78] Brown ME Bridgman PC Myosin functions in nervous and sensory systems J Neurobiol 2003 58 1 118 30 14598375 10.1002/neu.10285

[B79] Seabrooke S Qiu X Stewart BA Nonmuscle Myosin II helps regulate synaptic vesicle mobility at the Drosophila neuromuscular junction BMC Neurosci 2010 11 37 10.1186/1471-2202-11-37 20233422 PMC2853426

[B80] Seabrooke S Stewart BA Synaptic transmission and plasticity are modulated by Nonmuscle Myosin II at the neuromuscular junction of Drosophila J Neurophysiol 2011 105 5 1966 1976 10.1152/jn.00718.2010 21325687

[B81] Force A Lynch M Pickett FB Amores A Yan YL Postletwait J Preservation of duplicate genes by complementary, degenerative mutations Genetics 1999 151 4 1531 1545 10101175 10.1093/genetics/151.4.1531 PMC1460548

[B82] Schuchert P Reber-Müller S Schmid V Life stage specific expression of a myosin heavy chain in the hydrozoan Podocoryne carnea Differentiation 1993 54 1 11 18 10.1111/j.1432-0436.1993.tb00654.x 8104835

[B83] Goodson HV Molecular evolution of the myosin superfamily: application of phylogenetic techniques to cell biological questions Soc Gen Physiol Ser 1994 49 141 145 7939893

