# Supplementary material for: Independent specialisation of myosin II paralogues in muscle vs. non-muscle functions during early animal evolution: a ctenophore perspective
Source: BMC Evol Biol. 2012 Jul 2;12:107. doi: 10.1186/1471-2148-12-107 (PMC3502136; doi:10.1186/1471-2148-12-107)
Supplement: Additional file 1 — Alignment of myosin amino-acid sequences. This file contains the initial alignment (before site pruning by Gblocks) in graphic view. Note that for myosins of classes other than class II (outgroups), only the sequences of the motor domain have been included (the C-terminal part of these proteins is not alignable with the C-terminal part of myosins II). For Saccaromyces cerevisiae MHCII, the portion of the protein sequence downstream from position 1300 has been likewise omitted for the same reason. [file 1471-2148-12-107-S1.pdf]

```
                                        10        20        30        40        50        60        70        80        90       100
                                ....|....|....|....|....|....|....|....|....|....|....|....|....|....|....|....|....|....|....|....|
Mmu_MyoX_gi130507685refNP_0623  ------------------------------------------------------------------------MDS-----------FFPEGARVWL----
Mmu_VIIa_gi115511010refNP_0326  ------------------------------------------------------------------------------------MVILQKGDYVWMDLKS
Mmu_VIIb_gi261823963refNP_1157  ------------------------------------------------------------------------MSV------------FRLGDHVWLDP-P
Ddi_0301_gi166240253refXP_0017  --------------------------------------------------------------------MMIDNNC--------------GKEKVWV---P
Ddi_1951_gi66823681refXP_64519  ------------------------------------------------------MTTSTIENGASSPIIVSSSTP----------KLYQEGAGVWI---P
Sce_9711_gi6324902refNP_014971  -------------------------------------------------------------------------------------MSFEVGTRCWY---P
Sce_9373_gi6319290refNP_009373  -------------------------------------------------------------------------------------MSFEVGTKCWY---P
Mmu_MyoVc_gi124486759refNP_001  ---------------------------------------------------------------------MAVAEL------------YAQYNRVWI---P
Mmu_MyoVa_gi115511052refNP_034  ----------------------------------------------------------------------MAASE-----------LYTKFARVWI---P
Mmu_MyoVb_gi46399202refNP_9638  ----------------------------------------------------------------------MSYSE-----------LYTRYTRVWI---P
Ddimyo                          ---------------------------------------------MNPIHDRTSDYHKYLKVKQGDSDLFKLTVS--------------DKRYIWYNPDP
Sce_1888_gi6321812refNP_011888  --------------------------------------------------------------------MTGGQSC-------------SSNMIVWI---P
Sar_1270_SARC_01270T0Sphaerofo  ----------------------------------------------------MSDSLKHLRDTSEAKGGFADSAD---------QSEWTAQKWIWV---K
Mbr_4119_nullMonosigabrevicoll  -----------------------------------------------------MSVLQHLR-----VQRSSDDVK-----------SWAALKRVWV---P
Sro_4749_PTSG_04749T0Salpingoe  ---------------------------------------------------MTDQVLKYLRIPASSRQGYEDSSA------------WAAKRLAWI---P
PpiMHCa                         ---------------------------------------------------MSDAALKYL---VAERSLALNAIS---------QAEWAEKKLVWV---P
Aqu_8281_gi340368578refXP_0033  ---------------------------------------------------MATEDLKYLS--VD-RGKMYDAIA---------QAEWAKKKLVWV---P
Cow_3939_CAOG_03939T0Capsaspor  ------------------------------------------------MSHKEEDAMAYLS--VGKKNDVADAAV---------QAEWAAKKWVWI---P
TaMHCa                          ----------------------------------------------------MKSSAKELKHLVVEKRAFIDPAV---------QAQWMAKKYVWI---P
Asumyo                          ------------------------------------------------MANNATSEISYLQ--VQ-KGIVADPAL---------QAEWAKKQLVWV---P
Hma_21673571_gi221130996refXP_  ----------------------------MGLNFLNDCLI-------------------------------------------------------------
Nve_JGI5820_jgiNemve1118073jgi  ----------------------------------------------------MENEMKYLV--VD-RAAISDPVA---------LAEWSSKRLVWI---P
Mmu_Myo14_gi29336026refNP_0822  ------------------------------------------MAAVTMSVSGRKVASRPGPVPEAAQSFLYAPRTPNVGGPGGPQVEWTARRMVWV---P
MmsmMHC11                       ----------------------------------------------MAQKGQLSDDEKFL---FVDKNFMNSPMA---------QADWVAKKLVWV---P
Mmu_Myo9_gi114326446refNP_0718  --------------------------------------------------MAQQAADKYL---YVDKNFINNPLA---------QADWAAKKLVWV---P
MmuMHC10                        ----------------------------------------------MAQRTGLEDPERYL---FVDRAVIYNPAT---------QADWTAKKLVWI---P
Lpenm                           --------------------------------------------MAAPEDQFAGMSAQELKYLTVDRNILTDPSA---------QSQWAARRMVWV---P
LgnmMHCII1                      ----------------------------------------------MAEDHLVGLSAQELRYLTVDRSLVNDPSV---------QAEWAGRKLIWV---P
Aaesmnm                         --------------------------------------------MADDLRDRNDPELKYL---SVERNSFNDPAT---------QAEWTQKRLVWV---P
Dme_zipper_gi62471805refNP_001  MKSAWLALKSKSALHPKSEYPVSNIHYPKAANYRQTRNYLEIAAKMSEEVDRNDPELKYL---SVERNQFNDPAT---------QAEWTQKRLVWV---P
PpiMHCb1                        ----------------------------------------------------------------------------------------------------
PpiMHCb2                        ----------------------------------------------------------------------------------------------------
Mmu_Myo15_gi261245016refNP_001  -----------------------------------------------MDLSEFGDATAFLR--RNKADPLFQPPA------------FDGKKKCWV---P
Mmu_Myo7B_gi145864471refNP_001  ----------------------------------------------MMDMSELGESACYLR--QGYQEMMKVHTV-----------PWDGKKRVWV---P
MmcMHC7                         --------------------------------------------MADAEMAAFGAAAPFLR--KSEKERLEAQTR-----------PFDLKKDVFV---P
Mmu_Myo6_gi6754774refNP_034986  --------------------------------------------MTDAQMADFGAAAQYLR--KSEKERLEAQTR-----------PFDIRTECFV---P
Mmu_Myo13_gi124486959refNP_001  -------------------------------------------MSSDAEMAIFGEAAPYLR--KPEKERIEAQNR-----------PFDSKKACFA---V
Mmu_Myo3_gi153792649refNP_0010  -------------------------------------------MSSDTEMEVFGIAAPFLR--KSEKERIEAQNQ-----------PFDAKTYCFV---V
MmskMHC8                        -----------------------------------------MSAGSDAEMAIFGEAAPYLR--KSEKERIEAQNK-----------PFDAKTSVFV---A
Mmu_Myo2_gi205830428refNP_0010  -------------------------------------------MSSDAEMAVFGEAAPYLR--KSEKERIEAQNR-----------PFDAKTSVFV---A
Mmu_Myo4_gi67189167refNP_03498  -------------------------------------------MSSDAEMAVFGEAAPYLR--KSEKERIEAQNK-----------PFDAKSSVFV---V
Mmu_Myo1_gi82524274refNP_10960  -------------------------------------------MSSDAEMAVFGEAAPYLR--KSEKERIEAQNK-----------PFDAKSSVFV---V
Hma_21579261_gi221113859refXP_  ----------------------------------------------MSHDPMLDDPLRYLR--VDPAVALRDQSK-----------SFDAKKWVWV---P
Asumyo4                         ------------------------------------------------MDYENDPGWQYLR--QSREQMIADQSK-----------PYDSKKDCWI---P
AqMHCIIb                        -------------------------------------------------MSGTEDPSVYLR--PTASRTVYDQTK-----------AFDSKKWVWL---P
Aaesmnmmy                       --------------------------------------------MPKPVVQVGGDPDPSEWLFISEEMKRIDQSK-----------PYDAKKACWV---P
DmeMHCC                         -------------------------------------------MPKPVANQEDEDPTPYLF--VSLEQRRIDQSK-----------PYDSKKSCWI---P
Lpemyosin                       -----------------------------------------------MTMDFSDPDMEFLC--LTRQKLMEATSI-----------PFDGKKNCWV---P
LgMHCII3                        ---------------------------------------------MAGSYDPNDPDMKYLA--VDRKKLMEEQTK-----------AFDGKKACWV---P
TaMHCb                          ----------------------------------------------------MDDAEGFLR------QKSQAVTE-----------SIDSKKWVWI---E
Nve_SB1                         -----------------------------------------------------------------DKSQIMAQAK-----------EFESKKWVWI---P

                                       110       120       130       140       150       160       170       180       190       200
                                ....|....|....|....|....|....|....|....|....|....|....|....|....|....|....|....|....|....|....|....|
Mmu_MyoX_gi130507685refNP_0623  -RENGQHFPSTV--NSC-AEGVVVFQ---------T--DYGQVF-TY-----KQSTITNQKVTAMHPLHEEGVDDMASLAELHGGSIMYNLFQRY-KRNQ
Mmu_VIIa_gi115511010refNP_0326  GQEFDVPIGAVV--KLC-DSGQIQVV---------D--DEDNEH-WI-----SPQN--ATHIKPMHPTSVHGVEDMIRLGDLNEAGILRNLLIRY-RDHL
Mmu_VIIb_gi261823963refNP_1157  SSSKTGVAIGGI--VKETKLGKTLIE---------D--DEGKEH-WV-----HAED--LSTLRPMHPNSAQGVDDMIRLGDLNEAGVVHNLLIRY-QQHK
Ddi_0301_gi166240253refXP_0017  -NPEKGWINGDL--IKEIPGEGWLVR---------D--ENGKEI-KI-----EKDE-----LRMQNPVIQEGIDDMTSLSHLHEAAVIHNLIKRY-EINS
Ddi_1951_gi66823681refXP_64519  -DQELGWIGADVIEHSETSADQVLVR---------T--EDDREV-KI-----PLSK-----VFQKNPDILEGVDDLSFLSHLHEPAILHNLHHRY-NLNQ
Sce_9711_gi6324902refNP_014971  -HKELGWIGAEVI-KNEFNDGKYHLE---------LQLEDDEIV-SVDTKDLNNDK-DQSLPLLRNPPILEATEDLTSLSYLNEPAVLHAIKQRY-SQLN
Sce_9373_gi6319290refNP_009373  -HKEQGWIGGEVT-KNDFFEGTFHLE---------LKLEDGETV-SIETNSFENDDDHPTLPVLRNPPILESTDDLTTLSYLNEPAVLHAIKKRY-MNGQ
Mmu_MyoVc_gi124486759refNP_001  -DPEEVWKSAEI--AKDYRAGDRVLR---------LLLEDGMEL-EY-----PVDP--GSLPPLRNPDILVGENDLTALSYLHEPAVLHNLRIRFAESKL
Mmu_MyoVa_gi115511052refNP_034  -DPEEVWKSAEL--LKDYKPGDKVLL---------LHLEEGKDL-EY-----RLDPKTGELPHLRNPDILVGENDLTALSYLHEPAVLHNLRVRFIDSKL
Mmu_MyoVb_gi46399202refNP_9638  -DPDEVWRSAEL--TKDYKEGDKSLQ---------LRLEDDTIL-EY-----PVDVQNNQVPFLRNPDILVGENDLTALSHLHEPAVLHNLKVRFLESNH
Ddimyo                          -KERDSYECGEI--VSE-TSDSFTFK---------T--VDGQDR-QV-----KKDD-----ANQRNPIKFDGVEDMSELSYLNEPAVFHNLRVRY-NQDL
Sce_1888_gi6321812refNP_011888  -DEKEVFVKGELM-STDINKNKFTGQEEQIGIVHPLDSTEVSNLVQV-----RISD-----VFPVNPSTFDKVENMSELTHLNEPSVLYNLEKRY-DCDL
Sar_1270_SARC_01270T0Sphaerofo  -DEADTFIAGNI--TED-KGETYSVR---------L--TNGKVV-EI-----NKND-----SYKMNPPKFEKVEDMAELAYLNEPAVLDNLTKRY-QSNL
Mbr_4119_nullMonosigabrevicoll  -DETEGYVAAEI--RSD-SGDSITCE---------RV-SDHQTV-TI-----PKDD-----TQQMNPPKFNKVEDMAELTHLNEASVLHNLRDRY-MADL
Sro_4749_PTSG_04749T0Salpingoe  -DEKEGFVLTAI--KSD-EGEEVTCQ---------R--EDTREMVTV-----SADD-----VQKANPPKFNKVEDMAELTVLNEASVLFNLRDRY-HADL
PpiMHCa                         -HTEHGFVEGRK--VED-NGDEITVL---------F--ADGSTA-CI-----SADD-----VQKMNPPKFEKVEDMADLSVLNEASVLHNLRDRF-HSDL
Aqu_8281_gi340368578refXP_0033  -HPEAGFVPGSI--KKE-EGDSVTVE---------M--TDGQKK-TV-----SKDD-----IQKMNPPKFEKVEDMAELSFLNEASVLHNLTDRY-YSGL
Cow_3939_CAOG_03939T0Capsaspor  -DDNEGFVAASL--KSE-TDKDATVE---------V--AGGKTL-TV-----KRDD-----VHKANPPKFDKTEDMASLSHLNEASVLQNLKARY-FSNM
TaMHCa                          -DDNHGFTAASI--KSE-RGDEATVQ---------L--NDGRTT-TI-----NRED-----IQKMNPPKFDKVEDMADLTYLNEASVLHNLRDRY-FSGL
Asumyo                          -HETEGFVAGLL--KED-RGDDVVVE---------IA-ETGKKL-TL-----SKDD-----VQKMNPPKFDKVEDMAELTCLNEASVLHNLKERY-FSSL
Hma_21673571_gi221130996refXP_  ---------LSI--KPD-LAKKVNYG---------V--VTSNFV-----------------ARKARKVLFDEVDRIAIKSILNEASVLHNIKDRY-FSDL
Nve_JGI5820_jgiNemve1118073jgi  -DEKQGFVGASI--KDE-SKDEYVVE---------V--EGGKKK-KV-----HKDD-----IQRMNPPKFEKVEDMADLTCLNEASVLHNLKDRY-YSGL
Mmu_Myo14_gi29336026refNP_0822  -SELHGFEAAAL--RDE-GEEEAEVE---------LA-ESGRRL-RL-----PRDQ-----IQRMNPPKFSKAEDMAELTCLNEASVLHNLRERY-YSGL
MmsmMHC11                       -SEKQGFEAASI--KEE-KGDEVVVE---------LV-ENGKKV-TV-----GKDD-----IQKMNPPKFSKVEDMAELTCLNEASVLHNLRERY-FSGL
Mmu_Myo9_gi114326446refNP_0718  -SSKNGFEPASL--KEE-VGEEAIVE---------LV-ENGKKV-KV-----NKDD-----IQKMNPPKFSKVEDMAELTCLNEASVLHNLKERY-YSGL
MmuMHC10                        -SERHGFEAASI--KEE-RGDEVMVE---------LA-ENGKKA-MV-----NKDD-----IQKMNPPKFSKVEDMAELTCLNEASVLHNLKDRY-YSGL
Lpenm                           -SETHGFIGASV--KEE-KGDEVVVD---------VE-DTGKRT-TF-----HRDD-----IQKMNPPKFNKVEDMAELTCLNEASVLHNLKDRY-YSGL
LgnmMHCII1                      -DEQNGFVMASI--KGE-RGDQLEVI---------VD-ETGQKR-TV-----HRDD-----VQKMNPPKFSKVEDMAELTCLNEASVLHNLKDRY-YSGL
Aaesmnm                         -HETQGFVAASI--KGE-RGDEVEVE---------LA-ETGKRV-LV-----LKDD-----IQKMNPPKFDKVEDMAELTCLNEASVLHNIKDRY-YSGL
Dme_zipper_gi62471805refNP_001  -HENQGFVAASI--KRE-HGDEVEVE---------LA-ETGKRV-MI-----LRDD-----IQKMNPPKFDKVEDMAELTCLNEASVLHNIKDRY-YSGL
PpiMHCb1                        ----------------------------------------------------------------------------------------------------
PpiMHCb2                        ----------------------------------------------------------------------------------------------------
Mmu_Myo15_gi261245016refNP_001  -DGKNAYIEAEV--KESGDDGQVIVE---------T--RDGEIM-RI-----KEDK-----LQQMNPEELEMIEDLSMLLYLNEASVLHTLRRRY-DHWM
Mmu_Myo7B_gi145864471refNP_001  -DEQDAYVEAEV--KTEATGGKVTVE---------T--KDQKVL-TV-----RETE-----MQPMNPPRFDLLEDMAMMTHLNEAAVLHNLRQRY-ARWM
MmcMHC7                         -DDKEEFVKAKI--VSR-EGGKVTAE---------T--ENGKTV-TV-----KEDQ-----VMQQNPPKFDKIEDMAMLTFLHEPAVLYNLKERY-ASWM
Mmu_Myo6_gi6754774refNP_034986  -DDKEEYVKAKV--VSR-EGGKVTAE---------T--ENGKTV-TI-----KEDQ-----VMQQNPPKFDKIEDMAMLTFLHEPAVLYNLKERY-AAWM
Mmu_Myo13_gi124486959refNP_001  -DDKEMYVKGMI--QSR-ENDKVIVK---------T--LDDREL-TL-----NSDQ-----VFPMNPPKFDKIEDMAMMTHLHEPAVLYNLKERY-AAWM
Mmu_Myo3_gi153792649refNP_0010  -DSKEEYVKGKI--KSS-QDGKVTVE---------T--EDSRTL-VV-----KPED-----VYAMNPPKFDKIEDMAMLTHLNEPAVLYNLKDRY-TSWM
MmskMHC8                        -EPKESYVKSVI--QSK-DGGKVTVK---------T--ESGATL-TV-----KEDQ-----VFPMNPPKYDKIEDMAMMTHLHEPGVLYNLKERY-AAWM
Mmu_Myo2_gi205830428refNP_0010  -EPKESFVKGTI--QSK-DAGKVTVK---------T--EAGATL-TV-----KEDQ-----IFPMNPPKYDKIEDMAMMTHLHEPAVLYNLKERY-AAWM
Mmu_Myo4_gi67189167refNP_03498  -DAKESYVKATV--QSR-EGGKVTAK---------T--EGGATV-TV-----KDDQ-----VFSMNPPKYDKIEDMAMMTHLHEPAVLYNLKERY-AAWM
Mmu_Myo1_gi82524274refNP_10960  -DAKESFVKATV--QSR-EGGKVTAK---------T--EGGTTV-TV-----KDDQ-----VYPMNPPKYDKIEDMAMMTHLHEPAVLYNLKERY-AAWM
Hma_21579261_gi221113859refXP_  VKEDAGYSAAEV--KGT-NGDMVTVE---------T--EDGKTL-EM-----KKEL-----CEQMNPPKYEKCVDMASLTYLNEASVLYNLKSRY-FEGL
Asumyo4                         -DAEEGFISAQI--KST-KGDMVSVV---------T--SRGNEI-TL-----KSEL-----VQEMNPPKFEKTEDMSNLTFLNDASVLHNLRARY-SSML
AqMHCIIb                        -DEEEGFKSACV--KST-RGDKVLIE---------L--ADGSEK-EV-----DMNI-----TEQMNPPKFEKIEDMAGLTYLNEASVLHNLRQRY-YSSL
Aaesmnmmy                       -DEAEGYLLGEI--KAT-KGELVTVA---------L--PGGETK-DF-----KKDL-----VGQVNPPKYEKCEDMSNLTYLNDASVLHNLRERY-RARL
DmeMHCC                         -DEKEGYLLGEI--KAT-KGDIVSVG---------L--QGGETR-DL-----KKDL-----LQQVNPPKYEKAEDMSNLTYLNDASVLHNLRQRY-YNKL
```

```
Lpemyosin                          -DPDFGFVGAEI--QST-KGDEVTVK---------T--DKTQETRVV-----KKDD-----IGQRNPPKFEMNMDMANLTFLNEASILHNLRSRY-ESGF
LgMHCII3                           -DPQASFLPAEI--VSS-KGDEITVK---------I--TTNNEQRTV-----KKDQ-----IQQMNPPKYEKIEDMANMTFLNEASVLHNLRSRY-TSGL
TaMHCb                             -DKAEGYLSAKI--LEV-NGETLSVE---------V--NTGQRL-TV-----KRDL-----TQQMNPTKYDKAEDMAALTYLNEAGVLNNLKQRY-FSGM
Nve_SB1                            -DPKEGYKAAEV--KST-KGDNFLVE---------T--NDGQEV-EI-----NKND-----TEQMNPPKYEKTEDMSNLTYLNEASVVHNLKQRY-FSGL

                                            210       220       230       240       250       260       270       280       290       300
                                   ....|....|....|....|....|....|....|....|....|....|....|....|....|....|....|....|....|....|....|....|
Mmu_MyoX_gi130507685refNP_0623     IYTYIGSIIASVNPYQPIAGLYERATMEEYSRCH------------LGELPPHIFAIANECYRCLWKRHDNQCVLISGESGAGKTESTKLILKFLSVISQ
Mmu_VIIa_gi115511010refNP_0326     IYTYTGSILVAVNPYQLLS-IYSPEHIRQYTNKK------------IGEMPPHIFAIADNCYFNMKRNNRDQCCIISGESGAGKTESTKLILQFLAAISG
Mmu_VIIb_gi261823963refNP_1157     IYTYTGSILVAVNPFQMLP-LYTLEQVQIYYSRH------------MGELPPHIFAIANSCYFNMKKNKRDQCCIISGESGAGKTETTKLILQFLATVSG
Ddi_0301_gi166240253refXP_0017     IYTYTGSILIAINPYTKLP-IYSKEMIESFCDQP------------VSKLAPHVYSIAESAYREMLNFQKNQSILVSGESGAGKTETTKFLLQYFAAMGE
Ddi_1951_gi66823681refXP_64519     IYTYIGKILIAINPYTSLP-LYGKEMISAYYGKQ------------LGTLAPHVYAVAEDAFKDMRYDGTSQSILVSGESGAGKTETTKFLLQYFAAMGN
Sce_9711_gi6324902refNP_014971     IYTYSGIVLIATNPFDRVDQLYTQDMIQAYAGKR------------RGELEPHLFAIAEEAYRLMKNDKQNQTIVVSGESGAGKTVSAKYIMRYFASVEE
Sce_9373_gi6319290refNP_009373     IYTYSGIVLIAANPFDKVDHLYSREMIQNYSSKR------------KDELEPHLFAIAEEAYRFMVHEKANQTVVVSGESGAGKTVSAKYIMRYFASVQE
Mmu_MyoVc_gi124486759refNP_001     IYTYSGIILVAMNPYKQLP-IYGDAIIHAYSGQN------------MGDMDPHIFAVAEEAYKQMARNNRNQSIIVSGESGAGKTVSARYAMRYFATVSK
Mmu_MyoVa_gi115511052refNP_034     IYTYCGIVLVAINPYEQLP-IYGEDIINAYSGQN------------MGDMDPHIFAVAEEAYKQMARDERNQSIIVSGESGAGKTVSAKYAMRYFATVSG
Mmu_MyoVb_gi46399202refNP_9638     IYTYCGIVLVAINPYEQLP-IYGQDVIYAYSGQN------------MGDMDPHIFAVAEEAYKQMARDEKNQSIIVSGESGAGKTVSAKYAMRYFATVGG
Ddimyo                             IYTYSGLFLVAVNPFKRIP-IYTQEMVDIFKGRR------------RNEVAPHIFAISDVAYRSMLDDRQNQSLLITGESGAGKTENTKKVIQYLASVAG
Sce_1888_gi6321812refNP_011888     IYTYSGLFLVAINPYHNLN-LYSEDHINLYHNKHNRLSKSRLDENSHEKLPPHIFAIAEEAYENLLSEGKDQSILVTGESGAGKTENTKKILQYLASITS
Sar_1270_SARC_01270T0Sphaerofo     IYTYSGLFCVVVNPYKRLP-IYTPDVVDMYQGKR------------RQEMPPHVYNIADSAYRNMLNDRENQSILITGESGAGKTENTKKVIQYIAATAA
Mbr_4119_nullMonosigabrevicoll     IYTYSGLFCVVINPYKQLP-IYTEEAVALYKGKK------------RENMPPHVFAIADTAYRSMLQDRDDQSILCTGESGAGKTENTKKVIQYLASVAA
Sro_4749_PTSG_04749T0Salpingoe     IYTYSGLFCVVVNPYKNIP-IYNDDAIKLYKGQK------------RDAVPPHVFAIADLSYRSMLQDREDQSILCTGESGAGKTENTKKVIQYLASVAA
PpiMHCa                            IYTYSGLFCVVVNPYKRLP-IYTERVIDMYKGKK------------RYEVPPHIFAITDNAYRAMLQDRENQTILCTGESGAGKTENTKKVIQYLASITS
Aqu_8281_gi340368578refXP_0033     IYTYSGLFCVVVNPYKMLP-IYTDRVVDLYRGKK------------RHEMPPHVYAVTDQSYRNMLLDREDQSILCTGESGAGKTENTKKVIQYLAVIAS
Cow_3939_CAOG_03939T0Capsaspor     IYTYSGLFCVVINPYKKLP-IYSDKVVQMYKGRR------------RQELPPHVYALTDSAYRDMLQERENQSILCTGESGAGKTENTKKVIQYLASIAS
TaMHCa                             IYTYSGLFCVVVNPYKRYP-IYTDEVVELYKSKK------------RHEVPPHIYAITDQAYRCMLLEREDQSILCTGESGAGKTENTKKVIQYLAAVAG
Asumyo                             IYTYSGLFCVVVNPYKKLP-IYSEALIEAFKGKK------------RHEMPPHIFAIADTAYRSMLQEREDQSILCTGESGAGKTENTKKVIQYLAYVAG
Hma_21673571_gi221130996refXP_     IYTYSGLFCVVVNPYKKIP-IYSDNVVNLYRGKK------------RHELPPHVYAITDNAYRSMLQDRDNQSILCTGESGAGKTENTKKVIQYLTAIAG
Nve_JGI5820_jgiNemve1118073jgi     IYTYSGLFCVVVNPYKRLP-IYTDKVIDMYRGKK------------RHEVPPHIYAITDTAYRSMLQDRENQSILCTGESGAGKTENTKKVIQYLAAVAG
Mmu_Myo14_gi29336026refNP_0822     IYTYSGLFCVVINPYKQLP-IYTEAIVEMYRGKK------------RHEVPPHVYAVTEGAYRSMLQDREDQSILCTGESGAGKTENTKKVIQYLAHVAS
MmsmMHC11                          IYTYSGLFCVVVNPYKYLP-IYSEKIVDMYKGKK------------RHEMPPHIYAIADTAYRSMLQDREDQSILCTGESGAGKTENTQKVIQYLAVVAS
Mmu_Myo9_gi114326446refNP_0718     IYTYSGLFCVVINPYKNLP-IYSEEIVEMYKGKK------------RHEMPPHIYAITDTAYRSMMQDREDQSILCTGESGAGKTENTKKVIQYLAHVAS
MmuMHC10                           IYTYSGLFCVVINPYKNLP-IYSENIIEMYRGKK------------RHEMPPHIYAISESAYRCMLQDREDQSILCTGESGAGKTENTKKVIQYLAHVAS
Lpenm                              IYTYSGLFCVVVNPYKRLP-IYQDKVIELYRGKK------------RHEVPPHVFAITDTAYRSMLQDREDQSILCTGESGAGKTENTKKVIQYLAYVAA
LgnmMHCII1                         IYTYSGLFCVVINPYKRLP-IYQEKVIELYKGKK------------RHEVPPHVYAIADTAYRSMLQDREDQSILCTGESGAGKTENTKKVIQYLAYVAA
Aaesmnm                            IYTYSGLFCVVVNPYKKLP-IYTEKIMEKYKGIK------------RHEVPPHVFAITDTAYRSMLQDREDQSILCTGESGAGKTENTKKVIQYLAYVAA
Dme_zipper_gi62471805refNP_001     IYTYSGLFCVVVNPYKKLP-IYTEKIMERYKGIK------------RHEVPPHVFAITDSAYRNMLGDREDQSILCTGESGAGKTENTKKVIQFLAYVAA
PpiMHCb1                           -------------PLQGFP-IYTDVVINAYRGKK------------KNEMPPHVFSIADNAYSEMLQNRENQSILITGESGAGKTENTKKVIQYFASIGA
PpiMHCb2                           ----------------------------------------------------------------------------------------------------
Mmu_Myo15_gi261245016refNP_001     IYTYSGIFCVAINPYKWLP-VYQKEVMAAYKRKR------------RSEVPPHIFAVANRAFQDMLRNGENQSIVFTGESGSGKTVNTKLIIQYFATMAA
Mmu_Myo7B_gi145864471refNP_001     IYTYSGLFCVTINPYKWLP-VYTAAVVAAYKGKR------------RSEAPPHIYAVADNAYNDMLRNRENQSMLITGESGAGKTVNTKRVIQYFAIVAA
MmcMHC7                            IYTYSGLFCVTVNPYKWLP-VYNAEVVAAYRGKK------------RSEAPPHIFSISDNAYQYMLTDRENQSILITGESGAGKTVNTKRVIQYFAVIAA
Mmu_Myo6_gi6754774refNP_034986     IYTYSGLFCVTVNPYKWLP-VYNAEVVAAYRGKK------------RSEAPPHIFSISDNAYQYMLTDRENQSILITGESGAGKTVNTKRVIQYFASIAA
Mmu_Myo13_gi124486959refNP_001     IYTYSGLFCVTVNPYKWLP-VYNPEVVAAYRGKK------------RQEAPPHIFSISDNAYQFMLTDRDNQSILITGESGAGKTVNTKRVIQYFATIAV
Mmu_Myo3_gi153792649refNP_0010     IYTYSGLFCVTVNPYKWLP-VYNPEVVDGYRGKK------------RQEAPPHIFSISDNAYQFMLTDRENQSILITGESGAGKTVNTKRVIQYFATIAA
MmskMHC8                           IYTYSGLFCVTVNPYKWLP-VYNPEVVAAYRGKK------------RQEAPPHIFSISDNAYQFMLTDRENQSILITGESGAGKTVNTKRVIQYFATIAV
Mmu_Myo2_gi205830428refNP_0010     IYTYSGLFCVTVNPYKWLP-VYNPEVVAAYRGKK------------RQEAPPHIFSISDNAYQFMLTDRENQSILITGESGAGKTVNTKRVIQYFATIAV
Mmu_Myo4_gi67189167refNP_03498     IYTYSGLFCVTVNPYKWLP-VYNPEVVAAYRGKK------------RQEAPPHIFSISDNAYQFMLTDRENQSILITGESGAGKTVNTKRVIQYFATIAV
Mmu_Myo1_gi82524274refNP_10960     IYTYSGLFCVTVNPYKWLP-VYNAEVVAAYRGKK------------RQEAPPHIFSISDNAYQFMLTDRENQSILITGESGAGKTVNTKRVIQYFATIAV
Hma_21579261_gi221113859refXP_     IYTYSGLFCIAINPYRRLP-IYTDEVIKIYRGKR------------RVECPPHVFAIVDEAYQDMLTEHENQSMLITGESGAGKTENTKKVIQYIAKVAG
Asumyo4                            IYTYSGLFCVVINPYKRLP-IYTDSVASMFMGKR------------RTEMPPHLFAVSDEAYRNMLQDHENQSMLITGESGAGKTENTKKVIAYFAVVGA
AqMHCIIb                           IYTYSGLFCVAINPYRRLP-IYTEQVVNMYKGKR------------RTEMPPHIFAIADNAYRDMLQDRENQSILITGESGAGKTENTKKVIQYFAIVAP
Aaesmnmmy                          IYTYSGLFCIVINPYKRWP-LYTLRVAKMYRGKR------------RNEVPPHLFAISDGAYVNMLTNKENQSMLITGESGAGKTENTKKVIAYFATIGA
DmeMHCC                            IYTYSGLFCVAINPYKRYP-VYTNRCAKMYRGKR------------RNEVPPHIFAISDGAYVDMLTNHVNQSMLITGESGAGKTENTKKVIAYFATVGA
Lpemyosin                          IYTYSGLFCIAINPYRRLP-IYTQGLVDKYRGKR------------RAEMPPHLFSIADNAYQYMLQDRENQSMLITGESGAGKTENTKKVIQYFALVAA
LgMHCII3                           IYTYSGLFCIAINPYKRLP-IYTLSVIAKYRGKR------------KMEMPPHLFAIADNAYQSMVQERENQSVLITGESGAGKTENTKKVIMYFAQVAA
TaMHCb                             IYTYSGLFCVAVNPYRRLP-IYTDKVVEMYKGKR------------RAEMPPHIFSITDNAYNEMLQERENQSILITGESGAGKTENTKKVIQYVATVAG
Nve_SB1                            IYTYSGLFCVAINPYRRLP-IYTDKIVFAYRGKR------------KTEAPPHVFCICDNAYQNMLQDRENQSMLITGESGAGKTENTKKVIQYLAHVSG

                                            310       320       330       340       350       360       370       380       390       400
                                   ....|....|....|....|....|....|....|....|....|....|....|....|....|....|....|....|....|....|....|....|
Mmu_MyoX_gi130507685refNP_0623     --------------------QTLDLGLQEKT------------------------S-----SVEQAILQSSPIMEAFGNAKTVYNNNSSRFGKFVQLNIC
Mmu_VIIa_gi115511010refNP_0326     -----------------------------QH------------------------S-----WIEQQVLEATPILEAFGNAKTIRNDNSSRFGKYIDIHFN
Mmu_VIIb_gi261823963refNP_1157     -----------------------------QH------------------------S-----WIEQQVLEANPILEAFGNAKTIRNDNSSRFGKYIDIHFN
Ddi_0301_gi166240253refXP_0017     --------------KGNGVNTSLISEEDIVE------------------------GN----NIETQVIKSTPILEAFGNSKTLRNDNSSRFGKFIEIHFD
Ddi_1951_gi66823681refXP_64519     MIKESTSSSSINGINTSSDGIPVTPPPSPMK------------------------KSPVDKSVEERVLESTPLLEAFGNAKTLRNDNSSRFGKFIEIHFN
Sce_9711_gi6324902refNP_014971     -------------------ENSATVQHQVEM------------------------S-----ETEQKILATNPIMEAFGNAKTTRNDNSSRFGKYLEILFD
Sce_9373_gi6319290refNP_009373     ---------------------SNNREGEVEM------------------------S-----QIESQILATNPIMEAFGNAKTTRNDNSSRFGKYLQILFD
Mmu_MyoVc_gi124486759refNP_001     ---------------------------SSSN------------------------A-----HVEDKVLASNPITEAVGNAKTTRNDNSSRFGKYTEISFD
Mmu_MyoVa_gi115511052refNP_034     ---------------------------SASE------------------------A-----NVEEKVLASNPIMESIGNAKTTRNDNSSRFGKYIEIGFD
Mmu_MyoVb_gi46399202refNP_9638     ---------------------------SASD------------------------T-----NIEEKVLASSPIMEAIGNAKTTRNDNSSRFGKFIEIGFD
Ddimyo                             ------------------------RNQANGS------------------------G-----VLEQQILQANPILEAFGNAKTTRNNNSSRFGKFIEIQFN
Sce_1888_gi6321812refNP_011888     ----------------GSPSNIAPVSGSSIV------------------------E-----SFEMKILQSNPILESFGNAQTVRNNNSSRFGKFIKIEFN
Sar_1270_SARC_01270T0Sphaerofo     -------------------------GPDKGK------------------------G-----QLEEQLLQCNPVLEAFGNAKTIKNDNSSRFGKFIRIEFD
Mbr_4119_nullMonosigabrevicoll     ---AGR---------AKDVLGGTSASGGMGK------------------------G-----ELERQLLQANPILETFGNAATIKNDNSSRFGKFIKIHFD
Sro_4749_PTSG_04749T0Salpingoe     -------TTRRDPDTLAMFASDAAAGSTMGK------------------------G-----ELERQLLQANPILETFGNAGTIKNDNSSRFGKFIRIHFD
PpiMHCa                            ---RGG---------SGYINDTQNVASPKTVKQKQMIKRKSSMSLSVKTGAQVNMG-----DLELQLLQATPILEAFGNAKTVKNDNSSRFGKFIRITFD
Aqu_8281_gi340368578refXP_0033     ---GGS---------AAKPKGPIRRTSTSTTLNIKAGLTGLQ-------------G-----ELEAQLLQANPILEAFGNAKTVKNDNSSRFGKFIRINFD
Cow_3939_CAOG_03939T0Capsaspor     -------------------------GSSKSQ------------------------G-----QLEAQLLQANPILEAFGNAKTIKNDNSSRFGKFIRIEFD
TaMHCa                             --------------------AAKEMKDGNDG------------------------G-----ELEAQLLEANPILEAFGNAKTIRNDNSSRFGKFIRINFD
Asumyo                             ---ATR---------QSKTPSSPAKPRQSIENVI---------------------G-----ELEHQLLQANPILEAFGNSKTVKNDNSSRFGKFIRINFD
Hma_21673571_gi221130996refXP_     ---------------------RHKQDDKSAQ------------------------G-----QLEVQLLQANPILEAFGNAKTVKNDNSSRFGKFIRIMFD
Nve_JGI5820_jgiNemve1118073jgi     ---HSY---------GKVHATPQKTEKGGKSKPTSMTQVSVSNYLVLFVQ-----V-----ELEKQLLAANPILEAFGNAKTIKNDNSSRFGKFIRINFD
Mmu_Myo14_gi29336026refNP_0822     --------------------SPKGRKEPGVP------------------------G-----ELERQLLQANPILEAFGNAKTVKNDNSSRFGKFIRINFD
MmsmMHC11                          --------------------SHKGKKDSSIT------------------------G-----ELEKQLLQANPILEAFGNAKTVKNDNSSRFGKFIRINFD
Mmu_Myo9_gi114326446refNP_0718     -----------------------SHKSKKDQ------------------------G-----ELERQLLQANPILEAFGNAKTVKNDNSSRFGKFIRINFD
MmuMHC10                           --------------------SHKGRKDHNIP------------------------G-----ELERQLLQANPILESFGNAKTVKNDNSSRFGKFIRINFD
Lpenm                              --------------SSRAANNRSSVASFHGS------------------------G-----ELENQLLQANPILEAFGNAKTIKNDNSSRFGKFVRINFD
LgnmMHCII1                         SSRSTR---------SSVSNVSHSTNKELSL------------------------G-----ELENQLLQANPILESFGNAKTIKNDNSSRFGKFIRINFD
Aaesmnm                            SKPKGSVAVSSRWFIGSIFSPKERWTILGLCKILINIIFSKLFFLRLQQIYSTQQG-----ELEQQLLQANPILEAFGNAKTVKNDNSSRFGKFIRINFD
Dme_zipper_gi62471805refNP_001     ---------------SKPKGSGAVPHPAVLI------------------------G-----ELEQQLLQANPILEAFGNAKTVKNDNSSRFGKFIRINFD
PpiMHCb1                           --------------------TPGKGDGEKKM------------------------------SVEDQVVMCNPVLEAYGNAKTVRNDNSSRFGKFIRVHFG
PpiMHCb2                           ----------------------------------------------------------------------------------------------------
Mmu_Myo15_gi261245016refNP_001     -----------------------ISEPKKKL------------------------G-----NLEDQIVKMNPLLEAFGNAKTQKNDNSSRFGKLIRIHFG
Mmu_Myo7B_gi145864471refNP_001     --------------LGDGPGKKAQFLATKTG------------------------G-----TLEDQIIEANPAMEAFGNAKTLRNDNSSRFGKFIRIHFG
MmcMHC7                            ------------------IGDRSKKDQTPGK------------------------G-----TLEDQIIQANPALEAFGNAKTVRNDNSSRFGKFIRIHFG
Mmu_Myo6_gi6754774refNP_034986     -----------------IGDRSKKENPNANK------------------------G-----TLEDQIIQANPALEAFGNAKTVRNDNSSRFGKFIRIHFG
Mmu_Myo13_gi124486959refNP_001     -----------------TGDKKKEQQPGKMQ------------------------G-----TLEDQIIQANPLLEAFGNAKTVRNDNSSRFGKFIRIHFG
Mmu_Myo3_gi153792649refNP_0010     ------------------TGDLAKKKDSKMK------------------------G-----TLEDQIISANPLLEAFGNAKTVRNDNSSRFGKFIRIHFG
MmskMHC8                           ------------------TGDKKKEESGKMQ------------------------G-----TLEDQIISANPLLEAFGNAKTVRNDNSSRFGKFIRIHFG
Mmu_Myo2_gi205830428refNP_0010     ----------------TGDKKKEEATSGKMQ------------------------G-----TLEDQIISANPLLEAFGNAKTVRNDNSSRFGKFIRIHFG
Mmu_Myo4_gi67189167refNP_03498     ----------------TGDKKKEEATSGKMQ------------------------G-----TLEDQIISANPLLEAFGNAKTVRNDNSSRFGKFIRIHFG
Mmu_Myo1_gi82524274refNP_10960     ----------------TGEKKKEEATSGKMQ------------------------G-----TLEDQIISANPLLEAFGNAKTVRNDNSSRFGKFIRIHFG
```

```
Hma_21579261_gi221113859refXP_   -----------------VEKKSKETSTSAIK------------------------G-----TLDEQVVQANPLLEAFGNAKTTRNNNSSRFGKFIRCHFS
Asumyo4                          ---------------SQQEAVGVQQPEGKKK------------------------V-----TLEDQIVQTNPVLEAFGNARTVRNNNSSRFGKFIRIHFS
AqMHCIIb                         -------------------------DKHKTE------------------------Q-----NLEDQVIQANPVLEAFGNAKTTRNDNSSRFGKFIRIHFG
Aaesmnmmy                        ----------------------SKKDTEGK-------------------------P-----SLEDQVVQTNPVLEAYGNAKTVRNDNSSRFGKFIRIHFT
DmeMHCC                          --------------------SKKTDEAAKSK------------------------G-----SLEDQVVQTNPVLEAFGNAKTVRNDNSSRFGKFIRIHFG
Lpemyosin                        ------------SLAGKKDKKEEEKKKDEKK------------------------G-----TLEDQIVQCNPVLEAYGNAETTRNNNSSRFGKFIRIHFG
LgMHCII3                         ---------------MGQKGDEEEEKKDEKK------------------------G-----TLEDQIVQCNPVLEAYGNAKTTRNNNSSRFGKFIRIHFG
TaMHCb                           ----------------------TGETKSEKK------------------------Q-----NLEDQIVQANPLMEAFGNAKTIRNNNSSRFGKFIRVHFG
Nve_SB1                          -----------------GISKADEEHKKQTG------------------------G-----SLEDQVIQTNPILEAYGNAKTIRNNNSSRFGKFIRCHFG

                                          410       420       430       440       450       460       470       480       490       500
                                 ....|....|....|....|....|....|....|....|....|....|....|....|....|....|....|....|....|....|....|....|
Mmu_MyoX_gi130507685refNP_0623   Q-QGNIQGGRIVDYLLEKNRVVRQNPGERNYHIFYALLAG----------------------LDQGEREEFYL--SLPENYHYLNQSGCTEDKTISDQES
Mmu_VIIa_gi115511010refNP_0326   K-RGAIEGAKIEQYLLEKSRVCRQAPDERNYHVFYCMLEG----------------------MNEEEKKKLGL--GQAADYNYLAMGNCITCEGRVDSQE
Mmu_VIIb_gi261823963refNP_1157   S-SGVIEGASIEHFLLEKSRVCRQAPEERNYHIFYCMLMG----------------------MSPEEKQMLSL--GMPSEYHYLTMGSCTSSEGLSDAKD
Ddi_0301_gi166240253refXP_0017   KIKGTIVGAKLETYLLEKSRIVKPPENERGYHIFYQLIKGFNNSCCLKNSSNNNKDEDSSSSSNNNIDDLKSLLKCKASDFNYLISSGCDSIDGVDDSQV
Ddi_1951_gi66823681refXP_64519   E-MGSIIGAKILTYLLEKSRIVRQVYNERNYHIFYQLLSG----------------------ASEELKEKLNL--KTIEEYSYLNKSGCFEIEGVSDEEH
Sce_9711_gi6324902refNP_014971   K-DTSIIGARIRTYLLERSRLVYQPPIERNYHIFYQLMAG----------------------LPAQTKEELHL--TDASDYFYMNQGGDTKINGIDDAKE
Sce_9373_gi6319290refNP_009373   E-NTTIRGSKIRTYLLEKSRLVYQPETERNYHIFYQILEG----------------------LPEPVKQELHL--SSPKDYHYTNQGGQPNIAGIDEARE
Mmu_MyoVc_gi124486759refNP_001   E-RNQIIGANMRTYLLEKSRVVFQSENERNYHIFYQLCAS----------------------AQQSEFKHLKL--GSAEEFNYTRMGGNTVIEGVNDRAD
Mmu_MyoVa_gi115511052refNP_034   K-RYRIIGANMRTYLLEKSRVVFQAEEERNYHIFYQLCAS----------------------AKLPEFKMLRL--GNADSFHYTKQGGSPMIEGVDDAKE
Mmu_MyoVb_gi46399202refNP_9638   K-KYHIIGANMRTYLLEKSRVVFQADDERNYHIFYQLCAA----------------------ASLPEFKELAL--TCAEDFFYTAHGGNTTIEGVNDADD
Ddimyo                           N-AGFISGASIQSYLLEKSRVVFQSETERNYHIFYQLLAG----------------------ATAEEKKALHL--AGPESFNYLNQSGYVDIKGVSDSEE
Sce_1888_gi6321812refNP_011888   E-HGMINGAHIEWYLLEKSRIVHQNSKERNYHIFYQLLSG----------------------LDDSELKNLRLKSRNVKDYKILSNSNQDIIPGINDVEN
Sar_1270_SARC_01270T0Sphaerofo   R-SGLISGGNIETYLLEKSRAVRQQNDERTFHIFYQLLHG----------------------ATPEMRQELLL--DSVDAVPFLSDKASSSVEGVDDVAE
Mbr_4119_nullMonosigabrevicoll   A-AGVIIGATIDTYLLEKSRAIRQNEGERNFHAFYQLLRG----------------------ADTALITDFGL--QDIKSYRFMSN-GDVTLRDIDDKKE
Sro_4749_PTSG_04749T0Salpingoe   A-AGFIIGATIDTYLLEKSRCVRQNESERTFHSFYQLLRG----------------------ADAKYISELLL--EDISKYEFISN-GDMHLHHVDDGKE
PpiMHCa                          V-SGYISGANIETYLLEKARVIRQALSERTFHIFYQMLYG----------------------ATKEMKEKFLL--EDANDYGFISN-GHESIAGQDDAQD
Aqu_8281_gi340368578refXP_0033   S-SGHIAGANIDTYLLEKSRAVRQATDERTFHIFYQILNG----------------------MDPKEKEEYFY--HSFEKYKFLSN-GNLSVAGINDVQE
Cow_3939_CAOG_03939T0Capsaspor   K-TGHISGGNIETYLLEKSRSIRQSETERDFHIFFQLLRG----------------------ASKDQARDLLL--EDVTKYRFLN--GEKTVEGMDDVAE
TaMHCa                           A-AGFIVGANIETYLLEKSRAIRQAPDERNFHIFYQMLLG----------------------ATPEMKDDLLL--EKIDKYNYMSN-QLVVVPGVDDAEE
Asumyo                           M-SGYISGANIEFYLLEKSRTLRQAADERSFHIFYQFLRG----------------------TSPIEKANFLL--EEVDKYRFLNN-GYICLPNVDDATE
Hma_21673571_gi221130996refXP_   N-SGFISGANIESYLLEKGRLVRQAPEERLFHIFYQLLLG----------------------ASPEVKKQFLL--LDPKSYIFMSN-GLVQLPNMDDRAE
Nve_JGI5820_jgiNemve1118073jgi   A-SGFIAGANIETYLLEKSRAIRQNSNERTFHIFYQMMAG----------------------LEAKERGEILL--GKPTDYRFLSN-GNMPVPGIDDAAE
Mmu_Myo14_gi29336026refNP_0822   I-AGYIVGANIETYLLEKSRAIRQAKDECSFHIFYQLLGG----------------------AGEQLKADLLL--EPCSHYRFLTN-GPSSSPG-QEREL
MmsmMHC11                        V-TGYIVGANIETYLLEKSRAIRQARDERTFHIFYYLLAG----------------------AKEKMKSDLLL--ESFNSYTFLSN-GFVPIPAAQDDEM
Mmu_Myo9_gi114326446refNP_0718   V-NGYIVGANIETYLLEKSRAIRQAKEERTFHIFYYLLSG----------------------AGEHLKTDLLL--EPYNKYRFLSN-GHVTIPGQQDKDM
MmuMHC10                         V-TGYIVGANIETYLLEKSRAVRQAKDERTFHIFYQLLSG----------------------AGEHLKSDLLL--EGFNNYRFLSN-GYIPIPGQQDKDN
Lpenm                            M-SGYICGANIETYLLEKSRSVRQAEGERSFHIFYQFLTG----------------------ASTEQKNDFLL--EDAKSYHYMSS-GPMPVNGVDDVAE
LgnmMHCII1                       L-SGYISGANIESYLLEKSRAVRQAESERSFHVFYQFLNG----------------------ATPAQRKEFLL--EDMSNYRYLSY-GNVPVPGVDDSAD
Aaesmnm                          A-SGYISGANIETYLLEKSRAIRQAKDERTFHIFYQLLAG----------------------ASPEQHDKFIL--DDIKTYPFLSN-GSLPVAGVDDHIE
Dme_zipper_gi62471805refNP_001   A-SGFISGANIETYLLEKSRAIRQAKDERTFHIFYQLLAG----------------------ATPEQREKFIL--DDVKSYAFLSN-GSLPVPGVDDYAE
PpiMHCb1                         R-DSKLASADIEYYLLEKARLTYQNPEERNFHIFYQIFKG----------------------LPQDYLTNTLLMDPDCNNYKFLTN-GDVQVPSQDDVEL
PpiMHCb2                         ----------------------------------------------------------------------------------------------------
Mmu_Myo15_gi261245016refNP_001   A-RGTLSFADIQIYFLEKSRVVYQQPGERNYHIFYQILSG-----------------------NQELRNMLLV-STNPSDFHICSC-GVVAVESLDDAKE
Mmu_Myo7B_gi145864471refNP_001   P-TGKLASADIDSYLLEKSRVIFQLPGERGYHVYYQILSG----------------------KKPELQDMLLL-SMNPYDYHFCSQ-GVTTVDNMDDGEE
MmcMHC7                          A-TGKLASADIETYLLEKSRVIFQLKAERDYHIFYQILSN----------------------KKPELLDMLLI-TNNPYDYAFISQ-GETTVASIDDSEE
Mmu_Myo6_gi6754774refNP_034986   A-TGKLASADIETYLLEKSRVIFQLKAERNYHIFYQILSN----------------------KKPELLDMLLV-TNNPYDYAFVSQ-GEVSVASIDDSEE
Mmu_Myo13_gi124486959refNP_001   A-TGKLASADIETYLLEKSRVTFQLSSERSYHIFYQIMSN----------------------KKPELIDLLLI-STNPFDFPFVSQ-GEVTVASIDDSEE
Mmu_Myo3_gi153792649refNP_0010   T-TGKLASADIETYLLEKSRVTFQLKAERSYHIFYQILSN----------------------KKPELIELLLI-TTNPYDYPFISQ-GEILVASIDDAEE
MmskMHC8                         T-TGKLASADIETYLLEKSRVTFQLKAERSYHIFYQITSN----------------------KKPELIEMLLI-TTNPYDYAFVSQ-GEITVPSIDDQEE
Mmu_Myo2_gi205830428refNP_0010   T-TGKLASADIETYLLEKSRVTFQLKAERSYHIFYQITSN----------------------KKPELIEMLLI-TTNPYDYPFVSQ-GEISVASIDDQEE
Mmu_Myo4_gi67189167refNP_03498   A-TGKLASADIETYLLEKSRVTFQLKAERSYHIFYQIMSN----------------------KKPELIEMLLI-TTNPYDFAYVSQ-GEITVPSIDDQEE
Mmu_Myo1_gi82524274refNP_10960   T-TGKLASADIETYLLEKSRVTFQLKAERSYHIFYQIMSN----------------------KKPDLIEMLLI-TTNPYDYAFVSQ-GEITVPSIDDQEE
Hma_21579261_gi221113859refXP_   Q-QGKLAGADIESYLLEKNRVIHQGSAERNYHIFYQLLYG----------------------TDDDYFNKLCIPSRKAADYEFLNK-GVPSVEGIDDRDE
Asumyo4                          K-KGKVASCDIEHYLLEKSRVIRQAPGERCYHIFYQVYSD----------------------FNPTLKKDLLL-DRPLKEYYFCAQ-AELTIDGVNDKEE
AqMHCIIb                         N-QGKISGADIEYYLLEKSRVIYQQSGERNYHIFYQMMAG----------------------APQKLLDDLLL-NRQVKDYAFLAK-GCVSVDDVDDADM
Aaesmnmmy                        A-SGKLAGADIETYLLEKARVISQQTLERSYHIFYQMMSG----------------------SVKGLKDMCLL-SNDIYDYHNVAQ-GKVTIPNVDDGEE
DmeMHCC                          P-TGKLAGADIETYLLEKARVISQQSLERCYHIFYQIMSG----------------------SVPGVKDICLL-TDNIYDYHIVSQ-GKVTVASIDDAEE
Lpemyosin                        T-QGKIAGADIETYLLEKSRVTYQQSAERNYHIFYQLLSP----------------------AFPENIEKILA-VPDPGLYGFINQ-GTLTVDGIDDEEE
LgMHCII3                         T-QGKISGADIEQYLLEKSRVTYCQPAERNYHIFYQLLSN----------------------AIPAYHEKLLV-SPDPALYSFINQ-GCLTVDSIDDTQE
TaMHCb                           L-HGKIAGADVESYLLEKSRVVSQTSEERNYHVFYQILTA----------------------ADDQMKEKYLV-TGKPEDYKFLSE-GVARIDAVDDEEE
Nve_SB1                          P-QGKLAGADIESYLLEKSRVIYQQEIERNYHIFYQILYG----------------------APKDLHDQLLLESNKTADYAYTAK-GCERADGIDDVEE

                                          510       520       530       540       550       560       570       580       590       600
                                 ....|....|....|....|....|....|....|....|....|....|....|....|....|....|....|....|....|....|....|....|
Mmu_MyoX_gi130507685refNP_0623   FRQVITAMEVMQFSKEEVREVLRLLAGILHLGNIEFIT------AGGAQIP----------------FKTALGRSADLLGL-DPTQLTDALTQRSMILRG
Mmu_VIIa_gi115511010refNP_0326   YANIRSAMKVLMFTDTENWEISKLLAAILHMGNLQYEARTFEN-LDACEVL----------------FSPSLATAASLLEV-NPPDLMSCLTSRTLITRG
Mmu_VIIb_gi261823963refNP_1157   YAHVRSAMKILQFSDSENWDISKLLAAILHLGNVGFMAAVFEN-LDSSDVM----------------ETPAFPLAMKLLEV-QHQALRDCLIKHTIPVLG
Ddi_0301_gi166240253refXP_0017   FIKTENALKVMGLSNDELIGIYKILLSILHIGNIEFEK--GKE-EDSSIIKYGNSSFGESFSDDDAGGYNPLEISCKLLGC-SVDSLKSTFCSRKMKAGN
Ddi_1951_gi66823681refXP_64519   FNKTCHAMQVAGITLVEQENVFRILSAILLIGNFEFENIAGSN-DDSCQLI----------------DRDPLEKVSVLLGCAQPDELLNSMLTRKVVTGK
Sce_9711_gi6324902refNP_014971   YKITVDALTLVGITKETQHQIFKILAALLHIGNIEIKK--TRN--DASLSA----------------DEPNLKLACELLGI-DAYNFAKWVTKKQIITRS
Sce_9373_gi6319290refNP_009373   YKITTDALSLVGINHETQLGIFKILAGLLHIGNIEMKM--TRN--DASLSS----------------EEQNLQIACELLGI-DPFNFAKWIVKKQIVTRS
Mmu_MyoVc_gi124486759refNP_001   MVETQKTFTLLGFKKDFQMDVFKILAAILHLGNVQVTT--VGN-ERSSVSE----------------DDSHLKVFCELLGL-ETSKVAQWLCNRKIVTSS
Mmu_MyoVa_gi115511052refNP_034   MAHTRQACTLLGISESYQMGIFRILAGILHLGNVGFAS--RDS-DSCTIPP----------------KHEPLTIFCDLMGV-DYEEMCHWLCHRKLATAT
Mmu_MyoVb_gi46399202refNP_9638   FEKTRQALTLLGVRDSHQISIFKIIASILHLGSVEIQS--ERDGDSCSISP----------------QDEHLSNFCSLLGI-EHSQMEHWLCHRKLVTTS
Ddimyo                           FKITRQAMDIVGFSQEEQMSIFKIIAGILHLGNIKFEK--GA--GEGAVLK----------------DKTALNAASTVFGV-NPSVLEKALMEPRILAGR
Sce_1888_gi6321812refNP_011888   FKELLSALNIIGFSKDQIRWIFQVVAIILLIGNIEFVS--DR--AEQASFK------------------NDVSAICSNLGV-DEKDFQTAILRPRSKAGK
Sar_1270_SARC_01270T0Sphaerofo   FAITTEAMRVVGINDADQTEVWRTLSGIMKLGQMKFTQ--DR--SDQAQMP----------------DDTIAAMVCKLFGI-PLSDFTKAMLKPRIKVGR
Mbr_4119_nullMonosigabrevicoll   FKQTNEAFGIMGMSSDEINDVWRVISAVLLFGNVNVLG--GRRGDDQASIT----------------SDVCSQRLCSLLGI-NHNDFIKSMTRPRVKAGK
Sro_4749_PTSG_04749T0Salpingoe   FADTNEACRIMGMTDEQIMDVWRVVSAVMLIGNIEVSS--QRRGGEQAVIK----------------DDSVCQKVCHLLGI-NVLDFTRSLLKPKVKAGH
PpiMHCa                          FLDTLEALEIMLATPEEIEAILRIISSCLLFGNVKVKQ--ERN-SDQAILA----------------NNTVTQKICKLLGM-PAVDFEKALLKPRIKVGR
Aqu_8281_gi340368578refXP_0033   YDDTVEAMNVMGISEEEKSAVFRVVSAVLLFGNMEFKQ--ERN-SDQALLV----------------DNTVAQQIGKLLGV-PVTDFTKALLKPKIKTGR
Cow_3939_CAOG_03939T0Capsaspor   FKNTTYAMNVFEISEAEQTAMFKIVSGILQLGNMVFQQ--EKR-SDQAILN----------------DDTYAQKACTMLGI-PLGEFTRSLLKPRVKAGR
TaMHCa                           FQNTLNAIAVMGISNEDTNSSLKILSSLLHMGNLKFDE--DKS-SDQATLP----------------DNTVAQKVCFLLGV-SVTDFTRALLKPRVKVGR
Asumyo                           FHNTIRSMKIMGFHDEEITSVLRLVSAVLLFGNMEFFQ--EKK-SDQAILP----------------DDRVAQKLCHLLGL-PLVEFTKAFLRPRIKVG-
Hma_21673571_gi221130996refXP_   FKLTLEAMRDMGITQEELNPIFKVLSACLLFGNLDFKM--ERK-SDQAALP----------------DNTISQQISHLLGI-AVTDFTNALLKPRVKVGR
Nve_JGI5820_jgiNemve1118073jgi   LADSIVSLHYF-FFFIYFSAIYRVVSAVLHFGNLVFKQ--ERN-SDQAILP----------------NNEVAQKICHLLSM-PVTEFTRAILKPRVKVGR
Mmu_Myo14_gi29336026refNP_0822   FQETLESLRVLGLLPEEITAMLRTVSAVLQFGNIVLKK--ERN-TDQATMP----------------DNTAAQKLCRLLGL-GVTDFSRALLTPRIKVGR
MmsmMHC11                        FQETLEAMSIMGFNEEEQLAILKVVSSVLQLGNIVFKK--ERN-TDQASMP----------------DNTAAQKVCHLVGI-NVTDFTRAILTPRIKVGR
Mmu_Myo9_gi114326446refNP_0718   FQETMEAMRIMGIPEDEQMGLLRVISGVLQLGNIAFKK--ERN-TDQASMP----------------DNTAAQKVSHLLGI-NVTDFTRGILTPRIKVGR
MmuMHC10                         FQETMEAMHIMGFSHEEILSMLKVVSSVLQFGNISFKK--ERN-TDQASMP----------------ENTVAQKLCHLLGM-NVMEFTRAILTPRIKVGR
Lpenm                            FKQTHEAMLVMGLSSDDVNGIFRVVSAVLLFGNMVFRQ--ERN-SDQATLP----------------DNTVAQKVCHLLGL-NVTALTQAFLRPKIKVGR
LgnmMHCII1                       FQSLAESMGIMGISTDDQSAILRIVSAVLLLGNMQFKQ--ERS-SDQATLP----------------DNTVAQKACHLLGL-PVTAVTQAFLKPKIKVGR
Aaesmnm                          FQATVKSMHIMGMTEEDFNSIFRIVSAVLLFGSMKFKQ--ERS-SDQATLP----------------DNTVAQKIAHLLGL-SVTDMTKAFLTPRIKVGR
Dme_zipper_gi62471805refNP_001   FQATVKSMNIMGMTSEDFNSIFRIVSAVLLFGSMKFRQ--ERN-NDQATLP----------------DNTVAQKIAHLLGL-SVTDMTRAFLTPRIKVGR
PpiMHCb1                         WKENEEAMVILNFTDEEKESILKLMCGCLLFGNINLKQ--NKR-DEGCEVD----------------NPVYMEKAAALFQI-PAADFMKCLLKPRVKVGT
PpiMHCb2                         ----------------------------------------------------------------------------------------------------
Mmu_Myo15_gi261245016refNP_001   FLATEKAIDVLGFLPDEKFGCYKLVGAIMHFGNLKFKR--NLR-EEQLEAD----------------GTENADKAAFLMGI-HASELLKGLIYPRIKVGN
Mmu_Myo7B_gi145864471refNP_001   LIATDHAMDILGFSVDEKCACYKIVGALLHFGNMKFKQ--KQR-EEQAEAD----------------GTESADKAAYLMGV-SSGDLLKGLLHPRVRVGN
MmcMHC7                          LMATDSAFDVLGFTPEEKNSIYKLTGAIMHFGNMKFKQ--KQR-EEQAEPD----------------GTEEADKSAYLMGL-NSADLLKGLCHPRVKVGN
Mmu_Myo6_gi6754774refNP_034986   LLATDSAFDVLSFTAEEKAGVYKLTGAIMHYGNMKFKQ--KQR-EEQAEPD----------------GTEDADKSAYLMGL-NSADLLKGLCHPRVKVGN
Mmu_Myo13_gi124486959refNP_001   LLATDNAIDILGFSPEEKVGIYKLTGAVMHYGNMKFKQ--KQR-EEQAEPD----------------GTEVADKAGYLMGL-NSAEMLKGLCCPRVKVGN
```

```
Mmu_Myo3_gi153792649refNP_0010  LLATDSAIDILGFTPEEKSGLYKLTGAVMHYGNMKFKQ--KQR-EEQAEPD----------------GTEVADKTAYLMGL-NSSDLLKALCFPRVKVGN
MmskMHC8                        LMATDSAIDILGFSPEEKVSIYKLTGAVMHYGNMKFKQ--KQR-EEQAEPD----------------GTEVADKAAYLQCL-NSADLLKALCYPRVKVGN
Mmu_Myo2_gi205830428refNP_0010  LMATDSAIDILGFTNDEKVSIYKLTGAVMHYGNMKFKQ--KQR-EEQAEPD----------------GTEVADKAAYLQGL-NSADLLKALCYPRVKVGN
Mmu_Myo4_gi67189167refNP_03498  LMATDTAVDILGFSADEKVAIYKLTGAVMHYGNMKFKQ--KQR-EEQAEPD----------------GTEVADKAAYLTSL-NSADLLKALCYPRVKVGN
Mmu_Myo1_gi82524274refNP_10960  LMATDSAIDILGFTSDERVSIYKLTGAVMHYGNMKFKQ--KQR-EEQAEPD----------------GTEVADKAAYLQNL-NSADLLKALCYPRVKVGN
Hma_21579261_gi221113859refXP_  YNATLEAISVLGLSKDEQDSMFKICSAILNFGNIKLKQ--KPR-DEQAEVV----------------DPADGERVGYLLGI-PVGEFHKSLIKPKVKVGS
Asumyo4                         HQLTDEAFDILHFSPEEKLNCYKLVAAIMHMGNMKFKQ--RPR-EEQAEPD----------------GTDEAEKASAMFGV-NHEEFLKALTRPRVKVGT
AqMHCIIb                        FKQTEDAMNVLGFTTEEKTSLFKTVAGILHFGNIEVKQ--RPR-EEWANIP----------------TATVAEKVAHLLGL-NSTEFIKALIKPRIKVGN
Aaesmnmmy                       CRLTDEAFDILGFTQEEKDNIYKITAAVMHMGGMKFKQ--KGR-EEQAEAD----------------GIEEGDRVAKLLGC-VTDDLYKNLLKPRIKVGT
DmeMHCC                         FSLTDQAFDILGFTKQEKEDVYRITAAVMHMGGMKFKQ--RGR-EEQAEQD----------------GEEEGGRVSKLFGC-DTAELYKNLLKPRIKVGN
Lpemyosin                       MGLTDTAFDVLGFTDEEKLSMYKCTGCILHLGEMKWKQ----R-GEQAEAD----------------GTAEAEKVAFLLGV-NAGDLLKCLLKPKIKVGT
LgMHCII3                        MKDTDKAFDVLGFSEDEKLSYLKCTGAIVHFGEMKFKQ----R-GEQAESD----------------GNAEAEKVAFLLGI-NPEDLLKGFLKPKIKVGT
TaMHCb                          WHATCDSMKTLRFTDEERGFLIKVVMAILHFGNVKFKQ--RPR-EEQAECP----------------DTADAEKVAFLLGI-QVADLVRSLLRPRIRVGH
Nve_SB1                         WANTELAADTLGFSAEEKLSMYKICAACLHWGNSKFKQ--RPR-EEQAEVA----------------DPKDLDKTSFLMKL-PGADFVKNIVKPRIKVGR

                                       610       620       630       640       650       660       670       680       690       700
                                ....|....|....|....|....|....|....|....|....|....|....|....|....|....|....|....|....|....|....|....|
Mmu_MyoX_gi130507685refNP_0623  EEILTPLSVQQAVDSRDSLAMALYARCFEWVIKKINSRIKGKD------DFKSIGILDIFGFENFEVNHFEQFNINYANEKLQEYFNKHIFSLEQLEYSR
Mmu_VIIa_gi115511010refNP_0326  ETVSTPLSREQALDVRDAFVKGIYGRLFVWIVEKINAAIYKPPPLEVKNSRRSIGLLDIFGFENFTVNSFEQLCINFANEHLQQFFVRHVFKLEQEEYDL
Mmu_VIIb_gi261823963refNP_1157  EFVSRPVNIAQATDRRDAFVKGIYGRLFQWIVKKINAAIFTPQAQDPQNVRRAIGLLDIFGFENFQNNSFEQLCINFANEHLQQFFVKHVFTMEQEEYLS
Ddi_0301_gi166240253refXP_0017  ESYTINHTVEQASQARDSLSMFLYSRLFDWLVVRINQSIDKIGTEKKDNSFLFIGILDIYGFESFESNSYEQFTINYANEKLQNQFNHQIFKLEQLEYEK
Ddi_1951_gi66823681refXP_64519  ESYISHNTKERAENARDSLSMFLYGMMFDWLVVKINSSMSISTQQK---SKSFIGVLDIYGFESFEVNGFEQFCINYANEKLQQLFNQHVFKEEQQEYIK
Sce_9711_gi6324902refNP_014971  EKIVSNLNYSQALVAKDSVAKFIYSALFDWLVENINTVLCNPAVNDQ--ISSFIGVLDIYGFEHFEKNSFEQFCINYANEKLQQEFNQHVFKLEQEEYVK
Sce_9373_gi6319290refNP_009373  EKIVTNLNYNQALIARDSVAKFIYSTLFDWLVDNINKTLYDPELDQQDHVFSFIGILDIYGFEHFEKNSFEQFCINYANEKLQQEFNQHVFKLEQEEYVK
Mmu_MyoVc_gi124486759refNP_001  ETVVKPMTRPQAINARDALAKKIYAHLFDFIVEQINQALHFSGK-----QHTFIGVLDIYGFETFDVNSFEQFCINYANEKLQQQFNLHVFKLEQEEYMK
Mmu_MyoVa_gi115511052refNP_034  ETYIKPISKLQATNARDALAKHIYAKLFNWIVDHVNQALHSAVK-----QHSFIGVLDIYGFETFEINSFEQFCINYANEKLQQQFNMHVFKLEQEEYMK
Mmu_MyoVb_gi46399202refNP_9638  ETYVKTMSLQQVVNARDALAKHIYAQLFSWIVEHINKALHTSLK-----QHSFIGVLDIYGFETFEINSFEQFCINYANEKLQQQFNSHVFKLEQEEYMK
Ddimyo                          DLVAQHLNVEKSSSSRDALVKALYGRLFLWLVKKINNVLCQER------KAYFIGVLDISGFEIFKVNSFEQLCINYTNEKLQQFFNHHMFKLEQEEYLK
Sce_1888_gi6321812refNP_011888  EWVSQSKNSQQAKFILNALSRNLYERLFGYIVDMINKNLDHGSA-----TLNYIGLLDIAGFEIFENNSFEQLCINYTNEKLQQFFNNHMFVLEQSEYLK
Sar_1270_SARC_01270T0Sphaerofo  DTVTKAQRADQVVSSIEAIAKAIHERTFLWIVKKINAVLDTRRA-----ASSFIGILDIAGFEIFKVNSFEQLCINFTNEKLQQFFNNHMFVVEQEEYKK
Mbr_4119_nullMonosigabrevicoll  DFVQKAQTKEQVEFALEALAKALYERLFLWVVQRINMTLDKNVRD----SRNFIGILDIAGFEIFKNNSFEQLCINYTNEKLQQLFNHRMFKLEQEEYSK
Sro_4749_PTSG_04749T0Salpingoe  EFVHKQQTKEQVDFAKEALAKATYERLFLWIVKRINKTLDRNIRD----ARTFIGILDIAGFEIFKVNSFEQLCINYTNERLQQLFNSRMFQLEQDEYQK
PpiMHCa                         EYVVKAQNQDQVIFSCESIAKAIYEKTFKWIANKINKVLSRNQYQ----GGSFVGILDIAGFEIFRINSFEQLCINYTNEKLQQLFNHTMFILEQEEYRT
Aqu_8281_gi340368578refXP_0033  EFTIRSQNKSQVEYSCQSLAKALYERMFKWIVQRVNKTLDRSIGS----GTSFIGILDIAGFEIFKVNSFEQLCINYTNEKLQQLFNHTMFILEQEEYRT
Cow_3939_CAOG_03939T0Capsaspor  DIVTKAQNKEQVEFAVEAISKALYERLFKHVVARINKALDTKRQ-----SSSFIGILDIAGFEIFKVNSFEQLCINYTNEKLQQLFNHHMFILEQEEYQK
TaMHCa                          DYVVKAQNKDQASFAVQALSKALYKRLFKWLVSRVNRTLDRTKRQ----GASFIGILDIAGFEIFKLNSFEQLCINFTNEKLQQLFNHTMFILEQEEYRK
Asumyo                          --VHKAQNKEQAEFAVEAIAKACYEKMFRWLVARLNKSLDRTRRQ----GASFIGILDIAGFEIFELNSFEQLCINYTNEKLQQLFNNTMFILEQEEYQR
Hma_21673571_gi221130996refXP_  EFTQKAQTKAQCEFAVEALTKAMYERLFKWLVTRINKSLNRSKRE----GASFVGILDIAGFEIFKINSFEQLCINYTNEKLQQLFNHTMFILEQEEYQK
Nve_JGI5820_jgiNemve1118073jgi  DYVQRAVTKDQADSSVEAIAKALYERLFKWVVQKINKSMDRTKRE----GASFIGILDIAGFEIFQFNSFEQMCINYTNEKLQQLFNHTMFILEQEEYRK
Mmu_Myo14_gi29336026refNP_0822  DYVQKAQTKEQADFALEALAKATYERLFRWLVLRLNRALDRSPRQ----GASFLGILDIAGFEIFQLNSFEQLCINYTNEKLQQLFNHTMFVLEQEEYQR
MmsmMHC11                       DVVQKAQTKEQADFAIEALAKATYERLFRWILSRVNKALDKTHRQ----GASFLGILDIAGFEIFEVNSFEQLCINYTNEKLQQLFNHTMFILEQEEYQR
Mmu_Myo9_gi114326446refNP_0718  DYVQKAQTKEQADFAIEALAKATYERMFRWLVLRINKALDKTKRQ----GASFIGILDIAGFEIFDLNSFEQLCINYTNEKLQQLFNHTMFILEQEEYQR
MmuMHC10                        DYVQKAQTKEQADFAVEALAKATYERLFRWLVHRINKALDRTKRQ----GASFIGILDIAGFEIFELNSFEQLCINYTNEKLQQLFNHTMFILEQEEYQR
Lpenm                           DHVTKAQTKEQAEYSVEAISKACYERMFKWLVIRINKSLDRTKRQ----GAS--GILDIAGFEIFKMNSFEQLCINYTNEKLQQLFNHTMFILEQEEYQR
LgnmMHCII1                      DSVTKAQTKEQVEFAVEAISKALYERMFKWIVSRINKSLDKTKRQ----GASFIGILDIAGFEIFKMNSFEQLCINYTNEKLQQLFNHTMFVLEQEEYQK
Aaesmnm                         DFVTKAQTKEQVEFAVEAIAKACYEKMFKWLVNRINRSLDRTKRQ----GASFIGILDMAGFEIFELNSFEQLCINYTNEKLQQLFNHTMFILEQEEYQR
Dme_zipper_gi62471805refNP_001  DFVTKAQTKEQVEFAVEAIAKACYERMFKWLVNRINRSLDRTKRQ----GASFIGILDMAGFEIFELNSFEQLCINYTNEKLQQLFNHTMFILEQEEYQR
PpiMHCb1                        EMVQKSQNSEQVYFATKALCKSIFERVFKWLIGKCNTALDTKKP-----RQNFCGVLDIAGFEIFDFNTFEQLCINLTNERLQQFFNHHMFILEQEEYKK
PpiMHCb2                        ----------------------------------------------------------------------------------------------------
Mmu_Myo15_gi261245016refNP_001  EYVTRSQNLQQVTYAVGALSQSIYERMFQWLVARMNQVLDAKLT-----SHFFVGILDTTGFEILDYNSLEQLCINFTNEKLQQFFNQQLFILEQEEYRK
Mmu_Myo7B_gi145864471refNP_001  EYVTKGQSVEQVVFAVGALAKATYDRLFRWLVSRINQTLDTKL-P----RQFFIGVLDIAGFEIFEFNSFEQLCINFTNEKLQQFFNQHMFVLEQEEYKR
MmcMHC7                         EYVTKGQNVQQVSYAIGALAKSVYEKMFNWMVTRINATLETKQP-----RQYFIGVLDIAGFEIFDFNSFEQLCINFTNEKLQQFFNHHMFVLEQEEYKK
Mmu_Myo6_gi6754774refNP_034986  EYVTKGQSVQQVYYSIGALAKSVYEKMFNWMVTRINATLETKQP-----RQYFIGVLDIAGFEIFDFNSFEQLCINFTNEKLQQFFNHHMFVLEQEEYKK
Mmu_Myo13_gi124486959refNP_001  EYVTKGQNVQQVTNSVGALAKAVYEKMFLWMVTRINQQLDTKQP-----RQYFIGVLDIAGFEIFDFNSLEQLCINFTNEKLQQFFNHHMFVLEQEEYKK
Mmu_Myo3_gi153792649refNP_0010  EYVTKGQTVDQVHHAVNALSKSVYEKLFLWMVTRINQQLDTKLP-----RQHFIGVLDIAGFEIFEYNSLEQLCINFTNEKLQQFFNHHMFVLEQEEYKK
MmskMHC8                        EYVTKGQTVQQVYNAVGALAKAVYEKMFLWMVTRINQQLDTKQP-----RQYFIGVLDIAGFEIFDFNSLEQLCINFTNEKLQQFFNHHMFVLEQEEYKK
Mmu_Myo2_gi205830428refNP_0010  EYVTKGQTVEQVTNAVGALAKAMYEKMFLWMVTRINQQLDTKQP-----RQYFIGVLDIAGFEIFDFNSLEQLCINFTNEKLQQFFNHHMFVLEQEEYKK
Mmu_Myo4_gi67189167refNP_03498  EYVTKGQTVQQVYNSVGALAKSMYEKMFLWMVTRINQQLDTKQP-----RQYFIGVLDIAGFEIFDFNTLEQLCINFTNEKLQQFFNHHMFVLEQEEYKK
Mmu_Myo1_gi82524274refNP_10960  EYVTKGQTVQQVYNSVGALAKAVYEKMFLWMVTRINQQLDTKQP-----RQYFIGVLDIAGFEIFDFNSLEQLCINFTNEKLQQFFNHHMFVLEQEEYKK
Hma_21579261_gi221113859refXP_  EFVNKGQNVNQVLNAVAALSKALFERMFLWIVQRVNKALDTKER-----RVYFIGVLDIAGFEIFEYNSFEQLCINLTNEKLQQFFNHHMFVLEQEEYKR
Asumyo4                         EWVAKGQNIDQVTWAVGAMAKGLYSRVFNWLVKKCNITLDQKGIP----RDYFIGVLDIAGFEIFDFNSFEQLWINFVNEKLQQFFNHHMFVLEQEEYAR
AqMHCIIb                        EYVQQGRTEGQVNYSIGALSKAMYERMFKWLVGRVNKTLDTKNR-----KAYFIGVLDIAGFEIFKVNSFEQLCINFTNEKLQQFFNHHMFVLEQEEYKK
Aaesmnmmy                       EYVTKGQNKDQVYNAVGALCKGIFDRLFKWLVKKCNETLDTKQK-----RAQFIGVLDIAGFEIFDFNGFEQLCINFTNEKLQQFFNHHMFVLEQEEYKK
DmeMHCC                         EFVTQGRNVQQVTNSIGALCKGVFDRLFKWLVKKCNETLDTQQK-----RQHFIGVLDIAGFEIFEYNGFEQLCINFTNEKLQQFFNHHMFVLEQEEYKR
Lpemyosin                       EYVTQGRNKDQVTNSIAALAKSLYDRMFNWLVRRVNQTLDTKAK-----RQFFIGVLDIAGFEIFDFNSFEQLCINYTNERLQQFFNHHMFVLEQEEYKK
LgMHCII3                        EFVTQGRTKDQVVYSVSALAKSLYDRMFKSMVVRVNKTLDTKAK-----RQFFIGVLDIAGFEIFDFNSFEQLCINYTNERLQQFFNHHMFVLEQEEYKK
TaMHCb                          EYVQQGRNYEQVVSSVAALSKSLYDRMFKWLIARINKTLETKFS-----KNYFIGVLDIAGFEIFQVNLFEQLSINYTNEKLQQFFNHHMFILEQEEYKK
Nve_SB1                         EYVNQGRNLQQVNYSIGALTKSLYERMFLWLVDRANQTLMTKDR-----RAFFIGVLDIAGFEIFQFNSFEQLCINVTNEKLQQFFNHHMFILEQEEYKR

                                       710       720       730       740       750       760       770       780       790       800
                                ....|....|....|....|....|....|....|....|....|....|....|....|....|....|....|....|....|....|....|....|
Mmu_MyoX_gi130507685refNP_0623  EGLVWEDIDW-IDNGECLDLI-EKKL---GLLALINEES-HFPQATDSTLLEKLHSQHAN---NHFYVKP-------RVAVNNFGVKHYAGEVQYDVRGI
Mmu_VIIa_gi115511010refNP_0326  ESIDWLHIEF-TDNQEALDMIANRPM---NVISLIDEES-KFPKGTDATMLHKLNSQHKL---NANYVPP------KNSHETQFGINHFAGVVYYESQGF
Mmu_VIIb_gi261823963refNP_1157  ENITWNYIHY-TDNQPILDMLALKPM---SIISLLDEES-RFPQGTDVTMLQKLNSIHAN---NKSFLSP------RSIHDTRFGIAHFAGDVYYQAEGF
Ddi_0301_gi166240253refXP_0017  EKIDWSYIEF-SDNQECIDLIEKKPL---GILSILDEES-QFPKSTPSTLCTKLYNNHSK---SKNFEKP-------RFSQTHFIIDHYAGKVEYDTNLF
Ddi_1951_gi66823681refXP_64519  EKIDWSYIDF-NDNQDTLDLIEKKPI---CILTLLDEET-MFPKATPQTLATKLYSKMTS---HSKFEKP-------RFSSTAFTINHYAGKVTYETDQF
Sce_9711_gi6324902refNP_014971  EEIEWSFIEF-NDNQPCIDLI-ENKL---GILSLLDEES-RLPAGSDESWTQKLYQTLDKSPTNKVFSKP-------RFGQTKFIVSHYALDVAYDVEGF
Sce_9373_gi6319290refNP_009373  EEIEWSFIEF-SDNQPCIDLI-ENKL---GILSLLDEES-RLPSGSDESWASKLYSAFNKPPSNEVFSKP-------RFGQTKFIVSHYAVDVEYEVEGF
Mmu_MyoVc_gi124486759refNP_001  EDIPWTLIDF-YDNQPVIDLI-EAKM---GILELLDEEC-LLPHGTDENWLQKLYNNFVN--KNSLFEKP-------RMSNSSFIIQHFADKVEYQCEGF
Mmu_MyoVa_gi115511052refNP_034  EQIPWTLIDF-YDNQPCINLI-ESKL---GILDLLDEEC-KMPKGTDDTWAQKLYNTHLN--KCALFEKP-------RMSNKAFIIKHFADKVEYQCEGF
Mmu_MyoVb_gi46399202refNP_9638  EQIPWTLIDF-YDNQPCIDLI-EAKL---GILDLLDEEC-KVPKGTDQNWAQKLYERHSN---SQHFQKP-------RMSNTAFIVNHFADKVEYLSDGF
Ddimyo                          EKINWTFIDFGLDSQATIDLI-DGRQ-PPGILALLDEQS-VFPNATDNTLITKLHSHFSK--KNAKYEEP-------RFSKTEFGVTHYAGQVMYEIQDW
Sce_1888_gi6321812refNP_011888  ENIQWDYIDYGKDLQLTIDLI-ESKGPPTGVLPLLDEEA-VLPKSTDESFYSKLISTWDQ--NSSKFKRS--------RLKNGFILKHYAGDVEYTVEGW
Sar_1270_SARC_01270T0Sphaerofo  EGIDWQFINFGLDLQPCIDLI-EKPM---GVLALLDEECHLMPRSSDSTLVEKMDKAHSG--KSDKYRKL---GGKIKRDDVGFNCQHYAGEVEYTCDGW
Mbr_4119_nullMonosigabrevicoll  EEIDWEFIDFGLDLQPTIELI-DGQM---GVLKLVDEEC-FFPKATDKSLAEKVVHNCKS---HEKLVPP------GVRDDFDFAIVHYAGTVQYSAEGW
Sro_4749_PTSG_04749T0Salpingoe  ENIDWEFIDFGLDLQPTIDLI-ERK----GILPLLDDEC-FFPKATDQSFVDKVVKLL-G--DDEKLSKP------ALREDCDFAVVHYAGRVPYQAAGW
PpiMHCa                         EGIEWKFIDFGLDLQPCIDLI-EKPC---GIMALLDEEC-WFPKATDQTYVSKVENECAG---KQKFSKP------DFRSDADFCVLHYAGYVEYKAEKW
Aqu_8281_gi340368578refXP_0033  EGIEWTFIDFGLDLQPCIDLI-EKPM---GILSLLDEEC-WFPKATDKSYVEKLQREHAQ---NPKYSKP------DFRSSSDFTLGHYAGNVHYNCDQW
Cow_3939_CAOG_03939T0Capsaspor  EGIDWTFIDFGLDLQPCIDLL-EKPL---GILSILDEEC-LFPKATDKSFVEKLDSNHDK--KHPKYKKP-----PPVKSNADFIVVHYAGEVGYMAEQW
TaMHCa                          ENIDWKFIDFGLDLQPTIDLI-EKPL---GILALLDEEC-WFPKATDKTFSDKVEKQHKN---HPKYHKP------DFRENADFCLIHYAGKVTYCTNQW
Asumyo                          EGIDWKFIDFGLDLQPTIDLI-EKPM---GILALLDEQC-LFPKATDKSLVEKLLVNHAK---HPKFVIP------EMRAKSDFAVIHYAGRVDYSADQW
Hma_21673571_gi221130996refXP_  EGIDWKFIDFGLDLQPTIDLI-EKPM---GIMALLDEEC-WFPKATDKSLVEKINKAHAK---HPKYMKP------DFRANSDFCIIHYAGRVDYSAAQW
Nve_JGI5820_jgiNemve1118073jgi  EGIDWKFIDFGLDLQPCINLL-EKPM---GIFALCDEEC-WFPKATDKTLVEKLIKEHNK---HEKFQIP------EFRSQAHFSIIHYAGRVDYNCDNW
Mmu_Myo14_gi29336026refNP_0822  EGIPWTFLDFGLDLQPCIDLI-ERPANPPGLLALLDEEC-WFPKATDKSFVEKVAQEQGS---HPKFQRP-----RNLRDQADFSVLHYAGKVDYKASEW
MmsmMHC11                       EGIEWNFIDFGLDLQPSIELI-ERPNNPPGVLALLDEEC-WFPKATDKSFVEKLCSEQGN---HPKFQKP-----KQLKDKTEFSIIHYAGKVDYNASAW
Mmu_Myo9_gi114326446refNP_0718  EGIEWNFIDFGLDLQPCIDLI-EKPAGPPGILALLDEEC-WFPKATDKSFVEKVVQEQGT---HPKFQKP-----KQLKDKADFCIIHYAGKVDYKADEW
MmuMHC10                        EGIEWNFIDFGLDLQPCIDLI-ERPANPPGVLALLDEEC-WFPKATDKTFVEKLVQEQGS---HSKFQKP-----RQLKDKADFCIIHYAGKVDYKADEW
Lpenm                           EGMEWKFIDFGLDLQPTIDLI-EKPM---GILALLDEEC-WFPKATDKTYVDKLLGHHVN---RPKFEKP------DFRADADFSLIHYAGRVDYSAQAW
LgnmMHCII1                      EGIEWKFIDFGLDLQPTIDLL-EKPM---GILALLDEEC-WFPKATDKTFVEKLHKQHTT---HPKFIKK------DFRADCDFSLVHYAGNVPYQAERW
Aaesmnm                         EGIEWKFIDFGLDLQPTIDLI-DKPG---GIMALLDEEC-WFPKATDKTFVEKLATAHTM---HPKFMKT------DFRGVADFAVVHYAGKVDYSATKW
Dme_zipper_gi62471805refNP_001  EGIEWKFIDFGLDLQPTIDLI-DKPG---GIMALLDEEC-WFPKATDKTFVDKLVSAHSM---HPKFMKT------DFRGVADFAIVHYAGRVDYSAAKW
PpiMHCb1                        EGIVWKFIDFGLDLEASIELM-EKPM---GILPMLDEEC-IMPKATDNSYKDKVIQQHNK--KHPKFSVPSMKAIKGLEHPPHFNIHHYAGVVGYNVGDW
PpiMHCb2                        ----------------------------------------------------------------------------------------------------
```

```
Mmu_Myo15_gi261245016refNP_001  EGLDWLSIDYGLDVQACIDFI-EKPM---GIFSILEEEC-MLPKATDQMFKTKLFDHHFG--KSAYFQTP---TSPEKNFEVHFELAHYAGVVPYNISGW
Mmu_Myo7B_gi145864471refNP_001  EGIDWVFIDFGLDLQPCIDLI-EKPL---GILSILEEEC-MFPKASDASFRAKLYDNHSG--KSPNFQQP--RPDKKRKYQAHFEVVHYAGVVPYSIVGW
MmcMHC7                         EGIEWTFIDFGMDLQACIDLI-EKPM---GIMSILEEEC-MFPKATDMTFKAKLYDNHLG--KSNNFQKP---RNVKGKQEAHFSLVHYAGTVDYNILGW
Mmu_Myo6_gi6754774refNP_034986  EGIEWEFIDFGMDLQACIDLI-EKPM---GIMSILEEEC-MFPKASDMTFKAKLYDNHLG--KSNNFQKP---RNVKGKQEAHFSLVHYAGTVDYNIMGW
Mmu_Myo13_gi124486959refNP_001  EGIEWEFIDFGMDLAACIELI-EKPM---GIFSILEEEC-MFPKATDTSFKNKLYDQHLG--KSNNFQKP---KPTKGKAEAHFSLVHYAGTVDYNIAGW
Mmu_Myo3_gi153792649refNP_0010  EGIEWTFIDFGMDLAACIELI-EKPM---GIFSILEEEC-MFPKATDTSFKNKLYDQHLG--KSNNFQKP---KVVKGKAEAHFSLVHYAGTVDYSVSGW
MmskMHC8                        EGIEWTFIDFGMDLAACIELI-EKPL---GIFSILEEEC-MFPKATDTSFKNKLYDQHLG--KSNNFQKP---KPTKGKAEAHFSLVHYAGTVDYNITGW
Mmu_Myo2_gi205830428refNP_0010  EGIEWTFIDFGMDLAACIELI-EKPM---GIFSILEEEC-MFPKATDTSFKNKLYEQHLG--KSANFQKP---KVVKGKAEAHFSLIHYAGTVDYNITGW
Mmu_Myo4_gi67189167refNP_03498  EGIDWEFIDFGMDLAACIELI-EKPM---GIFSILEEEC-MFPKATDTSFKNKLYEQHLG--KSNNFQKP---KPAKGKAEAHFSLVHYAGTVDYNIIGW
Mmu_Myo1_gi82524274refNP_10960  EGIEWEFIDFGMDLAACIELI-EKPM---GIFSILEEEC-MFPKATDTSFKNKLYEQHLG--KSNNFQKP---KPAKGKVEAHFSLVHYAGTVDYNIAGW
Hma_21579261_gi221113859refXP_  EGINWEFIDFGMDLEQTIDLI-EKPM---GIFAILEEEC-IVPKATDATYLAKLHKQHGG--NNPSYEKPTNKKSAGKSGTGDFILHHYAGSVGYSVEGW
Asumyo4                         EGIQWTFIDFGLDLQACIELI-EKPL---GIISILDEEC-IVPKATDLTLAQKLNDQHLG--KHPNFEKP--KPPKGKQGEAHFAMRHYAGTVRYNVMNW
AqMHCIIb                        EGIKWEFIDFGLDLQPCIDLI-EKQM---GVLALLDEEC-LFPKASDKSYVEKLVKNHDG--KSPNFINP---RDKMKKDVPHFMLAHYAGEVDYTVSGW
Aaesmnmmy                       EGINWAFIDFGMDLLACIELI-EKPM---GILSILEEES-MFPKATDQTFAEKLMNNHLG--KSPPFMKP--RPPKPGIPAGHFAIGHYAGVVSYNITGW
DmeMHCC                         EGIDWAFIDFGMDLLACIDLI-EKPM---GILSILEEES-MFPKATDQTFSEKLTNTHLG--KSAPFQKP--KPPKPGQQAAHFAIAHYAGCVSYNITGW
Lpemyosin                       EGIVWEFIDFGLDLQACIELI-EKPM---GILSILEEEC-MFPKASDTSFKNKLYDNHLG--KNPMFGKP--KPPKAGCAEAHFCLHHYAGSVSYSIAGW
LgMHCII3                        EGIEWEFIDFGMDLQACVDLI-EKPL---GILSILEEEC-MFPKASDKSFMEKLYQNHLG--KSPNFGKP---VGKKVKGEAHFELHHYAGTVPYSITAW
TaMHCb                          ENIDWEFIDFGHDLQPCIDLI-EKKL---GILSILDEES-IYPKASDKTFIEKLKKNHDG--KSPKFKLP----KMSSKNKAHFEIEHYAGTVGYTVMGW
Nve_SB1                         EGIHWEFIDFGHDLEPTINLIFGKGT---GIFAILEEEC-IVPKATDQTFLQKLNNTHDG--KSAKFGKP--KISGKKQVNYHFEIHHYAGTVGYNVDNW

                                       810       820       830       840       850       860       870       880       890       900
                                ....|....|....|....|....|....|....|....|....|....|....|....|....|....|....|....|....|....|....|....|
Mmu_MyoX_gi130507685refNP_0623  LEKNRDTFRDDLLNLLRES-RFDFIYDLFEHVSSRNNQD-------------------------------------------------------------
Mmu_VIIa_gi115511010refNP_0326  LEKNRDTLHGDIIQLVHSS-RNKFIKQIFQADV-------------------------------------------------------------------
Mmu_VIIb_gi261823963refNP_1157  LEKNRDVLSTDILILIHSS-KNKFLKEIFNVDSSQTKLGHGTICQVK-----------------------------------------------------
Ddi_0301_gi166240253refXP_0017  LEKNKDFIISEQVSALESS-NWKFLTNLFQILSKKMNGGGGTSGG-------------------------------------------------------
Ddi_1951_gi66823681refXP_64519  LDKNKDFIIPEQISILQRS-NFSFIKVLMSHSDKFTQSPGGHPQGN------------------------------------------------------
Sce_9711_gi6324902refNP_014971  IEKNRDTVSDGHLEVLKAS-TNETLINILEGLEKAAKKLEEAKK--------------------------------------------------------
Sce_9373_gi6319290refNP_009373  IEKNRDSVSLGHLDVFKAT-TNPIFKQILDNRELRSDDAPEEQN--------------------------------------------------------
Mmu_MyoVc_gi124486759refNP_001  LEKNRDTVYDMLVEILRAS-KFHLCAAFFQESPVPSSPFGAMITVK------------------------------------------------------
Mmu_MyoVa_gi115511052refNP_034  LEKNKDTVFEEQIKVLKSS-KFKMLPELFQDDEKAISPTSATSSGRTP----------------------------------------------------
Mmu_MyoVb_gi46399202refNP_9638  LEKNRDTVYEEQINILKAS-KFPLVADLFHDDKDS-APATNTAKNRSS----------------------------------------------------
Ddimyo                          LEKNKDPLQQDLELCFKDS-SDNVVTKLFND---------------------------------------------------------------------
Sce_1888_gi6321812refNP_011888  LSKNKDPLNDNLLSLLSSS-QNDIISKLFQPEGEKSSSAGVEAN--------------------------------------------------------
Sar_1270_SARC_01270T0Sphaerofo  LTKNTDPLNDNITALLSQS-TNPFTASLWADMV-------------------------------------------------------------------
Mbr_4119_nullMonosigabrevicoll  LVKNKDPLNDNATAQLEDS-SIPFIKSLWSDDFST-----------------------------------------------------------------
Sro_4749_PTSG_04749T0Salpingoe  LVKNKDPLNDNVTALLAKS-GNAFVATLWSDMFSG-----------------------------------------------------------------
PpiMHCa                         LLKNMDPLNENIVELLSNS-GDPFIAGLFKDTSTNPGFSSMANS--------------------------------------------------------
Aqu_8281_gi340368578refXP_0033  LMKNMDPLNDNVIQLLVAS-NDWFVATLWKDTAHVVGMGVQAQSEAK-----------------------------------------------------
Cow_3939_CAOG_03939T0Capsaspor  LVKNMDPLNDNVTELLAKS-TEPLVAELWSDGYVAPAQASES----------------------------------------------------------
TaMHCa                          LMKNMDPLNDNVITLLQQS-SDGFVAGLWKDSSNVVGMNATNTD--------------------------------------------------------
Asumyo                          LMKNMDPLNENVVALFQNS-SDPFVVSIWKDAEFAGICASELN---------------------------------------------------------
Hma_21673571_gi221130996refXP_  LTKNMDPLNDNVVALLAAS-SEPFVDALWKDIENVVSMSVTEGMD-------------------------------------------------------
Nve_JGI5820_jgiNemve1118073jgi  LMKNQDPLNDNITSLMQGS-SDTFVVALWKDAEFVSQFANSG----------------------------------------------------------
Mmu_Myo14_gi29336026refNP_0822  LMKNMDPLNDNVAALLHQS-TDRLTAEIWKDVEGIVGLEQVSSLG-------------------------------------------------------
MmsmMHC11                       LTKNMDPLNDNVTSLLNAS-SDKFVADLWKDVDRIVGLDQMAKMTES-----------------------------------------------------
Mmu_Myo9_gi114326446refNP_0718  LMKNMDPLNDNIATLLHQS-SDKFVSELWKDVDRIIGLDQVAGMSET-----------------------------------------------------
MmuMHC10                        LMKNMDPLNDNVATLLHQS-SDRFVAELWKDVDRIVGLDQVTGMTET-----------------------------------------------------
Lpenm                           LMKNMDPLNENVVALLQNS-SDPFIQLIWKDAEIVGLGAAAAA---------------------------------------------------------
LgnmMHCII1                      LVKNMDPLNENIVSIMQQA-SDTFMANLWKDAEIVGMGAAAAV---------------------------------------------------------
Aaesmnm                         LMKNMDPLNENIVSLLQGS-QDPFVVQIWKDAEIVGMAQQALT---------------------------------------------------------
Dme_zipper_gi62471805refNP_001  LMKNMDPLNENIVSLLQGS-QDPFVVNIWKDAEIVGMAQQALT---------------------------------------------------------
PpiMHCb1                        LTKNADPLNDNVLDLFRKS-ALPFMGALYDD---------------------------------------------------------------------
PpiMHCb2                        ----------------------------------------------------------------------------------------------------
Mmu_Myo15_gi261245016refNP_001  IGKNKGLLNETVVALLQKS-SNKVLANLFTKDIIAGSAS-------------------------------------------------------------
Mmu_Myo7B_gi145864471refNP_001  LEKNKDPLNETVVPIFQKS-QNRLLATLYENYAGSCSTEPPKS---------------------------------------------------------
MmcMHC7                         LQKNKDPLNETVVGLYQKS-SLKLLSNLFANYAGADAPA-------------------------------------------------------------
Mmu_Myo6_gi6754774refNP_034986  LEKNKDPLNETVVGLYQKS-SLKLMATLFSTYASADTGDS------------------------------------------------------------
Mmu_Myo13_gi124486959refNP_001  LDKNKDPLNETVVGLYQKS-SLKLLSFLFSNYAGAEAGDSA-----------------------------------------------------------
Mmu_Myo3_gi153792649refNP_0010  LEKNKDPLNETVVGLYQKS-SNRLLAHLYATFATTDADG-------------------------------------------------------------
MmskMHC8                        LDKNKDPLNDTVVGLYQKS-AMKTLASLFSTYASAEADG-------------------------------------------------------------
Mmu_Myo2_gi205830428refNP_0010  LDKNKDPLNETVVGLYQKS-SVKTLAYLFSGAQTAEAEASSGG---------------------------------------------------------
Mmu_Myo4_gi67189167refNP_03498  LDKNKDPLNETVVGLYQKS-GLKTLAFLFSGGQAAEAEGG------------------------------------------------------------
Mmu_Myo1_gi82524274refNP_10960  LDKNKDPLNETVVGLYQKS-SMKTLAYLFSGAAAAAEAESGGG---------------------------------------------------------
Hma_21579261_gi221113859refXP_  LEKNKDPINDHTASLFSHA-TDPLVSLLFQDYDPDQAAGRRKGSAFQTVSFRHKEQLKNLLTTLGMTSPHFVRCIIPNEKKEPGVVEGQLVLHQLRCNGV
Asumyo4                         LEKNKDPLNDTVVSCMKATQGNNLLNEVWKDYVTQEEAAIAA----------------------------------------------------------
AqMHCIIb                        LDKNKDPLNESVVELFRKS-SDPFVALLWGDYSF------------------------------------------------------------------
Aaesmnmmy                       LEKNKDPLNDTVVEQFKKG-ENALLVEIFSEAAPVVAEGAPP----------------------------------------------------------
DmeMHCC                         LEKNKDPLNDTVVDQFKKS-QNKLLIEIFADHAGQSGGGE------------------------------------------------------------
Lpemyosin                       LDKNKDPINENVVELLQNS-KEPIVKMLFTPPRI------------------------------------------------------------------
LgMHCII3                        LEKNKDPLNETIVELLMHS-KEALVQTLFAPSEPAAA---------------------------------------------------------------
TaMHCb                          LEKNKDPLNDSVVDLLRKS-TDPIIASLFAD---------------------------------------------------------------------
Nve_SB1                         LDKNKDPINEAVASLFAKS-GDPFISHLWKEY--------------------------------------------------------------------

                                       910       920       930       940       950       960       970       980       990      1000
                                ....|....|....|....|....|....|....|....|....|....|....|....|....|....|....|....|....|....|....|....|
Mmu_MyoX_gi130507685refNP_0623  ------------------------------------------------------------------TLKCGSKHRR---------P-TVSSQFKDSLHSL
Mmu_VIIa_gi115511010refNP_0326  ------------------------------------------------------------------AMGAETRKRS---------P-TLSSQFKRSLELL
Mmu_VIIb_gi261823963refNP_1157  ------------------------------------------------------------------AGSQLFKSSDSIKR----PV-TLASQFKQSLDQL
Ddi_0301_gi166240253refXP_0017  -----------------------------------------------------------GGAGGNKASSSAAGKST--FK----FT-SVSSQFKESLNSL
Ddi_1951_gi66823681refXP_64519  ------------------------------------------------------------GGPTSSNTKGTSGSSS--MK----FL-SVGSQFSTSLATL
Sce_9711_gi6324902refNP_014971  -----------------------------------------------------------LELEQAGSKKPGPIRTV--NR----KP-TLGSMFKQSLIEL
Sce_9373_gi6319290refNP_009373  ------------------------------------------------------------------TEKKIMIPARLSQK----KP-TLGSMFKKSLGEL
Mmu_MyoVc_gi124486759refNP_001  ------------------------------------------------------------------SAKQVIKPNT--KH----FRTTVGNKFRSSLYLL
Mmu_MyoVa_gi115511052refNP_034  ----------------------------------------------------------LTRVPVKPTKGRPGQTAK--EH----KK-TVGHQFRNSLHLL
Mmu_MyoVb_gi46399202refNP_9638  -----------------------------------------------------------SKINVRSSRPLIKVPNK--EH----KK-SVGYQFRTSLNLL
Ddimyo                          ------------------------------------------------------------------PNIASRAKKG--AN----FI-TVAAQYKEQLASL
Sce_1888_gi6321812refNP_011888  ------------------------------------------------------------------ISNQEVKKSARTST----FK-TTSSRHREQQITL
Sar_1270_SARC_01270T0Sphaerofo  ------------------------------------------------------------------SHDTTRSRRG--GS----MR-TVGQNYRDQLNSL
Mbr_4119_nullMonosigabrevicoll  ------------------------------------------------------------------SVASGSRTRK--GA----FR-TVGFIYKDQLSRL
Sro_4749_PTSG_04749T0Salpingoe  ------------------------------------------------------------------SLASANRTRK--GA----FR-TVGFIYKENLNHL
PpiMHCa                         ------------------------------------------------------------------TSGQTSRVRK--GM----FR-TVSQLYKQQLEKL
Aqu_8281_gi340368578refXP_0033  ------------------------------------------------------------------SAFGAQRSRK--GM----FR-TVGQLYKEQLSNL
Cow_3939_CAOG_03939T0Capsaspor  ------------------------------------------------------------------AFGATTRARK--GM----FR-TVAQIHKEQLEHL
TaMHCa                          ------------------------------------------------------------------TTFGSSRTRK--GM----FR-TVGQLYKEQLARL
Asumyo                          ------------------------------------------------------------------ETAFGVRTKK--GM----FR-TVSQMHKEQLTRL
Hma_21673571_gi221130996refXP_  ------------------------------------------------------------------TAFGAAKTKK--GM----FR-TLSQLYKEQLQGL
Nve_JGI5820_jgiNemve1118073jgi  ------------------------------------------------------------------ESPFGSKVRK--GM----FR-TVSQVYKDQLARL
Mmu_Myo14_gi29336026refNP_0822  ------------------------------------------------------------------DGPPGGRPRR--GM----FR-TVGQLYKESLSRL
MmsmMHC11                       ------------------------------------------------------------------SLPSASKTKK--GM----FR-TVGQLYKEQLGKL
Mmu_Myo9_gi114326446refNP_0718  ------------------------------------------------------------------ALPGAFKTRK--GM----FR-TVGQLYKEQLAKL
MmuMHC10                        ------------------------------------------------------------------AFGSAYKTKK--GM----FR-TVGQLYKESLTKL
Lpenm                           ------------------------------------------------------------------ETAFGSRTRK--GM----FR-TVSQLYKEQLAKL
```

```
LgnmMHCII1                      ------------------------------------------------------------------DTMFGSRTRK--GM----FR-TVSQLYKEQLSKL
Aaesmnm                         ------------------------------------------------------------------DTQFGARTRK--GM----FR-TVSHLYKEQLAKL
Dme_zipper_gi62471805refNP_001  ------------------------------------------------------------------DTQFGARTRK--GM----FR-TVSHLYKEQLAKL
PpiMHCb1                        ------------------------------------------------------------------CVPAGPKERK--KT----FQ-TMAMMHKKSLNEL
PpiMHCb2                        ----------------------------------------------------------------------------------------------------
Mmu_Myo15_gi261245016refNP_001  ------------------------------------------------------------------QFGEKTHKKG--TS----FH-LITSLHKENINKL
Mmu_Myo7B_gi145864471refNP_001  ------------------------------------------------------------------GVKE-KRKKA--AS----FQ-TVSQLHKENLNKL
MmcMHC7                         ------------------------------------------------------------------DKGKGKAKKG--SS----FQ-TVSALHRENLNKL
Mmu_Myo6_gi6754774refNP_034986  ------------------------------------------------------------------GKGKGGKKKG--SS----FQ-TVSALHRENLNKL
Mmu_Myo13_gi124486959refNP_001  ------------------------------------------------------------------GGKKGGKKKG--SS----FQ-TVSAVFRENLNKL
Mmu_Myo3_gi153792649refNP_0010  ------------------------------------------------------------------GKKKVAKKKG--SS----FQ-TVSALFRENLNKL
MmskMHC8                        ------------------------------------------------------------------GAKKGAKKKG--SS----FQ-TVSALFRENLNKL
Mmu_Myo2_gi205830428refNP_0010  ------------------------------------------------------------------AAKKGAKKKG--SS----FQ-TVSALFRENLNKL
Mmu_Myo4_gi67189167refNP_03498  ------------------------------------------------------------------GGKKGGKKKG--SS----FQ-TVSALFRENLNKL
Mmu_Myo1_gi82524274refNP_10960  ------------------------------------------------------------------GGKKGAKKKG--SS----FQ-TVSALFRENLNKL
Hma_21579261_gi221113859refXP_  LEGIRICRKGFPSRMNFQDFKLSYAGSVGYSVEGWLEKNKDPINDHTASLFSHATDPLVSLLFQDYDPDPAGRRKG--SA----FQ-TVSFRHKEQLKNL
Asumyo4                         ------------------------------------------------------------------KEGGGGKKHGKSGS----FM-TVSMLYRESLNNL
AqMHCIIb                        ------------------------------------------------------------------ESEKGSRKRG--SQ----FQ-TVGQIHKTSLNNL
Aaesmnmmy                       ------------------------------------------------------------------AKGGGRGKKG--AA----FA-TVSSAYKEQLNNL
DmeMHCC                         ------------------------------------------------------------------QAKGGRGKKG--GG----FA-TVSSAYKEQLNSL
Lpemyosin                       ------------------------------------------------------------------LTPGGKKKKGKSAA----FQ-TISSVHKESLNKL
LgMHCII3                        ------------------------------------------------------------------SGGPAKKKKS--SA----FQ-TISATHRESLNKL
TaMHCb                          ------------------------------------------------------------------HQPEGSRKKG--SQ----FL-TVSQLHKEQLEKL
Nve_SB1                         ------------------------------------------------------------------ATEGHTRGKG--GS----FQ-TVSAKHKEQLQSL

                                       1010      1020      1030      1040      1050      1060      1070      1080      1090      1100
                                ....|....|....|....|....|....|....|....|....|....|....|....|....|....|....|....|....|....|....|....|
Mmu_MyoX_gi130507685refNP_0623  MATLSSSNPFFVRCIKPNTQKMPDQFDQVVVLNQLRYSGMLETVRIRKAGYAVRRPFQDFYKRYK--VLMRNLALP------------------------
Mmu_VIIa_gi115511010refNP_0326  MRTLGACQPFFVRCIKPNEFKKPMLFDRHLCVRQLRYSGMMETIRIRHAGYPIRYSFVEFVERYR--VLLPGVKPAYKQ---------------------
Mmu_VIIb_gi261823963refNP_1157  MRILTNCQPYFVRCIKPNEYKKPLLFDRELCIQQLRYSGMMETVHIRKSGFPIRYTFDEFSQRFR--VLLPSPERMQFQ---------------------
Ddi_0301_gi166240253refXP_0017  MTTINSTNPHYIRCIKPNTEKRANLFDNVMVLHQLRCSGVIEQLRISRSGYPSRLVYDNFIKRYK--LIVAKDFKNDDDSNESKEWNSILKETDLNSSNG
Ddi_1951_gi66823681refXP_64519  MKTISTTTPHYVRCIKPNPEKLPQTFNKQDVIHQLRCGGVMESVRICCAGFPTRRLLSEFYQRYK--ILYVKDINTGSGGGK----------------KG
Sce_9711_gi6324902refNP_014971  MNTINSTNVHYIRCIKPNADKEAWQFDNLMVLSQLRACGVLETIRISCAGFPSRWTFEEFVLRYY--ILIPHEQWDLIFKKK------------------
Sce_9373_gi6319290refNP_009373  MAIINSTNVHYIRCIKPNSEKKPWEFDNLMVLSQLRACGVLETIRISCAGFPSRWTFDEFVQRYF--LLTDYSLWSGILYNP------------------
Mmu_MyoVc_gi124486759refNP_001  METLNATTPHYVRCIKPNDEKMPFEFDSKRIVQQLRACGVLETIRISAQSYPSRWTYLEFYSRYG--ILMTQQELSL-----------------------
Mmu_MyoVa_gi115511052refNP_034  METLNATTPHYVRCIKPNDFKFPFTFDEKRAVQQLRACGVLETIRISAAGFPSRWTYQEFFSRYR--VLMKQKDVLG-----------------------
Mmu_MyoVb_gi46399202refNP_9638  METLNATTPHYVRCIKPNDEKLPFHFDPKRAVQQLRACGVLETIRISAAGYPSRWTYHDFFNRYR--VLMKKRELTN-----------------------
Ddimyo                          MATLETTNPHFVRCIIPNNKQLPAKLEDKVVLDQLRCNGVLEGIRITRKGFPNRIIYADFVKRYY--LLAPNVPRDA-----------------------
Sce_1888_gi6321812refNP_011888  LNQLASTHPHFVRCIIPNNVKKVKTFNRRLILDQLRCNGVLEGIRLAREGYPNRIAFQEFFQRYR--ILYPENSTTTTFSSK------------------
Sar_1270_SARC_01270T0Sphaerofo  MTTLRNTTPHFVRCIIPNHEKKAGKIDQAIVLDQLRCNGVLEGIRICRKGFPNRILFQDFKQRYE--ILTPGAVPKG-F---------------------
Mbr_4119_nullMonosigabrevicoll  MDTLHHTVPNFVRCIIPNHKKRPGMIDAQLVLDQLACNGVLEGIRICRKGFPNRILFQEFRQRYS--ILTPDAVGPG-F---------------------
Sro_4749_PTSG_04749T0Salpingoe  MDTLNHTTPHFVRCIIPNHEKKAGKIDAPLVLDQLRCNGVLEGIRICRKGFPNRILFQEFKQRYS--ILVPEAIPRG-F---------------------
PpiMHCa                         MSILHDTKPHFVRCIIPNHEKRPGKMVAPLILEQLRCNGVLEGIRICRQGYPNRILFQEFRQRYE--ILTPNAIPTG-F---------------------
Aqu_8281_gi340368578refXP_0033  MITLRQTHPHFVRCIIPNYEKKAGKIVAPLVLDQLRCNGVLEGIRICRLGFPNRILFQEFRQRYE--ILTPGIIPKG-F---------------------
Cow_3939_CAOG_03939T0Capsaspor  MTTLRNTQPHFVRCIIPNHEKKAGKINNQLVLEQLRCNGVLEGIRIVRQGFPSRVLFQEFKQRYE--ILTPSAVPKGV----------------------
TaMHCa                          MTVLNNTSPNFVRCIIPNHDKKPGKIEANLVLEQLRCNGVLEGIRICRQGFPNRIAFQEFKQRYE--ILTPGAIPKG-F---------------------
Asumyo                          MTTLRNTSPHFVRCIIPNHEKKAGKITSMLVLEQLRCNGVLEGIRICRQGFPNRVPFQEFRHRYE--ILTPNVIPKG-F---------------------
Hma_21673571_gi221130996refXP_  MNTLNCTKPNFVRCIIPNYEKRSGKITNFLVLDQLRCNGVLEGIRICRQGFPNRILFQEFRQRYE--ILCPGVVPKG-F---------------------
Nve_JGI5820_jgiNemve1118073jgi  MLVLNNTNPNFVRCIIPNHEKKAGKIASFLVLDQLRCNGVLEGIRICRQGFPNRMLFQEFKQRYE--LLTPGVIPKG-F---------------------
Mmu_Myo14_gi29336026refNP_0822  MATLSNTNPSFVRCIVPNHEKRAGKLEPRLVLDQLRCNGVLEGIRICRQGFPNRILFQEFRQRYE--ILTPNAIPKG-F---------------------
MmsmMHC11                       MATLRNTTANFVRCIIPNHEKRSGKLDAFLVLEQLRCNGVLEGIRICRQGFPNRIVFQEFRQRYE--ILAANAIPKG-F---------------------
Mmu_Myo9_gi114326446refNP_0718  MATLRNTNPNFVRCIIPNHEKKAGKLDPHLVLDQLRCNGVLEGIRICRQGFPNRVVFQEFRQRYE--ILTPNSIPKG-F---------------------
MmuMHC10                        MATLRNTNPNFVRCIIPNHEKRAGKLDPHLVLDQLRCNGVLEGIRICRQGFPNRIVFQEFRQRYE--ILTPNAIPKG-F---------------------
Lpenm                           MATLRNTNPNFVRCIIPNHDKRPGKIEAPLVLDQLRCNGVLEGIRICRQGFPNRILFQEFRQRYE--ILTPSAIPKG-F---------------------
LgnmMHCII1                      MSTLRNTNPNFVRCIIPNHEKKAGKINSTLVLDQLRCNGVLEGIRICRQGFPNRIIFQEFRQRYE--ILTPNAIPRG-F---------------------
Aaesmnm                         MDTLRNTNPNFVRCIIPNHEKRAGKIDAPLVLDQLRCNGVLEGIRICRQGFPNRIPFQEFRQRYE--LLTPNVIPKG-F---------------------
Dme_zipper_gi62471805refNP_001  MDTLRNTNPNFVRCIIPNHEKRAGKIDAPLVLDQLRCNGVLEGIRICRQGFPNRIPFQEFRQRYE--LLTPNVIPKG-F---------------------
PpiMHCb1                        MGMLHSTSPNFIRCLVPNETKTPGKVLAELVLHQLRCNGVLEGIRIVRQGFPNRIPYADFKQRYQ--ILAASAIPAG-F---------------------
PpiMHCb2                        ----------------------------------------------------------------------------------------------------
Mmu_Myo15_gi261245016refNP_001  MTDLKSTAPHFVRCINPNKNKIPGVMDPFLVLQQLRCNGVLEGIRVCCEAFPSWMLYDDFKQRYW--ILNPRIFSKSKF---------------------
Mmu_Myo7B_gi145864471refNP_001  MTNLRATQPHFVRCIVPNENKTPGVMDSFLVLHQLRCNGVLEGIRICRQGFPNRLLYADFRQRYR--ILNPSAIPDDTF---------------------
MmcMHC7                         MTNLRSTHPHFVRCIIPNETKSPGVMDNPLVMHQLRCNGVLEGIRICRKGFPNRILYGDFRQRYR--ILNPAAIPEGQF---------------------
Mmu_Myo6_gi6754774refNP_034986  MTNLKTTHPHFVRCIIPNERKAPGVMDNPLVMHQLRCNGVLEGIRICRKGFPNRILYGDFRQRYR--ILNPAAIPEGQF---------------------
Mmu_Myo13_gi124486959refNP_001  MTNLRSTHPHFVRCLIPNETKTPGVMDHYLVMHQLRCNGVLEGIRICRKGFPSRILYADFKQRYR--ILNASAIPEGQF---------------------
Mmu_Myo3_gi153792649refNP_0010  MSNLRTTHPHFVRCIIPNETKTPGAMEHSLVLHQLRCNGVLEGIRICRKGFPNRILYGDFKQRYR--VLNASAIPEGQF---------------------
MmskMHC8                        MTNLRSTHPHFVRCIIPNETKTPGAMEHELVLHQLRCNGVLEGIRICRKGFPSRILYGDFKQRYK--VLNASAIPEGQF---------------------
Mmu_Myo2_gi205830428refNP_0010  MTNLRSTHPHFVRCIIPNETKTPGAMEHELVLHQLRCNGVLEGIRICRKGFPSRILYADFKQRYK--VLNASAIPEGQY---------------------
Mmu_Myo4_gi67189167refNP_03498  MTNLKSTHPHFVRCLIPNETKTPGAMEHELVLHQLRCNGVLEGIRICRKGFPSRILYADFKQRYK--VLNASAIPEGQF---------------------
Mmu_Myo1_gi82524274refNP_10960  MTNLRSTHPHFVRCIIPNETKTPGAMEHELVLHQLRCNGVLEGIRICRKGFPSRILYADFKQRYK--VLNASAIPEGQF---------------------
Hma_21579261_gi221113859refXP_  LTTLGMTSPHFVRCIIPNEKKEPGVVEGQLVLHQLRCNGVLEGIRICRKGFPSRMNFQDFKLRYEYQILASNAIPPG-F---------------------
Asumyo4                         MTMLNMTHPHFIRCIIPNEKKTSGLIEAGLVLNQLTCNGVLEGIRICRKGFPNRMLHPDFRHRYS--VLAADEANSS-----------------------
AqMHCIIb                        MTTLRNTTPHFVRCIIPNELKKAGVLEANLVLQQLRCNGVLEGIRICRKGFPNRLLYPEFRQRYA--ILAPKAIPAG-F---------------------
Aaesmnmmy                       MNTLMSTSPHFVRCIIPNELKQTGLIDAKLVMHQLTCNGVLEGIRICRKGFPNRMMYSDFKQRYL--ILAPAAM-QA-E---------------------
DmeMHCC                         MTTLRSTQPHFVRCIIPNEMKQPGVVDAHLVMHQLTCNGVLEGIRICRKGFPNRMMYPDFKMRYK--IMCPKLL-QG-V---------------------
Lpemyosin                       MKNLYSTHPHFVRCIIPNELKTPGLIDAALVLHQLRCNGVLEGIRICRKGFPNRIIYSEFKQRYS--ILAPNAVPSG-F---------------------
LgMHCII3                        MKNLYSTHPHFVRCIIPNEFKQPGEVDAHLVLHQLQCNGVLEGIRICRKGFPNRLVHSEFKQRYS--ILAPNAIPDG-F---------------------
TaMHCb                          MVNLRNTTPHFVRCIIPNEKKKPGIIEANLVLHQLRCNGVLEGIRICRKGFPNRIIFSEFKQRYA--ILAPGAIPSGMF---------------------
Nve_SB1                         MDTLYSTSPHFVRCIIPNELKKAGVIDTPLVIHQLRCNGVLEGIRICRKGFPNRIPFQEFKQRYQ--ILAPTAV-SGQF---------------------

                                       1110      1120      1130      1140      1150      1160      1170      1180      1190      1200
                                ....|....|....|....|....|....|....|....|....|....|....|....|....|....|....|....|....|....|....|....|
Mmu_MyoX_gi130507685refNP_0623  ------DDIRGKCTVLLQVY----DASNSEWQLGKTKVFLRESLEQKLEKRREEEIDRAAMVIRAHILGYLARKQYRK----------------------
Mmu_VIIa_gi115511010refNP_0326  ------GDLRGTCQRMAEAV----LGTHDDWQIGKTKIFLKDHHDMLLEVERDKAITDRVILLQKVIRGFKDRSNFLR----------------------
Mmu_VIIb_gi261823963refNP_1157  ------NKPRQMTLHIADLC----LGTDKDWKVGKTKIFLKDHQDTVLEIRRSQALDGAAIRIQRVLRGHKYRKEFLR----------------------
Ddi_0301_gi166240253refXP_0017  GTNNQIELKRKGAELMINKL----SIDISSVQFGLTKLFFKSGIIANLELLRSQTMINSATFIQKIWRGYTDRKAYTS----------------------
Ddi_1951_gi66823681refXP_64519  SNNNKIKDPKILVQNLLTGI----ELSDDKYKIGLTKVFLRAGQLASLEDMRLEQLDRSATVIQKRWKGYLYRKRYKQ----------------------
Sce_9711_gi6324902refNP_014971  --ETTEEDIISVVKMILDAT----VKDKSKYQIGNTKIFFKAGMLAYLEKLRSNKMHNSIVMIQKKIRAKYYRKQYLQ----------------------
Sce_9373_gi6319290refNP_009373  --DLPKEAIVNFCQSILDAT----ISDSAKYQIGNTKIFFKAGMLAFLEKLRTNKMNEICIIIQKKIRARYYRLQYLQ----------------------
Mmu_MyoVc_gi124486759refNP_001  ------SDKKEVCKVVLHRL----IQDSNQYQFGRTKIFFRAGQVAYLEKLRLDKLRQDCIMIQKHVRGWLQRRKFLR----------------------
Mmu_MyoVa_gi115511052refNP_034  -------DRKQTCKNVLEKL----ILDKDKYQFGKTKIFFRAGQVAYLEKLRADKLRAACIRIQKTIRGWLLRKRYLC----------------------
Mmu_MyoVb_gi46399202refNP_9638  ------TDKKNICKSVLESL----IKDPDKFQFGRTKIFFRAGQVAYLEKLRADKFREATIMIQKSVRGWLQRVKYRR----------------------
Ddimyo                          ------EDSQKATDAVLKHL----NIDPEQYRFGITKIFFRAGQLARIEEAREQRISEIIKAIQAATRGWIARKVYKQAREHTVAARIIQQNLRAYIDF-
Sce_1888_gi6321812refNP_011888  ----LKASTKQNCEFLLTSL----QLDTKVYKIGNTKLFFKAGVLADLEKQKDVKLNNIMIKLTATIRGYTVRKEITYHLQKLKKTRVIGNTFRLYNRLV
Sar_1270_SARC_01270T0Sphaerofo  ------MDGRKACEKMIEAL----ELSADEFRIGSSKIFFRTGVIGQLEEQRDEKLSAMVRHLQAHCRGLLARRQYRRATNHEVAVKMIQRNCRKYLML-
Mbr_4119_nullMonosigabrevicoll  ------TEGRTACERMLEAL----NLDPNSYRVGLTKVFFRAGVLAHLEEERDIRLSKMIVGLQAHCRGYLARRHHGALLHGSNAILVIQSNVRAYMKL-
Sro_4749_PTSG_04749T0Salpingoe  ------TDGRHACEKMVEAL----NLDAQSYRIGLTKVFFRAGILAQLEEQRDLKLSKMIIGLQAFCRGYLARRYHGALLHGSNAILVIQRNARAYMKL-
PpiMHCa                         ------MDGRKAATAMLSQL----DLDQRLYRIGKSKIFFRAGVLANLESERDEKLRVLIIGLQAMMRGKIALEKFKKLIQQQKAIQVLQRNGKCYLKL-
Aqu_8281_gi340368578refXP_0033  ------MDGKKAAEKMLIAL----ELDKATYRIGHSKVFFRAGVLAQLEEERDLKLTEILIGLQSHCRGFIGRKNFQKRVEQTRAIRIIQRNVQSYLKL-
Cow_3939_CAOG_03939T0Capsaspor  ------MDNKKICQKMVEAL----ELEANSFRIGHSKIFFRAGVLAQLEEQRDEKLTAVIKGFQAFCRGFMARRDLKKMMSNETAIRIIQRNTRKYLVL-
TaMHCa                          ------MDARKAVQKMTEVL----ELESNSYRIGLSKLFFRAGTLARLEEERDLKLSSIITGFQAYCRGNLARKMYLRRVQQTNAIRVIQRNVQAYLKL-
Asumyo                          ------MDGKEAVRKMIDAL----ELESNLYRIGQSKIFFRAGVLAHLEEERDLKLTDLIVSFQAQCRAFLARRLYQKRVQQSNAIRVLQRNGVAWLKL-
Hma_21673571_gi221130996refXP_  ------MDGRNASKKMIEAL----EMDPNLFRIGQSKIFFRAGVLAHLEEERDIKLTEIVIQFQAFCRGNIARKNYKRRIQQLSAIRVIQRNGRSWMKL-
Nve_JGI5820_jgiNemve1118073jgi  ------MDGRKACEKMLQSL----ELDSNSYRIGQSKIFFRAGVLAHLEEERDIKLTEIITIFQAFCRGNIARKQYHKRVQQLSAIRVIQRNCLSYLKL-
```

```
Mmu_Myo14_gi29336026refNP_0822  ------MDGKQACEKMIQAL----ELDPNLYRVGQSKIFFRAGVLAQLEEERDLKVTDIIVSFQAAARGYLARRAFQRRQQQQSALRVMQRNCAAYLKL-
MmsmMHC11                       ------MDGKQACILMIKAL----ELDPNLYRIGQSKIFFRTGVLAHLEEERDLKITDVIMAFQAMCRGYLARKAFTKRQQQLTAMKVIQRNCAAYLKL-
Mmu_Myo9_gi114326446refNP_0718  ------MDGKQACVLMIKAL----ELDSNLYRIGQSKVFFRAGVLAHLEEERDLKITDVIIGFQACCRGYLARKAFAKRQQQLTAMKVLQRNCAAYLRL-
MmuMHC10                        ------MDGKQACERMIRAL----ELDPNLYRIGQSKIFFRAGVLAHLEEERDLKITDIIIFFQAVCRGYLARKAFAKKQQQLSALKVLQRNCAAYLKL-
Lpenm                           ------MDGKKAVGKMIESL----ELDPNLYRIGQSKIFFRAGVLAHLEEERDLKLTDIIIQFQAYVRGMLARRNYHKRLQQLSAIRVIQRNCSAYLKL-
LgnmMHCII1                      ------MDGKKAVEKMIQAL----ELDKNLYRIGQSKIFFRAGVLAHLEEERDLKLTDIIVQFQALARGLLARRNYQKRLQQLNAIRVIQRNCAAYLKL-
Aaesmnm                         ------MDGKRACEQMIKAL----ELDNNLYRIGQSKIFFRAGVLAHLEEERDYKITDLIVNFQAYCRGFLARRNYQKRLQQLNAIRIIQRNCAAYLKL-
Dme_zipper_gi62471805refNP_001  ------MDGKKACEKMIQAL----ELDSNLYRVGQSKIFFRAGVLAHLEEERDFKISDLIVNFQAFCRGFLARRNYQKRLQQLNAIRIIQRNCAAYLKL-
PpiMHCb1                        ------IEAKAATELIIAAL----GLTDDEAKFGNTKVFFRAGILGRLEDLRDEKITEILTAFQALARGWIARKIFSKLMEQRVGVVVLQRNIRQHFIL-
PpiMHCb2                        ----------------------------------------------------------------------------------------------------
Mmu_Myo15_gi261245016refNP_001  ------VSSRKATEEVLDFL----EIDHPHYQCGVTKVFFKAFILDQLEERRDEKISKVFTLFQARARGKLMRITFQKILEERDALALIQENIRAFIAV-
Mmu_Myo7B_gi145864471refNP_001  ------VDSRKATEKLLGSL----DIDHTQYQFGHTKVFFKAGLLGILEELRDQRLAKVLTLLQARSRGRLMRLEYQRMLGGRDALFTIQWNIRAFNAV-
MmcMHC7                         ------IDSRKGAEKLLGSL----DIDHNQYKFGHTKVFFKAGLLGLLEEMRDERLSRIITRIQAQSRGVLSRMEFKKLLERRDSLLIIQWNIRAFMGV-
Mmu_Myo6_gi6754774refNP_034986  ------IDSRKGAEKLLGSL----DIDHNQYKFGHTKVFFKAGLLGLLEEMRDERLSRIITRIQAQARGQLMRIEFKKIVERRDALLVIQWNIRAFMGV-
Mmu_Myo13_gi124486959refNP_001  ------IDSKNASEKLLNSI----DVDREQFRFGHTKVFFKAGLLGLLEEMRDEKLVTLMTRTQAVCRGYLMRVEFKKMMERRESIFCIQYNVRSFMNV-
Mmu_Myo3_gi153792649refNP_0010  ------IDSKKACEKLLASI----DIDHTQYKFGHTKVFFKAGLLGTLEEMRDERLAKLITRTQAVCRGFLMRVEFQKMMQRRESIFCIQYNIRAFMNV-
MmskMHC8                        ------IDSKKASEKLLGSI----DIDHTQYKFGHTKVFFKAGLLGLLEEMRDEKLAQIITRTQAVCRGYLMRVEYQKMLLRRESIFCIQYNVRAFMNV-
Mmu_Myo2_gi205830428refNP_0010  ------IDSKKASEKLLGSI----DIDHTQYKFGHTKVFFKAGLLGLLEEMRDDKLAQLITRTQAMCRGFLARVEYQKMVERRESIFCIQYNIRAFMNV-
Mmu_Myo4_gi67189167refNP_03498  ------IDSKKASEKLLGSI----DIDHTQYKFGHTKVFFKAGLLGTLEEMRDEKLAQLITRTQAVCRGYLMRVEFKKMMERRESIFCIQYNVRAFMNV-
Mmu_Myo1_gi82524274refNP_10960  ------IDSKKASEKLLGSI----DIDHTQYKFGHTKVFFKAGLLGLLEEMRDDKLAQLITRTQAMCRGYLARVEYQKMVERRESIFCIQYNVRAFMNV-
Hma_21579261_gi221113859refXP_  ------IDGKVAAEKLIEAL----QLDQSEYRVGKTKIFFRAGIVGELEEMRDERLSKIISQFQAYCKGSIMRSEYKKMVAQRIGLAVIQRNVRKYLFL-
Asumyo4                         ------PDAKKCAEAILGKLVSQQKLTEDNYKMGDTKVFFKAGVLARLEDIRDEVLKVIMTKFEAYIRWYCGLIDRKRRIEQNAATLLLQRNIHMWCSL-
AqMHCIIb                        ------MDGRKATEKLIDAL----QLDQSEFRLGHSKVFFRAGVLGRLEDLRDERLSLVFGQFQVFCRGFIMRRKYRKLQEQRLAIAVIQRNVRKHLFL-
Aaesmnmmy                       ------AEPKKAAEKCFEAI----QLDPDSYRIGHTKVFFRAGVLGQMEDFRDQRLSKIMSWLQSWCRGYLTRIEFKKMQSQRTALEVVQRNLRKYLKL-
DmeMHCC                         ------EKDKKATEIIIKFI----DLPEDQYRLGNTKVFFRAGVLGQMEEFRDERLGKIMSWMQAWARGYLSRKGFKKLQEQRVALKVVQRNLRKYLQL-
Lpemyosin                       ------ADGKVVTDKVLSAL----QLDPNEYRLGNTKVFFKAGVLGMLEDMRDERLSKIISMFQAHIRGYLMRKAYKKLQDQRIGLTLIQRNVRKWLVL-
LgMHCII3                        ------VDGKTVAGKILVAL----EMDPNEYKLGSTKVFFKAGVLGYLEDLRDERLSSIIAMFQAHIRGYLVRRNYKKLCDKRIGLSVIQKNIRKWLAM-
TaMHCb                          ------MDGRKAAAKLVDAL----QLEPNEFRMGTTKVFFRAGVIGRLEESRDEKLYAILSKFQARMRGFLMRKTFQKMQEQRSGLQIIQRNVRKYIVL-
Nve_SB1                         ------MDGKKACEKLLAAI----QLEENEYRIGTTKVFFRAGVLGHLEDLRDDRLAKIISMFQAYCKGFLMRKEYRKMCDQRIGISVIQRNVRKYLYL-

                                        1210      1220      1230      1240      1250      1260      1270      1280      1290      1300
                                ....|....|....|....|....|....|....|....|....|....|....|....|....|....|....|....|....|....|....|....|
Mmu_MyoX_gi130507685refNP_0623  ----------------------------------------------------------------------------------------------------
Mmu_VIIa_gi115511010refNP_0326  ----------------------------------------------------------------------------------------------------
Mmu_VIIb_gi261823963refNP_1157  ----------------------------------------------------------------------------------------------------
Ddi_0301_gi166240253refXP_0017  ----------------------------------------------------------------------------------------------------
Ddi_1951_gi66823681refXP_64519  ----------------------------------------------------------------------------------------------------
Sce_9711_gi6324902refNP_014971  ----------------------------------------------------------------------------------------------------
Sce_9373_gi6319290refNP_009373  ----------------------------------------------------------------------------------------------------
Mmu_MyoVc_gi124486759refNP_001  ----------------------------------------------------------------------------------------------------
Mmu_MyoVa_gi115511052refNP_034  ----------------------------------------------------------------------------------------------------
Mmu_MyoVb_gi46399202refNP_9638  ----------------------------------------------------------------------------------------------------
Ddimyo                          KSWPWWKLFSKARPLLKRRNFEKEIKEKEREILELKSNLTDSTTQKDKLEKSLKDTESNVLDLQRQLKAEKETLKAMYDSKDALEAQKRELEIRVEDMES
Sce_1888_gi6321812refNP_011888  KEDPWFNLFIRIKPLLTSSNDMTRTKKFNEQINKLKNDLQEMESKKKFLEEKNQKTVNELENTQDLLNQEKENLRKNESLLNRVKTSSETLQKQFDDLVS
Sar_1270_SARC_01270T0Sphaerofo  RSWPWWKLFVRVQPLLKVTQEDN----LKKELEDAKASAEHSAKREQELQVQFDELHNEKNGLKSDLDRVQNELDSCNDSIDRFQTRSEQLLKEVEGLED
Mbr_4119_nullMonosigabrevicoll  RSWAWWKLFTRLKPLLKHARSDDEMRELREKVDQLQEQLQAERQAREAAENEADKMRRKANALEDDIQVCAST--------RNLVLFMRNLWCLFHGIDH
Sro_4749_PTSG_04749T0Salpingoe  RSWAWWKLFTKIKPLLKVARGDEELRLLREELEDVKQQLERSELARKEAEARIETLEMEKKELNQQLEADNVALTDAEEVRSRLVSKNNELLEDLQYVEQ
PpiMHCa                         RNWQWWRLFTKVKPLLQVTSRDKEIHEREEELKKVQDAHVRDREALQENEAQLKKTIEERATLQDQIFREKEMAAEADEQRTRLVNKKIELEEYIHDLEE
Aqu_8281_gi340368578refXP_0033  RNWKWWRLFTKVKPLLQVTNAEEQKREMEEEIKKLNASYEKLRIEYDELAKKNNVVLVNNKQLEDQLREEKFLTQEAEEMRDMLAQKRTELEGMIGEYEL
Cow_3939_CAOG_03939T0Capsaspor  RNWAWWKLYTKVKPLLNVARQEDEMRQKEQEVKKLQEKAEKEEAARIEMEKLHAKLLEEKNALATQLQLESEAAAEAEEMKNRLAAKRAELEGIVGELEA
TaMHCa                          RDWQWWRLYTKVRPLLNVTRQDDEKRQVEDELKRVREKSEKAQHDLQILEIDYNKVLEEKSILAEQLQTETELCAEAEEMRIRLSIKKNELEELLHDAEV
Asumyo                          RNWQWWRLFTKVKPLLQVTNQEAAIAAKDEELKVVKEKLNRREVEFVEVEKRIQQLSEERAVLQEQLQQESEERAEADELRERLTARKVELEEMLTELNT
Hma_21673571_gi221130996refXP_  RNWQWWRLFTKVKPLLNVTRHDEELRAKEEEYKKVLDKYEKVESAHNDLKKSHDKLTDENKLLAEQLQAEIELCQEAEENVSCLQQRKIELEELLNDFEI
Nve_JGI5820_jgiNemve1118073jgi  RNWQWWRLFTKVKPLLNVTRQEEELHQREDELRTLREKLEKLETEYTDLEKKHSQISEEKAIVAEQLESEREVAQETEEMRQRLQTKKNELEELLSDLEG
Mmu_Myo14_gi29336026refNP_0822  RNWQWWRLFIKVKPLLQVTRQDEVLQARAQELQKVQELQQQSAREVGELQGRVAQLEEERTRLAEQLRAEAELCSEAEETRARLAARKQELELVVTELEA
MmsmMHC11                       RNWQWWRLFTKVKPLLQVTRQEEEMQAKEEEMQKITERQQKAETELKELEQKHTQLAEEKTLLQEQLQAETELYAESEEMRVRLAAKKQELEEILHEMEA
Mmu_Myo9_gi114326446refNP_0718  RNWQWWRLFTKVKPLLNSIRHEDELLAKEAELTKVREKHLAAENRLTEMETMQSQLMAEKLQLQEQLQAETELCAEAEELRARLTAKKQELEEICHDLEA
MmuMHC10                        RHWQWWRVFTKVKPLLQVTRQEEELQAKDEELLKVKEKQTKVEGELEEMERKHQQLLEEKNILAEQLQAETELFAEAEEMRARLAAKKQELEEILHDLES
Lpenm                           RNWQWWRLFTKVKPLLSVTNQEEKLNAKEEELKKIKEHYEKTKFEYEDLERKFANLVDEKSLLAEQLQAETELCTEAEETRILLNNRKIELEEVLNETEA
LgnmMHCII1                      RNWQWWRLFTKVKPLLSVTGQEEQMVKKEEETKRMKESLEKQQQDLLEFEKRFSQVIEEKNILAEQLQAETELCAEAEEARARLAARKQELEDVMHDMEL
Aaesmnm                         RNWQWWRLYTKVKPLLEVTKQEEKLVQKEDELRQIRDKLDNLSRSSQEYEKKFQQAIEEKTHLAEQLQAEIELCAEAEEGRARLAARKQELEELMQDLEA
Dme_zipper_gi62471805refNP_001  RNWQWWRLYTKVKPLLEVTKQEEKLVQKEDELKQVREKLDTLAKNTQEYERKYQQALVEKTTLAEQLQAEIELCAEAEESRSRLMARKQELEDMMQELET
PpiMHCb1                        RNWPWWKLFTKVKPLLSVARQEEEIREKEEEMKRAMEAAEETAKAKKELEEKFEKLQSERNEIQAELEKERQAAIDAEELVFKLEGKQKDLEEENNELID
PpiMHCb2                        ----------------------------------------------------------------------------------------------------
Mmu_Myo15_gi261245016refNP_001  KNCPWMGLFFKIKPLAKSVGAGEEIAGLKEECAQLQKALESSESQREELKTKQVSLVQEKNDLRLQLQAEQETLANSEEQCESLIKSKVELEVKIKELSR
Mmu_Myo7B_gi145864471refNP_001  KNWSWMKLFFKMKPLLRSAQAEEELAALRAELRGLRGALATAEAKRQELEETQVSVTQEKNDLALQLQAEQDNLADAEERCHLLIKSKVQLEAKVKELSE
MmcMHC7                         KNWPWMKLYFKIKPLLKSAETEKEMATMKEEFGRVKDALEKSEARRKELEEKMVSLLQEKNDLQLQVQAEQDNLADAEERCDQLIKNKIQLEAKVKEMTE
Mmu_Myo6_gi6754774refNP_034986  KNWPWMKLYFKIKPLLKSAETEKEMANMKEEFGRVKDALEKSEARRKELEEKMVSLLQEKNDLQLQVQAEQDNLNDAEERCDQLIKNKIQLEAKVKEMTE
Mmu_Myo13_gi124486959refNP_001  KHWPWMNLFFKIKPLLKSAEAEKEMATMKEDFERAKEDLARSEARRKELEEKMVSLLQEKNDLQLQVQSETENLMDAEERCEGLIKSKIQLEAKVKELNE
Mmu_Myo3_gi153792649refNP_0010  KHWPWMKLFFKIKPLLKSAETEKEMATMKEEFQKTKDELAKSEAKRKELEEKLVTLVQEKNDLQLQVQAESENLLDAEERCDQLIKAKFQLEAKIKEVTE
MmskMHC8                        KHWPWMKLFFKIKPLLKSAETEKEMATMKEEFQKTKDELAKSEAKRKELEEKMVTLLKEKNDLQLQVQSEADSLADAEERCEQLIKNKIQLEAKIKEVTE
Mmu_Myo2_gi205830428refNP_0010  KHWPWMKLFFKIKPLLKSAETEKEMATMKEEFQKTKDDLAKSEAKRKELEEKMVSLLKEKNDLQLQVQAEAEGLADAEERCDQLIKTKIQLEAKIKEVTE
Mmu_Myo4_gi67189167refNP_03498  KHWPWMKLYFKIKPLLKSAETEKEMANMKEDFEKAKEDLAKSEAKRKELEEKMVALMQEKNDLQLQVQAEADGLADAEERCDQLIKTKIQLEAKIKELTE
Mmu_Myo1_gi82524274refNP_10960  KHWPWMKLYFKIKPLLKSAETEKEMANMKEEFEKAKENLAKAEAKRKELEEKMVALMQEKNDLQLQVQSEADSLADAEERCDQLIKTKIQLEAKIKEVTE
Hma_21579261_gi221113859refXP_  RHWSWWKLYTKVQPLLSVARAEDEMRAKEEELEAAKEQLKKDAEAKKKMEEELTEAMAQKEKLYASLQAETDRLITIEDKLLNLQTVKDKLESSLNEALE
Asumyo4                         RTWEWFRLYTKVRPMLREGKIAEQMEKLNEKLKSLEDGIEKEAKLRKELEDNSVKTQAEKADLLAQLESVKAQLDEAEERVKRESALKGDVDKQLEDLNE
AqMHCIIb                        RDWRWWKLFTKVKPLLNVARAEDELRQKEEEVTKLREKASKDDQARKAMEEDLTRLMQEKQQLYDQLQKEQDAAAESEDKLSKLIIKKNELEDNFKELVD
Aaesmnmmy                       RTWAWWKLWQKVKPLLNVSRVEDQIAKLEEKAQKATEAFEKEEKLRKELEALNSKLLAEKTALLDSLSGEKGALQEYQEKAAKLTAQKNDLENQLRDTQE
DmeMHCC                         RTWPWYKLWQKVKPLLNVSRIEDEIARLEEKAKKAEELHAAEVKVRKELEALNAKLLAEKTALLDSLSGEKGALQDYQERNAKLTAQKNDLENQLRDIQE
Lpemyosin                       RNWEWWRLFNKVKPLLNIARQEDENKKAQEEFAKMKEEFASCEQMRKELEEQNTVLMQQKNDLVIAMSSGEDAIGDAEEKIEQLIKQKSDFETQIKELED
LgMHCII3                        RNWLWWKMYVKVKPLLNVARAEDEMKQKEEQFEKTKVELEKSEKMKKELEEQNTVLLQQKNDMFLELQTEQDKVVEFEEKVENLIKQKIDFEAQIKEMEE
TaMHCb                          RNWQWWRLFTKVKPLLNVARSDEELKHMQEELNRTLDKSQKEESLRKDLEEQNTKLVEEKQQLFMDLQREQDAYADAEDKIVKLESIKNDMESQIQELND
Nve_SB1                         RNWAWWKLYTRVKPLLQVARADEEMKQKVEQMKEIEEKLGKEEALRKELEEKYTKLVEEKNLLFQDFQREQDACADAEERNAELEGRKADLEAQVKDMLE

                                        1310      1320      1330      1340      1350      1360      1370      1380      1390      1400
                                ....|....|....|....|....|....|....|....|....|....|....|....|....|....|....|....|....|....|....|....|
Mmu_MyoX_gi130507685refNP_0623  ----------------------------------------------------------------------------------------------------
Mmu_VIIa_gi115511010refNP_0326  ----------------------------------------------------------------------------------------------------
Mmu_VIIb_gi261823963refNP_1157  ----------------------------------------------------------------------------------------------------
Ddi_0301_gi166240253refXP_0017  ----------------------------------------------------------------------------------------------------
Ddi_1951_gi66823681refXP_64519  ----------------------------------------------------------------------------------------------------
Sce_9711_gi6324902refNP_014971  ----------------------------------------------------------------------------------------------------
Sce_9373_gi6319290refNP_009373  ----------------------------------------------------------------------------------------------------
Mmu_MyoVc_gi124486759refNP_001  ----------------------------------------------------------------------------------------------------
Mmu_MyoVa_gi115511052refNP_034  ----------------------------------------------------------------------------------------------------
Mmu_MyoVb_gi46399202refNP_9638  ----------------------------------------------------------------------------------------------------
Ddimyo                          ELDEKKLALENLQNQKRSVEEKVRDLEEELQEEQKLRNTLEKLKKKYEEELEEMKRVNDGQSDTISRLEKIKDELQKEVEELTESFSEESKDKGVLEKTR
Sce_1888_gi6321812refNP_011888  ----------------------------------------------------------------------------------------------------
Sar_1270_SARC_01270T0Sphaerofo  QLEEESSRADNLMTEKKALQAKQSIVEAEMADAQQRAEKLAGEKVSLEEKLSAVETELLSEKDANEKLTKDKASLESQLSTRSEELAAAEEKYKSLSKAK
Mbr_4119_nullMonosigabrevicoll  RIEELTDSSARLNEQNNVLSEEIAHLEEQLAQEMDSLQTYLIEKKDLLAQLEDMRGGLDVASVNEARATRLEEQLSEARTKSEETQQE------------
Sro_4749_PTSG_04749T0Salpingoe  QLDEQMSTTEKLLAEKKHLIEELEDLKKDLGTANLNEARISRLEEQLETVRKQLDDQTAA----------------------------------------
PpiMHCa                         RIDTEEEKTQKVLSEKKKIESTIADLETTLADEGNARQKLSIEKQQLENKLKQLEETVANKDDSIGKIARDRAHLETRLKDTQSALEEEEDKGKALAKQR
Aqu_8281_gi340368578refXP_0033  RLEESDEQNNQLVSDWKKLKQQLQETEEQLEEEGTSNQKLMQEKVSAENKIKALEEQITINDDNISKLSREKNHLDDKVREIQAALEQEEGKAKQEHRQR
```

```
Cow_3939_CAOG_03939T0Capsaspor  RLDEEEEIKAKLSTEKRKLEAQINEMEEKVNDLETIKQKLEQEKAAREAQLKKIEDELTVEKDNVEKLTKERNSLNEKLTTTSASLQDQEDTNKHLIKQK
TaMHCa                          KAAEEEENVNVLTKEKKKLLQSISDLETSLEEEEAQRQKLQMEKASLDSKIVAFENDLMKEADTNSKLSKDKKFLEDKVAELTSSFGTLEDKHKHLNRQK
Asumyo                          KLDEGEEQMEKLSEEKKKLQETVRDLEEQLEEEEQARQKLMLDKVNVDQKWKKLEEKHAELYDSYEKLLKEKKNLEERTVQLSNQVLEEEEKAKHVGKQR
Hma_21673571_gi221130996refXP_  KLAEEEERSAKTAEEKKKLQQGIQELEESLEEEEAQRQKLQLEKVQVEAKLKALEDDLRLVEDSNAKLSQDKKQLEERMDELSTKLSAEEDKSKGLIKLK
Nve_JGI5820_jgiNemve1118073jgi  RITEEEENVLALTEDKKKLLKEIQDLEDSLEEEESARQKLHLEKVSCEAKIKKLEEDLSLVEDTNSKISKEKKTLEEKLNELDQALIDEEEKSKGLAKQK
Mmu_Myo14_gi29336026refNP_0822  RVGEEEECSRQLQSEKKRLQQHIQELESHLEAEEGARQKLQLEKVTTEAKMKKFEEDLLLLEDQNSKLSKERRLLEERLAEFSSQAAEEEEKVKSLNKLR
MmsmMHC11                       RLEEEEDRRQQLQAERKKMAQQMLDLEEQLEEEEAARQKLQLEKVTAEAKIKKLEDDILVMDDQNSKLSKERKLLEERVSDLTTNLAEEEEKAKNLTKLK
Mmu_Myo9_gi114326446refNP_0718  RVEEEEERCQYLQAEKKKMQQNIQELEEQLEEEESARQKLQLEKVTTEAKLKKLEEDQIIMEDQNCKLAKEKKLLEDRVAEFTTNLMEEEEKSKSLAKLK
MmuMHC10                        RVEEEEERNQILQNEKKKMQAHIQDLEEQLDEEEGARQKLQLEKVTAEAKIKKMEEEVLLLEDQNSKFIKEKKLMEDRIAECSSQLAEEEEKAKNLAKIR
Lpenm                           RLEEEMRNSEFLHDEKNKLRLTIEDLEEQLEEEEQARQKLQMEKLSVDQRVKKLEEDIAVNTDSGNKLSKEKKYLEEKIHELTSKLVGEEDKFKQLGKQK
LgnmMHCII1                      RVEEEEERYNQVLEEKKNYQQTLKDLEEQLEEEEQARQKLQLERVSHESKLKKYEEDLALLDDQNQKLNKEKKNLEERLADISLQAVEEEEKAKQLGKLK
Aaesmnm                         RIEEEEERVNALSSEKKKLQINIQDLEEQLEEEEAARQKLQLEKVQLDAKLKKMEEDVALTEDQNQKLLKEKKILEERANDLSQTLAEEEEKAKHLAKLK
Dme_zipper_gi62471805refNP_001  RIEEEEERVLALGGEKKKLELNIQDLEEQLEEEEAARQKLQLEKVQLDAKIKKYEEDLALTDDQNQKLLKEKKLLEERANDLSQTLAEEEEKAKHLAKLK
PpiMHCb1                        RIEEEEENNFSVAEMKRAIEAELAKTQEEVKVLEXXXXXXXXXXXXXXXXXXXXXXXXXXXXXXXXXXXXXXXXXXXXXXXXXXXXXXXXXXXEKASKLDKER
PpiMHCb2                        ----------------------------------------------------------------------------------------------------
Mmu_Myo15_gi261245016refNP_001  QVEEEEEINSELTARGRKLEDECSELKKEIYDLEAILAKSEKGKCAAEHKVRNLTEEVHSLNEEVSKLSRVVKDAQETQQQTQEQLHIEEEKLSNMSKAN
Mmu_Myo7B_gi145864471refNP_001  RLEDEEEVNADLAARRRKLEDECTELKKDIDDLELTLAKAEKEKQATENKVKNLTEEMAALDEAVVRLTKEKKALQEAHQQALGDLQAEEDRVSALAKAK
MmcMHC7                         RLEDEEEMNAELTAKKRKLEDECSELKRDIDDLELTLAKVEKEKHATENKVKNLAEEMAGLDEIIVKLTKEKKALQEAHQQALDDLQAEEDKVNTLTKAK
Mmu_Myo6_gi6754774refNP_034986  RLEDEEEMNAELTAKKRKLEDECSELKKDIDDLELTLAKVEKEKHATENKVKNLTEEMAGLDEIIAKLTKEKKALQEAHQQALDDLQAEEDKVNTLTKSK
Mmu_Myo13_gi124486959refNP_001  RLEEEEEMNSELVAKKRNLEDKCSSLKRDIDDLELTLTKVEKEKHATENKVKNLSEEMTALEETISKLTKEKKSLQEAHQQTLDDLQVEEDKVNGLIKIN
Mmu_Myo3_gi153792649refNP_0010  RAEDEEEINAELTAKKRKLEDECSELKKDIDDLELTLAKVEKEKHATENKVKNLTEELAGLDETIAKLTREKKALQEAHQQTLDDLQAEEDKVNSLSKLK
MmskMHC8                        RAEDEEEINAELTAKKRKLEDECSELKKDIDDLELTLAKVEKEKHATENKVKNLTEEMAGLDENIAKLTKEKKALQEAHQQTLDDLQAEEDKVNTLTKAK
Mmu_Myo2_gi205830428refNP_0010  RAEDEEEINAELTAKKRKLEDECSELKKDIDDLELTLAKVEKEKHATENKVKNLTEEMAGLDETIAKLTKEKKALQEAHQQTLDDLQAEEDKVNTLTKAK
Mmu_Myo4_gi67189167refNP_03498  RAEDEEEINAELTAKKRKLEDECSELKKDIDDLELTLAKVEKEKHATENKVKNLTEEMAGLDENIAKLTKEKKALQEAHQQTLDDLQAEEDKVNTLTKAK
Mmu_Myo1_gi82524274refNP_10960  RAEDEEEINAELTAKKRKLEDECSELKKDIDDLELTLAKVEKEKHATENKVKNLTEEMAGLDETIAKLTKEKKALQEAHQQTLDDLQAEEDKVNTLTKAK
Hma_21579261_gi221113859refXP_  KLDGEEHSVLVLEEKIQEAEEKIDELTEKTEELQSNISRLETEKQNRDKQIDTLNEDIRKQDETISKMNAEKKHVDEELKDRTEQLQAAEDKCNNLNKTK
Asumyo4                         QLAQTEDKNEDLMRAKKKVESEVEALKKQIQDLEMSVRKAESEKQSKDHQVRSIQDEMQMQDETIAKLNKEKKHQEEMNKKIMEDLQSEEDKSNHINKIK
AqMHCIIb                        RLDEEMEVNSEMSAGKRKLEIEIESFKDTIDEMKQQQQLLEQEKAQKEKECVSLDVELEKVNDSLSKITKEKLSLEDRLNEVSNSLQTEEDKASRLGKMK
Aaesmnmmy                       RLAQEEDARNQLFQTKKKLEQEISGQKKDAEDLELQIQKIEQDKASKDHQIRNLNDEIAHQDELINKLNKEKKMQGEVNQKTAEELQAAEDKVNHLNKVK
DmeMHCC                         RLTQEEDARNQLFQQKKKADQEISGLKKDIEDLELNVQKAEQDKATKDHQIRNLNDEIAHQDELINKLNKEKKMQGETNQKTGEELQAAEDKINHLNKVK
Lpemyosin                       KLMDEEDAATELSAQKKKSDAEIGELKKDVEDLEAGLAKAEQEKTTKDNQIKTLQDEMAQQDEHLSKLNKEKKNLEEVQKKTLEDLQAEEDKVNHLSKLK
LgMHCII3                        RCLDEEDAVGNLTEAKKQLQKANDDLKKDLEDMETNLVKAEQEKTSKDNQIKTLNDEIAKQEDMIMKVTKDKKNMEEANKKLSDSLQAEEDKVNHLSKLK
TaMHCb                          QLEEEEDNNAQISQVKRKLEAEVENLRTDIEDIEAALQKSDQEKQAKDDALRSITTELDECQEAVDALTKDKKALEEKELELSNLLEAEEDKSNHLTKVK
Nve_SB1                         QLEDEEEASAELSSVKHKLEGEISDLKQDIEELDATLKKVEEEGKQKDKNIEQLNEELQQQDEAIAKLQKAKKQVEDERTELEDHLQEEQNKVSHLTKTK

                                         1410      1420      1430      1440      1450      1460      1470      1480      1490      1500
                                ....|....|....|....|....|....|....|....|....|....|....|....|....|....|....|....|....|....|....|....|
Mmu_MyoX_gi130507685refNP_0623  ----------------------------------------------------------------------------------------------------
Mmu_VIIa_gi115511010refNP_0326  ----------------------------------------------------------------------------------------------------
Mmu_VIIb_gi261823963refNP_1157  ----------------------------------------------------------------------------------------------------
Ddi_0301_gi166240253refXP_0017  ----------------------------------------------------------------------------------------------------
Ddi_1951_gi66823681refXP_64519  ----------------------------------------------------------------------------------------------------
Sce_9711_gi6324902refNP_014971  ----------------------------------------------------------------------------------------------------
Sce_9373_gi6319290refNP_009373  ----------------------------------------------------------------------------------------------------
Mmu_MyoVc_gi124486759refNP_001  ----------------------------------------------------------------------------------------------------
Mmu_MyoVa_gi115511052refNP_034  ----------------------------------------------------------------------------------------------------
Mmu_MyoVb_gi46399202refNP_9638  ----------------------------------------------------------------------------------------------------
Ddimyo                          VRLQSELDDLTVRLDSETKDKSELLRQKKKLEE----ELKQVQEALAAETAAKLAQEAANKKLQGEYTELNEKFNSEVTARSNVEKSKKTLESQLVAVNN
Sce_1888_gi6321812refNP_011888  ----------------------------------------------------------------------------------------------------
Sar_1270_SARC_01270T0Sphaerofo  SKGDGSLAEIEERLATVEAERSDLQTQKRKIDA----ELATLRDRVAELEKQVAQLQAEVERKTAEVAATQEQLDAESEKVFAVQKENRELTSQVESLQE
Mbr_4119_nullMonosigabrevicoll  ----------------------------------------------------------------------------------------------------
Sro_4749_PTSG_04749T0Salpingoe  ----------------------------------------------------------------------------------------------------
PpiMHCa                         VKLETAMAELEEKLAKEEKSREDLDRQKRKLEN----VNHTLQDSVLEKENRVEELKNSLDKAMEDVARLTVKMDDDAATRSNLEKKLRELENRASDLSE
Aqu_8281_gi340368578refXP_0033  VKLEQLSAELDEKLERETKRRQELDNDKRKLQK----EIADLQEQLARAKQRIEDLESQVAKLETELANVNARADEEAAGRAKAEKEKRELATQLQETQD
Cow_3939_CAOG_03939T0Capsaspor  AKLDATITELEERLQKEEKERQELEKQKRKLES----DVRDLKEQLNAAGGEKGDLASTLGKKQAELEAINSALEEEKVQRSNVEKAKRDLQAQLEEVNE
TaMHCa                          AKVDSQLQETEDTLRKEQELRTDLEKIKRKLEA----DLRDTNDQLSDATAQVTELQNTLERHSNDLNQANAKIDQEVNARNKAEKKCRDLESQLQEITE
Asumyo                          SRVEVQLHDLEQELQKEKQAKAELDLQKRKLEAEMDEEMDEQKELLEEKRAKLEELNSLLSKREEELTVLLTKSDEDSANNVGLQKQIRELESTLEELKE
Hma_21673571_gi221130996refXP_  VKQETLISELEERVAKSEKTAVDYQKEIRKLQQ----EISDLRNQLAEAQQRIAELEDDLARRDNELAAAIKRGDEEMSKRVNVEKAKRDVEAHLEEVKD
Nve_JGI5820_jgiNemve1118073jgi  AKQDAMIADLEERLKNEERARQDLDKIRRKLES----ELAEVSRQLEEAKQQIQELEAAVHKKDMELNDLNARYDEEMARRTELEKDKRELENSVEELKE
Mmu_Myo14_gi29336026refNP_0822  LKYEATISDMEDRLKKEEKGRQELEKLKRRLDG----ESSELQEQMVEQKQRAEELLAQLGRKEDELQAALLRAEEEGGARAQLLKSLREAQAGLAEAQE
MmsmMHC11                       SKHESMISELEVRLKKEEKSRQELEKLKRKLEG----DASDFHEQIADLQAQIAELKMQLAKKEEELQAALARLDEEIAQKNNALKKIRELEGHISDLQE
Mmu_Myo9_gi114326446refNP_0718  NKHEAMITDLEERLRREEKQRQELEKTRRKLEG----DSTDLSDQIAELQAQIAELKMQLAKKEEELQAALARVEEEAAQKNMALKKIRELETQISELQE
MmuMHC10                        NKQEVMISDLEERLKKEEKTRQELEKAKRKLDG----ETTDLQDQIAELQAQVDELKVQLTKKEEELQGALARGDDETLHKNNALKVARELQAQIAELQE
Lpenm                           SRAESALADLETQVQRETQNRQELEKVKRKLEA----DLADHRDQLNEKRAQVEEVQSQLARREEELQSALQKSEDESVAKGTTLKQLRELQSQIQELSE
LgnmMHCII1                      NKYEAIIADLEERLRKEQQARQELEKIRRRLET----ELNDLRDQLNEKRDQVEELQQQLLRREEEVQMSLQKTDEEAGQRSILQKQMRELQNQIQELQE
Aaesmnm                         AKHESTIAELEERLLKDHQQRQESDRSKRKIET----EVADLKEQINERRMQIEEMQQQLIKREEELAQTLVRIDEESAAKAAAQKAQRELESQLAEIQE
Dme_zipper_gi62471805refNP_001  AKHEATISELEERLHKDQQQRQESDRSKRKIET----EVADLKEQLNERRVQVDEMQAQLAKREEELTQTLLRIDEESATKATAQKAQRELESQLAEIQE
PpiMHCb1                        KRVEGELNDTRNDLEKEKRAKAELDKNLRKKDA----EHKDVVGQLDDMTRQKGEAEDTIRRKEGEISTLQATVEAEQNNVTKAQRQIKDLGQKYKDLED
PpiMHCb2                        ----------------------------------------------------------------------------------------------------
Mmu_Myo15_gi261245016refNP_001  LKLAQQIDVLEGDLERERKARMKCEREKRKLQD----ELKMNQEGAENLESSRQKLAEQLRKKEFEMGQMNSKVENEKNQVSQLQKMVKELQTHILNLKE
Mmu_Myo7B_gi145864471refNP_001  IRLEQQVEDLECSLEQEKKLRMDTERAKRKLEG----DLKLTQETVTDTTQDKQQLEEKLKKKDSELSQLNLRVEDEQLVGVQLQKKIKELQARAEELEE
MmcMHC7                         VKLEQQVDDLEGSLEQEKKVRMDLERAKRKLEG----DLKLTQESIMDLENDKQQLDERLKKKDFELNALNARIEDEQALGSQLQKKLKELQARIEELEE
Mmu_Myo6_gi6754774refNP_034986  VKLEQQVDDLEGSLEQEKKVRMDLERAKRKLEG----DLKLTQESIMDLENDKLQLEEKLKKKEFDISQQNSKIEDEQALALQLQKKLKENQARIEELEE
Mmu_Myo13_gi124486959refNP_001  VKLEQQTDDLEGSLEQEKKLRADLERVKRKLEG----DLKMSQESIMDLENDTQQLEEKLKKKEFEMSQLQTRIDDEQVLSLQLQKKIKELQARTEELEE
Mmu_Myo3_gi153792649refNP_0010  SKLEQQVDDLESSLEQEKKLRVDLERNKRKLEG----DLKLAQESILDLENDKQQLDERLKKKDFEYSQLQSKVEDEQTLSLQLQKKIKELQARIEELEE
MmskMHC8                        TKLEQQVDDLEGSLEQEKKLRMDLERAKRKLEG----DLKLAQESTMDIENDKQQLDEKLKKKEFEISNLISKIEDEQAVEIQLQKKIKELQARIEELEE
Mmu_Myo2_gi205830428refNP_0010  IKLEQQVDDLEGSLEQEKKLRMDLERAKRKLEG----DLKLAQESIMDIENEKQQLDERLKKKEFEMSNLQSKIEDEQAIGIQLQKKIKELQARIEELEE
Mmu_Myo4_gi67189167refNP_03498  TKLEQQVDDLEGSLEQEKKLRMDLERAKRKLEG----DLKLAQESTMDIENDKQQLDEKLKKKEFEMSNLQSKIEDEQALGMQLQKKIKELQARIEELEE
Mmu_Myo1_gi82524274refNP_10960  IKLEQQVDDLEGSLEQEKKIRMDLERAKRKLEG----DLKLAQESTMDVENDKQQLDEKLKKKEFEMSNLQSKIEDEQALGMQLQKKIKELQARIEELEE
Hma_21579261_gi221113859refXP_  NKLESSIREIEQDLKKEKDSKMKLEKEKKKVES----DLKDNRDKLSETETRLKETQDLVTKREKSISDLENAKEGLESQISQLQRKIQELLAKIEELEE
Asumyo4                         AKLEQTLDDLEENLERERRAKADTEKARRKVEG----ELKIAQENIEEATRQKHDLENNLKRKEAEMNNLSSRLEDEQSIVSKLQRQIKEAQSRVGELEE
AqMHCIIb                        TKLESSLQDAQEEIEKEKKAVSDLDKAKRKLEG----DLKSMTSNCDELSAAKAELEKSVSSKEKEVNELQQKIEEDSSEIAALQRKIRELQAKIEELEE
Aaesmnmmy                       AKLEQTLDELEDSLEREKKLRGDVEKAKRKVEG----DLKLTQEAVSDLERNKKEMDQTIMRKDKEISALSAKLEDEQNLVGKLQKQIKELQGRIEELEE
DmeMHCC                         AKLEQTLDELEDSLEREKKVRGDVEKSKRKVEG----DLKLTQEAVADLERNKKELEQTIQRKDKELSSITAKLEDEQVVVLKHQRQIKELQARIEELEE
Lpemyosin                       TKLEQTLDELEDNLEREKKIPGDVDKAKRKVEQ----DLKTTQETVEDLERVKRDLEDAGRKKDMEINGLNSKLEDEQNLVAQLQKKIKELQARIEELEE
LgMHCII3                        SKLEQSLDEFENNLEREKKIRGDVEKVKRKLEQ----DLKMTQESVEDLERIKRELEETMRKKDAEINSLNSRVEDEQGLVASLQKKIKELQAIIEELEE
TaMHCb                          NKLETSLEELQSHLDSEKKHTSDLDKAKRKLES----EAKVANEKINEMEGNVKLAEETIHKRDNEISKLGQSLEDERTTVAALQKKLREAQSRIDELEE
Nve_SB1                         LKLESTLDEVNLNLEREKKVRGEVEKVKRKLEG----DLKMTQQTLEETQAEKARTEDEVRKRDANIVELSGKLEDSNNLVESLRKRIR-----------

                                         1510      1520      1530      1540      1550      1560      1570      1580      1590      1600
                                ....|....|....|....|....|....|....|....|....|....|....|....|....|....|....|....|....|....|....|....|
Mmu_MyoX_gi130507685refNP_0623  ----------------------------------------------------------------------------------------------------
Mmu_VIIa_gi115511010refNP_0326  ----------------------------------------------------------------------------------------------------
Mmu_VIIb_gi261823963refNP_1157  ----------------------------------------------------------------------------------------------------
Ddi_0301_gi166240253refXP_0017  ----------------------------------------------------------------------------------------------------
Ddi_1951_gi66823681refXP_64519  ----------------------------------------------------------------------------------------------------
Sce_9711_gi6324902refNP_014971  ----------------------------------------------------------------------------------------------------
Sce_9373_gi6319290refNP_009373  ----------------------------------------------------------------------------------------------------
Mmu_MyoVc_gi124486759refNP_001  ----------------------------------------------------------------------------------------------------
Mmu_MyoVa_gi115511052refNP_034  ----------------------------------------------------------------------------------------------------
Mmu_MyoVb_gi46399202refNP_9638  ----------------------------------------------------------------------------------------------------
Ddimyo                          ELDEEKKNRDALEKKKKALDAMLEEMKDQLESTGGEKKSLYDLKVKQESDMEALRNQISELQSTI-AKLEKIKSTLEGEVARLQGELEAEQLAKSNVEKQ
Sce_1888_gi6321812refNP_011888  ----------------------------------------------------------------------------------------------------
```

```
Sar_1270_SARC_01270T0Sphaerofo   ELESEQSALSKLKKEKLALDLLLKELEDRMEDSETSGNAQAEMRAKREEELQGLKTKYEEAQAKHEEVVGELKAKLQAQREELVAEVEQLQRNKSELDKN
Mbr_4119_nullMonosigabrevicoll   ----------------------------------------------------------------------------------------------------
Sro_4749_PTSG_04749T0Salpingoe   ----------------------------------------------------------------------------------------------------
PpiMHCa                          DLEAEKTAHRRTDKMKRDLNDELQKISNELMTSVDNKNVLQELKGNMEKDVEDLKKDLAKEKDEHEALIQSMRTKYQTQVEDLQERMETQKKQSASYEKQ
Aqu_8281_gi340368578refXP_0033   DLESEREARNKSEKNRKQLNAELEKLQELLDDTTSSTTAQQAMTAQRESELSALKRTLDEEISSHEAAIASMRQKHTRNVEDLNEQLDNAKKAKSMLEKV
Cow_3939_CAOG_03939T0Capsaspor   ELESERAARSKAEKRAKDLAADYDSLRAQLDESGDSNKMQAELRAKREAELASLKKKLDDETASHEAALTDLRKKHTSLAEESNAEIDSLKKSKAALEKN
TaMHCa                           DLDAERDTRSKIDKQKRQLNEELESLKNELLDSLDTTAAQQDLRNKREEELKHLKKVLEEDTIAHAEAVSLSKKKHNETIEEMNNQIETLKKAKASLEKY
Asumyo                           DLENEKSLRAKAEKSRRDLNDDLETLRTEYLEATDKTAVSLEIQKKKDGELKELKKALEAATAANESKIEELRQKYLRSMEDLSEQIEQQKRLRAQADKS
Hma_21673571_gi221130996refXP_   DYEQEKAAREKVEKAKRELEKDLNELREELEVNTDATTVQKELQRKREEDFNELKKQVEIEAREHEKQMDSLRGKHNQLVNELQDQLDQFKKGKNALEKN
Nve_JGI5820_jgiNemve1118073jgi   DLEVEKSARAKAEKQRRELSEELESLKSELEESIDTTAAATDMRVKREAELVALKKSVEEESAKHENLMSELRQKNSKQIEELTIELESTKKNKNSLEKS
Mmu_Myo14_gi29336026refNP_0822   DLEAERVARAKAEKQRRDLGEELEALRGELEDTLDSTNAQQELRSKREQEVTELKKALEEESRAHEVSMQELRQRHSQALVEMAEQLEQARRGKGVWEKT
MmsmMHC11                        DLDSERAARNKAEKQKRDLGEELEALKTELEDTLDSTATQQELRAKREQEVTVLKKALDEETRSHEAQVQEMRQKHTQAVEELTEQLEQFKRAKANLDKS
Mmu_Myo9_gi114326446refNP_0718   DLESERASRNKAEKQKRDLGEELEALKTELEDTLDSTAAQQELRSKREQEVSILKKTLEDEAKTHEAQIQEMRQKHSQAVEELADQLEQTKRVKATLEKA
MmuMHC10                         DFESEKASRNKAEKQKRDLSEELEALKTELEDTLDTTAAQQELRTKREQEVAELKKALEDETKNHEAQIQDMRQRHATALEELSEQLEQAKRFKANLEKN
Lpenm                            DLDAEKQSREKAEKQKRDLNEELEALKSELEDSLDTTAAVQELRNKRELEVDNLKKAIEENQKQGENQALELRQKYTKQLEAVNEDLDVIKKSKASIEKV
LgnmMHCII1                       DLETEKESRNKAEKQKRDLNEELEALKTELEDSLDTTAAVQDVRQKREHEVSEMKKVVEATQKQHENQIQDMRNKYSAQIEALSEEIENTRKTKGSVEKV
Aaesmnm                          DLEAEKLARSKAEKQKRDLNEELEALKNELLDSLDTTAAQQELRSKREQEVATLKKTLEDESANHEGQVSDMRHKHAQEISSINEQLENLKKVKGGLEKA
Dme_zipper_gi62471805refNP_001   DLEAEKAARAKAEKVRRDLSEELEALKNELLDSLDTTAAQQELRSKREQELATLKKSLEEETVNHEGVLADMRHKHSQELNSINDQLENLRKAKTVLEKA
PpiMHCb1                         EMDNERNAKNRLEKLRAELEGQIAAYEDQIEEAGGANNAASELCRRREDEIAKLRKEKEAVELEAQEQADSSKRKLNDVTNQLEDAEDMLRKYKAKAEKE
PpiMHCb2                         ------------------------------------------------------------------------------------------KKNKIKNEKD
Mmu_Myo15_gi261245016refNP_001   ELESERTIRAKVEREKGDLVQDLEDLNERLEEAGGTSLAQMEITKQQEARFQKLHHDMEETTRHFEATSASLKKRHAENLAELEGQVEHLQQVRLVLEQD
Mmu_Myo7B_gi145864471refNP_001   ELEAERAARARVEKQRAEAARELEELSERLEEAGGASAGQREGCRKREAELGRLRRELEEAVLRHEATVAALRRKQADSAAELSEQVDSLQRIRQKLEKE
MmcMHC7                          ELEAERTARAKVEKLRSDLSRELEEISERLEEAGGATSVQIEMNKKREAEFQKMRRDLEEATLQHEATAAALRKKHADSVAELGEQIDNLQRVKQKLEKE
Mmu_Myo6_gi6754774refNP_034986   ELEAERTARAKVEKLRSDLSRELEEISERLEEAGGATSVQIEMNKKREAEFQKMRRDLEEATLQHEATAAALRKKHADSVAELGEQIDNLQRVKQKLEKE
Mmu_Myo13_gi124486959refNP_001   EIEAEHTVRAKIEKQRSDLARELEEISERLEEASGATSAQIEMNKKRESEFQKLRRDLEEATLQHEATAATLRKKHADTVAELGEQIDNLQRVKQKLEKE
Mmu_Myo3_gi153792649refNP_0010   EIEAERATRAKTEKQRSDYARELEELSERLEEAGGVTSTQIELNKKREAEFLKLRRDLEEATLQHEATVATLRKKHADSAAELAEQIDNLQRVKQKLEKE
MmskMHC8                         EIEAERASRAKAEKQRSDLSRELEEISERLEEAGGATSAQVEMNKKRETEFQKLRRDLEEATLQHEATAAALRKKHADSVAELGEQIDNLQRVKQKLEKE
Mmu_Myo2_gi205830428refNP_0010   EIEAERASRAKAEKQRSDLSRELEEISERLEEAGGATSAQIEMNKKREAEFQKMRRDLEEATLQHEATAATLRKKHADSVAELGEQIDNLQRVKQKLEKE
Mmu_Myo4_gi67189167refNP_03498   EIEAERASRAKAEKQRSDLSRELEEISERLEEAGGATSAQIEMNKKREAEFQKMRRDLEEATLQHEATAAALRKKHADSVAELGEQIDNLQRVKQKLEKE
Mmu_Myo1_gi82524274refNP_10960   EIEAERASRAKAEKQRSDLSRELEEISERLEEAGGATSAQIEMNKKREAEFQKMRRDLEEATLQHEATAATLRKKHADSVAELGEQIDNLQRVKQKLEKE
Hma_21579261_gi221113859refXP_   ELENERKLRQKSELQRKELESRIEELQDQLETAGGATSAQVEVGKKREAECNRLRKEIEALNIANDAAISAIKAKTNATIAEIQEENEAMKKAKAKLEKE
Asumyo4                          ELEGERESRSKADRAKSDLQREIEELNERLDEQGGATEAQIEINKKREAELAKLRRDIEEANMNHEGQLATLRKKHGDAVAELADQLDMIEKQRQKLEKE
AqMHCIIb                         DLENEKRIRQKVEKQKNELLKELEELKDTLEEQGGIQTAQVELNRKRESELDTLRKEHAAATEESEKMISDLKKKHGESVKDYESQIDTLQKAKNKLEKE
Aaesmnmmy                        EVEAERQARAKAEKQRADLARELEELGERLEEAGGATSAQIELNKKREAELAKLRRDLEEANIQHEGTLANLRKKHNDAVAEMAEQVDQLNKLKTKAEHD
DmeMHCC                          EVEAERQARAKAEKQRADLARELEELGERLEEAGGATSAQIELNKKREAELSKLRRDLEEANIQHESTLANLRKKHNDAVAEMAEQVDQLNKLKAKAEKE
Lpemyosin                        ELEAERQARTKVEKQRTELSRELEELGERLDEAGGATAAQMELNKKREQELLRLRRDLEEATMQHESQIATLRKKNQEATNELGDQIDQLQKVKSRLEKE
LgMHCII3                         ELASEKQARSKVEKQRNELSHELEDISDRLDEAGGATSAQIDINKKRELELLKLRRELEENQLHHDAQIASFRKKQQESANEMSDEIDQLMKIKSKLEKE
TaMHCb                           EFDNERVLKLKIEKQKAELLQELDELNEKLDEAGGAHVLQTDLTKKREAEINRLQSEIESRTKQHGSQVASMKKKHNEIIDELENQVETLQRAKNKLESE
Nve_SB1                          ----------------------------------------------------------------------------------------------------

                                          1610      1620      1630      1640      1650      1660      1670      1680      1690      1700
                                 ....|....|....|....|....|....|....|....|....|....|....|....|....|....|....|....|....|....|....|....|
Mmu_MyoX_gi130507685refNP_0623   ----------------------------------------------------------------------------------------------------
Mmu_VIIa_gi115511010refNP_0326   ----------------------------------------------------------------------------------------------------
Mmu_VIIb_gi261823963refNP_1157   ----------------------------------------------------------------------------------------------------
Ddi_0301_gi166240253refXP_0017   ----------------------------------------------------------------------------------------------------
Ddi_1951_gi66823681refXP_64519   ----------------------------------------------------------------------------------------------------
Sce_9711_gi6324902refNP_014971   ----------------------------------------------------------------------------------------------------
Sce_9373_gi6319290refNP_009373   ----------------------------------------------------------------------------------------------------
Mmu_MyoVc_gi124486759refNP_001   ----------------------------------------------------------------------------------------------------
Mmu_MyoVa_gi115511052refNP_034   ----------------------------------------------------------------------------------------------------
Mmu_MyoVb_gi46399202refNP_9638   ----------------------------------------------------------------------------------------------------
Ddimyo                           KKKVELDLEDKSAQLAEETAAKQALDKLKKKLEQELSEVQTQLSEANNKNVNSDSTNKHLETSFNNLKLELEAEQKAKQALEKKRLGLESELKHVNEQLE
Sce_1888_gi6321812refNP_011888   ----------------------------------------------------------------------------------------------------
Sar_1270_SARC_01270T0Sphaerofo   ANALSSEVEALKESLDSEGKSRADSDKKKKSLDAQLSETTSILEETTRQLSETSAKAASLAAEVDALSKSLDEKDQALTKAERDAAQARAKVDDLTTTLE
Mbr_4119_nullMonosigabrevicoll   ----------------------------------------------------------------------------------------------------
Sro_4749_PTSG_04749T0Salpingoe   ----------------------------------------------------------------------------------------------------
PpiMHCa                          KNDLSKERNSLAEELDLAKQQKAELDRKKKMLESTVNENLAKIEECEKGSAMMVENHKKLTAELENLLQKQMDAESKVSMLERTNKDLNRQINEIQDSYD
Aqu_8281_gi340368578refXP_0033   KNSLETERSNLSSDLKDATSSLSESEKRRRNVEAQLSEAQAHIAEDTAKVQELTSQNERMKAEIANISSQLEELETKFTSSDRNNKNYLGQIQELQDQVS
Cow_3939_CAOG_03939T0Capsaspor   VSDATRDRDSLQSELDALKKAYAELEKKKKALEAQLAEATGRLNEDQRIIAELQSQNQKLTAELDSTSKNLEDLESRFSALEKAKKTAESSLGESKGNIE
TaMHCa                           KLQADSSLADVKAECSDLHASKTELEKKKKALESQVSDLNGRNTTLQRKTEDLEQTNSKLQSQLDTTVAQLVQAETQISSLERTSKSLELQIAELQELYE
Asumyo                           RRAVEAERNEIAAELTAMQSMKAEVEKRRKHAESALLEMTTRLKDLEGARTISMVQLSKAQNELEAAVKQKEEEEQNVSALLKKISLLEQQLAETHDQLQ
Hma_21673571_gi221130996refXP_   KTALENENAEIAADLQRVSLLKQESDRKAKNFEAQLSEANAIRMGQEEMISKLDGQCAKLTKECDSLNQQIDEVESKAANLERAKQAAETSLSDLQDALH
Nve_JGI5820_jgiNemve1118073jgi   KKDLENETSSLTIELKAISQEKQEGDRKIKMLENQMSEANIRLADDEQKVSELTISRNQLQKDLEVQISMVESLESKHTALERSKKSLEDTLAETQDSLQ
Mmu_Myo14_gi29336026refNP_0822   RLSLEAEVSELKAELSSLQTSRQEGEQKRRRLESQLQEVQGRSSDSERARSEAAEKLQRAQAELESVSTALSEAESKAIRLGKELSSAESQLHDTQELLQ
MmsmMHC11                        KQTLEKENADLAGELRVLGQAKQEVEHKKKKLEVQLQDLQSKCSDGERARAELSDKVHKLQNEVESVTGMLNEAEGKAIKLAKDVASLGSQLQDTQELLQ
Mmu_Myo9_gi114326446refNP_0718   KQTLENERGELANEVKALLQGKGDSEHKRKKVEAQLQELQVKFSEGERVRTELADKVTKLQVELDSVTGLLSQSDSKSSKLTKDFSALESQLQDTQELLQ
MmuMHC10                         KQGLETDNKELACEVKVLQQVKAESEHKRKKLDAQVQELHAKVSEGDRLRVELAEKANKLQNELDNVSTLLEEAEKKGIKFAKDAAGLESQLQDTQELLQ
Lpenm                            KESLQAENADLANDLKQMQQAKQESERKRKQLESHLQEQSMKLVDVERNKSELGEKLTKMQTEYEQAVASLEDSDASVLELNRKVSNLQTDLATATEGLE
LgnmMHCII1                       KQTLEAENQDLANDLKQVQQAKQESERKRKQLDSQLQEAVIKLQELERNKGEVSEKSTKLQSELESVSSQLEQSDTKFLQMTQKASALEAQLADSQDMCQ
Aaesmnm                          KQTLEAENADLATELRNVNQSRQENDRRRKQAETQIAELQVKLAEVERARMELQDKVTKLQQESENITQQLDDAELKASAAIKSASNMESQLTEAQQLLE
Dme_zipper_gi62471805refNP_001   KGTLEAENADLATELRSVNSSRQENDRRRKQAESQIAELQVKLAEIERARSELQEKCTKLQQEAENITNQLEEAELKASAAVKSASNMESQLTEAQQLLE
PpiMHCb1                         KKAFVSEMADLQEQIDNLAKAKSTSDKNSSGLDASLAEANARIAALEASLRELTDKYNKLVKDNNATLSRLEETEAREANLSKQKKVLSGQIDELKSQLE
PpiMHCb2                         RKGAQEEIDHLNETLDTLQKQKGNLDKTVRVLDEQLGDANSRCSQMETNLRELADKYNKLVKENNDFAQKIEDMEAKEAMLSKGKKGLQSQIDDLGGQLE
Mmu_Myo15_gi261245016refNP_001   KSDLQLQVDDLLNRVDQMARAKANAEKLCGLYERRLNEANTKLDEVTQLAHDLTTQKTKLQSESGEFFKRLEEKEALISQLSREKSNFTRQVEELRAQLE
Mmu_Myo7B_gi145864471refNP_001   KSELRMEVDDLGASVETLARGKASAEKLCRTYEDQLSEAKIKVEELQRQLADASTQRGRLQTENGELGRLLEEKESMISQLSRGKTSAAQSLEELRRQLE
MmcMHC7                          KSEFKLELDDVTSNMEQIIKAKANLEKMCRTLEDQMNEHRSKAEETQRSVNDLTSQRAKLQTENGELSRQLDEKEALISQLTRGKLTYTQQLEDLKRQLE
Mmu_Myo6_gi6754774refNP_034986   KSEFKLELDDVTSNMEQIIKAKANLEKVSRTLEDQANEYRVKLEEAQRSLNDFTTQRAKLQTENGELARQLEEKEALISQLTRGKLSYTQQMEDLKRQLE
Mmu_Myo13_gi124486959refNP_001   KSELKMEIDDMASNIETVSKSKSNMERMCRSVEDQFNEIKAKDDQQTQLIHDLNMQKARLQTQNGELSHQVEEKESLVSQLTKSKQALTQQLEELKRQLE
Mmu_Myo3_gi153792649refNP_0010   KSEFKLEIDDLSSSVESVSKSKANLEKICRTLEDQLSEARGKNEEMQRSLSELTTQKSRLQTEAGELSRQLEEKESIVSQLSRSKQAFTQQIEELKRQLE
MmskMHC8                         KSELKMEIDDLSSNAEAIAKAKGNLEKMCRTLEDQVSELKSKEEEQQRLINELTAQRARLQTEAGEYSRQLDEKDALVSQLSRSKQASTQQIEELKRQLE
Mmu_Myo2_gi205830428refNP_0010   KSEMKMEIDDLASNVETVSKAKGNLEKMCRTLEDQVSELKSKEEEQQRLINDLTTQRGRLQTESGEFSRQLDEKEALVSQLSRGKQAFTQQIEELKRQLE
Mmu_Myo4_gi67189167refNP_03498   KSELKMEIDDLASNMETVSKAKGNLEKMCRTLEDQLSEVKTKEEEQQRLINELSTQKARLHTESGEFSRQLDEKDAMVSQLSRGKQAFTQQIEELKRQLE
Mmu_Myo1_gi82524274refNP_10960   KSEMKMEIDDLASNMEVISKSKGNLEKMCRTLEDQVSELKTKEEEQQRLINELTAQRGRLQTESGEYSRQLDEKDSLVSQLSRGKQAFTQQIEELKRQLE
Hma_21579261_gi221113859refXP_   KSALNNELNETKNSLDQIKKQKTNSDKNSRMLEEQINELNSKLAQVDELHSQSESKNSKVNSELLALNSQLSESEHNLGIATKNIKTLESQLAESKNFNE
Asumyo4                          KAQIVHDSENLAAQLDSETAAKSNNEKLAKQLEMQLADLQGKADEQSRQLQDFASIRNRLANENADLNRQLEDLEEQLGALQRVKAQLSTQLEDTRQAAD
AqMHCIIb                         RSSLSSNQDDLNKQVEDLQKYKTVTERKVRQLEEQLADTSARATEAESAVQALEASQAKSATENNDLSQQLADADSKLSSLSKAKKQLETQLEDTKSDLS
Aaesmnmmy                        RANMYNELNNTRSACDQLSREKAAQEKIAKQLQHTLNEVQGKLDETNRTLNDFDAAKKKLSIENSDLLRQLEDAESQVSQLSKIKISLTQQLEDTKRLAD
DmeMHCC                          KNEYYGQLNDLRAGVDHITNEKAAQEKIAKQLQHTLNEVQSKLDETNRTLNDFDASKKKLSIENSDLLRQLEEAESQVSQLSKIKISLTTQLEDTKRLAD
Lpemyosin                        KTQLRAEMDDVQSQVEHAGKNRGCSEKMSKQMEAQLSELNAKIDDQARSVSELTSQKSRLQTEAADLTRQLEEAEHNVGQLTKLKSSLGASLEDAKRSLE
LgMHCII3                         RNHARSEAEELAAQLQHASKNKGMSEKMTKQVENQISEFHFKLEESTRIVSDLQSSKSKLQMEVSEITRTLEETEHKVSIIIKEKNTIQSQFEETKRCLE
TaMHCb                           KQHLSGDLDELQSNLESLTKAKNSSDKQLRQLDEQYNEASAKVDEQEKIIADLQAALQRLQAENNELSEQVEDGESKINSLTKAKKGLESQLEELKRKFE
Nve_SB1                          ----------------------------------------------------------------------------------------------------

                                          1710      1720      1730      1740      1750      1760      1770      1780      1790      1800
                                 ....|....|....|....|....|....|....|....|....|....|....|....|....|....|....|....|....|....|....|....|
Mmu_MyoX_gi130507685refNP_0623   ----------------------------------------------------------------------------------------------------
Mmu_VIIa_gi115511010refNP_0326   ----------------------------------------------------------------------------------------------------
Mmu_VIIb_gi261823963refNP_1157   ----------------------------------------------------------------------------------------------------
Ddi_0301_gi166240253refXP_0017   ----------------------------------------------------------------------------------------------------
Ddi_1951_gi66823681refXP_64519   ----------------------------------------------------------------------------------------------------
Sce_9711_gi6324902refNP_014971   ----------------------------------------------------------------------------------------------------
Sce_9373_gi6319290refNP_009373   ----------------------------------------------------------------------------------------------------
```

```
Mmu_MyoVc_gi124486759refNP_001 ----------------------------------------------------------------------------------------------------
Mmu_MyoVa_gi115511052refNP_034 ----------------------------------------------------------------------------------------------------
Mmu_MyoVb_gi46399202refNP_9638 ----------------------------------------------------------------------------------------------------
Ddimyo EEKKQKESNEKRKVDLEKEVSELKDQIEEEVASKKAVTEAKNKKESELDEIKRQYADVVSSRDKSVEQLK-TLQAKNEELRNTAEEAEGQLDRAERSKKK
Sce_1888_gi6321812refNP_011888 ----------------------------------------------------------------------------------------------------
Sar_1270_SARC_01270T0Sphaerofo DETRQKLNFQSKLKHAEGWTDEAKEALAECEEEVANRDRQVSSLKGQLDELTNRFRTEQGN-AENGEAARKNLMREVEELRTSVEGGIAAQSKLKSTNNR
Mbr_4119_nullMonosigabrevicoll --------------------------------------------------------------MAVITEEKAKLNKEISGLREELDQALNQKARAVKAQKK
Sro_4749_PTSG_04749T0Salpingoe --------------------------------------------------------------NQELEEQRKKLNKELNDVRADLDQALAQKSKAQKAHKK
PpiMHCa SESRDKQTLMAKMRDLQATHNQTLESLEEEQDARDVLQQKLTTSMQQLSDAKRAAEEHAGS-LEEVENARRKLSRDQEDLNAQIEDLRNEISKLEKSKKR
Aqu_8281_gi340368578refXP_0033 EESRLKIAAQNKQKQLQEEVDHLTQQVEDEEEAKTALQNKLVQLTQQLSEAKRKADDDQVN-LEELQASKKKVDKEMDGLKERVEELQAENQKLTRSKKK
Cow_3939_CAOG_03939T0Capsaspor AETRAKLALASKLKQAETDIAGLKEQLEEEEEAKAALEKQIASLTAQSADYKRKAD-DAGNQAEEFEATRKKLGKDADELRTRIEELEHALANSDKTKNR
TaMHCa DETKQKQNISMQHRELENKLSHHSELIEELESDKENLARQINTQSTQLADARKSLQQSEIA-LEDAENAKRKLAKDLDLCQNTLDDLKNTNTKLEKAKTR
Asumyo EETRSKLAMQSRVRQLEDELTESVEMREEVETKRKELEAQIESMRATLIETRKKADEGA---LQQMEELKKKAQRDLETAQKAIAEAEMARDRADRSKKK
Hma_21673571_gi221130996refXP_ EETRQKLALQTKVREAEDEANRLQEQLEEEEDEKKAVQKTLTQVQLQLDSCKKEIELKVTL-LEEAESARQKQKRENEELRSDNERFQSEISKLDKARKK
Nve_JGI5820_jgiNemve1118073jgi EETHHKLMIISKLKDVENEIVSLQEQLEEEEENKSALQKQLQSAQTQCAEMKKVVEERAAA-LEEADAVKKKLVREMEALNTTVEEIQGSNAKLEKTRKR
Mmu_Myo14_gi29336026refNP_0822 EETRAKLALGSRVRALEAEAAGLREQMEEEVVARERAGRELQSTQAQLSEWRRRQEEEAAV-LEAGEEARRRAAREAETLTQRLAEKTEAVERLERARRR
MmsmMHC11 EETRQKLNVSTKLRQLEDERNSLQDQLDEEMEAKQNLERHVSTLNIQLSDSKKKLQDFAST-IEVMEEGKKRLQKEMEGLSQQYEEKAAAYDKLEKTKNR
Mmu_Myo9_gi114326446refNP_0718 EENRQKLSLSTKLKQMEDEKNSFREQLEEEEAKRNLEKQIATLHAQVTDMKKKMEDGVGC-LETAEEAKRRLQKDLEGLSQRLEEKVAAYDKLEKTKTR
MmuMHC10 EETRQKLNLSSRIRQLEEEKNSLQEQQEEEEEARKNLEKQVLALQSQLADTKKKVDDDLGT-IESLEEAKKKLLKDVEALSQRLEEKVLAYDKLEKTKNR
Lpenm EENRLKMQALSDLRTATEERESLHDKLEEEEENKKLLQKQLEKNQSLMVELKKNAETAESSRDNAEEELKKAL-RLIEQLRQQIADLQEQIARLEKAKKK
LgnmMHCII1 EETRQKLAAQSKLRQALDEKESLLERLEEEEEVKKQLQKQCNDLQQKIVEVKKKAEEDAVN-NEALEEFKKKAARDADNYQREVEESKMQADRLDKSRRK
Aaesmnm EETRQKLALSSKLRQLESEKEALQEQLEEDEEAKKNYEKKLAELNFTIQEMKRRSEEDSDI-AKELEEAKKKMNKDIEVLQRQILELQATNDRLDKSKKK
Dme_zipper_gi62471805refNP_001 EETRQKLGLSSKLRQIESEKEALQEQLEEDDEAKRNYERKLAEVTTQMQEIKKKAEEDADL-AKELEEGKKRLNKDIEALERQVKELIAQNDRLDKSKKK
PpiMHCb1 DESSQRNSLANQLRGASADLQAARDEIEIGNQEKNDLKRNLGNAQSEAAGWKSRLESEALPKIDELEAEKRKLAGRIQDAEDRADEALSKCNNLDKVRSR
PpiMHCb2 TLNSEKNGVQTQLKQALAEIEGLQEEVEMATSEKEDKERMLGILQSELNTARNAITNEHVPRIEELEGENRKLQVKITELEDAVDEAQTKANNAEKTKNR
Mmu_Myo15_gi261245016refNP_001 EESRSQSALSHALQSAKHDYDLLREQYEEEQEVKAELHRALSKGNKETVQWRAKYEHDAMQRTEDLEEAKKKLAIRLQEAAEAMEVSNAKNASLERARHR
Mmu_Myo7B_gi145864471refNP_001 EESKAKGALAHAVQALRHDCDLLREQHEEESEAQAELQRLLSKANAEVAQWRSKYEADAIQRTEELEEAKKKLALRLQEAEEGVEAANAKCSSLEKAKLR
MmcMHC7 EEVKAKNALAHALQSARHDCDLLREQYEEETEAKAELQRVLSKANSEVAQWRTKYETDAIQRTEELEEAKKKLAQRLQDAEEAVEAVNAKCSSLEKTKHR
Mmu_Myo6_gi6754774refNP_034986 EEGKAKNALAHALQSSRHDCDLLREQYEEEMEAKAELQRVLSKANSEVAQWRTKYETDAIQRTEELEEAKKKLAQRLQDAEEAVEAVNAKCSSLEKTKHR
Mmu_Myo13_gi124486959refNP_001 EETKAKNALAHALQSSRHDCDLLREQYEEEQEGKAELQRALSKANSEVAQWRTKYETDAIQRTEELEEAKKKLAQRLQEAEENTEASNSKCASLEKTKQR
Mmu_Myo3_gi153792649refNP_0010 EENKAKNALAHALQSSRHDCDLLREQYEEEQEGKAELQRALSKANSEVAQWRTKYETDAIQRTEELEEAKKKLAQRLQDSEEQVEAVNAKCASLEKTKQR
MmskMHC8 EETKAKNALAHALQSSRHDCDLLREQYEEEQEGKAELQRALSKANSEVAQWRTKYETDAIQRTEELEEAKKKLAQRLQAAEEHVEAVNAKCASLEKTKQR
Mmu_Myo2_gi205830428refNP_0010 EEVKAKNALAHALQSSRHDCDLLREQYEEEQESKAELQRALSKANSEVAQWRTKYETDAIQRTEELEEAKKKLAQRLQAAEEHVEAVNAKCASLEKTKQR
Mmu_Myo4_gi67189167refNP_03498 EESKAKNALAHALQSARHDCDLLREQYEEEQEAKAELQRAMSKANSEVAQWRTKYETDAIQRTEELEEAKKKLAQRLQDAEEHVEAVNSKCASLEKTKQR
Mmu_Myo1_gi82524274refNP_10960 EEIKAKSALAHALQSSRHDCDLLREQYEEEQEAKAELQRAMSKANSEVAQWRTKYETDAIQRTEELEEAKKKLAQRLQDAEEHVEAVNAKCASLEKTKQR
Hma_21579261_gi221113859refXP_ AESKAKLENYNKLQNALAEIESLQEQLEEEAAAKQDLQNKYLRAAAEAQQWKAKYEQDGANRVEELEEAKKKLAKRVQEVEEALVAAESKAAGMEKVKNR
Asumyo4 DEARERQTLAAQAKNFQIEAEQAHNNLDEEIESKNEVMRQLSKANAEIQQWQSKFEGDGMLRADEIEEVKRKQGLKINELQEALEMANQKVLSLEKTKSR
AqMHCIIb SETSAKLDALSKLKQAQNEVQNLNETVEEEQDSKTELSKQLAAAKAEAAQWKTKFETEINPRMEELEDSKRKLQTQMKDLEEQLSTTQTKYSSLEKTKNR
Aaesmnmmy EEARERATLLGKFRNLEHDLDNLREQVEEEAEGKGDIQRQLSKANAEAQLWRSKYESEGVARAEELEEAKRKLQARLAEAEETIESLNQKCVALEKTKQR
DmeMHCC EESRERATLLGKFRNLEHDLDNLREQVEEEAEGKADLQRQLSKANAEAQVWRSKYESDGVARSEELEEAKRKLQARLAEAEETIESLNQKCIGLEKTKQR
Lpemyosin DEGRLRAKLQAEVRNLNSDIDGIRESLEEEAESKSDLQRALSRANAEVQQWRSKFESEGAARADELEDAKRKLQAKLSEAEQTADTLHSKCAGLEKAKSR
LgMHCII3 EETRIRQKLQSEVRNLTTDIDSLRDQIEEEQDTKCAMQRQLSKANSEAQSWKSKYESEGLSRMEELEEAKRKLQAKLADAEQNLEAANIKINNLEKVKCR
TaMHCb DESQARSSVSNNVKNLQAELEQTQELLDEEQESKAELQRQLTKANADAATWRNKYEIDGAQRVEELEDIKRKLTAKLTSAEELADEFQMKFSSAEKSKNR
Nve_SB1 ----------------------------------------------------------------------------------------------------

      1810      1820      1830      1840      1850      1860      1870      1880      1890      1900
....|....|....|....|....|....|....|....|....|....|....|....|....|....|....|....|....|....|....|....|
Mmu_MyoX_gi130507685refNP_0623 ----------------------------------------------------------------------------------------------------
Mmu_VIIa_gi115511010refNP_0326 ----------------------------------------------------------------------------------------------------
Mmu_VIIb_gi261823963refNP_1157 ----------------------------------------------------------------------------------------------------
Ddi_0301_gi166240253refXP_0017 ----------------------------------------------------------------------------------------------------
Ddi_1951_gi66823681refXP_64519 ----------------------------------------------------------------------------------------------------
Sce_9711_gi6324902refNP_014971 ----------------------------------------------------------------------------------------------------
Sce_9373_gi6319290refNP_009373 ----------------------------------------------------------------------------------------------------
Mmu_MyoVc_gi124486759refNP_001 ----------------------------------------------------------------------------------------------------
Mmu_MyoVa_gi115511052refNP_034 ----------------------------------------------------------------------------------------------------
Mmu_MyoVb_gi46399202refNP_9638 ----------------------------------------------------------------------------------------------------
Ddimyo AEFDLEEAVKNLEEETAKKVKAEKAMKKAETDYRSTKSELDDAKNVSSEQYVQIKRLNEELSELRSVLEEADERCNSAIKAKKTAESALESLKDEIDAAN
Sce_1888_gi6321812refNP_011888 ----------------------------------------------------------------------------------------------------
Sar_1270_SARC_01270T0Sphaerofo LQGELDDANAAVETAQSEASSALKKAKKFDAQINEQKQLAQESDARARIAEAEASRLGTDVYSLKQQIEDLNEQLMSAKKESSNLKSELEESRNSSNKDA
Mbr_4119_nullMonosigabrevicoll VESEVEDLHVEIQTLQATISSLNNKQRSHDKFLAEERDKTSVITEERDSLQSQIRELSTKVLTLKNELETVTDRLETAESAKRRLQAEIDDMIANQDDSA
Sro_4749_PTSG_04749T0Salpingoe LVSELEDATVELDHSRSQIAQLTAHQRSFDKQLNDERGRYEELAKERDALQSQNRELSTKVLTLKNELEEATDRCESTEKAKKRLQDELDDLVSSSNDAA
PpiMHCa LQGEVDDLSVDLEKEKTLVNSLQNQQKKFDATLNKERERADQLEADKEGYEKKSRDLESKVLSLQAELEDTNDKWLDSERARKQQAAELGELMESKDDAG
Aqu_8281_gi340368578refXP_0033 IQEDLDDLMVQVESQRSEILQLEKKQKKFDNQLTEVQSLAEKNAQERDEAEQRARQAETKSLSLNRELEELQEKLEDVERQRRALQSERDSLVETKDDVG
Cow_3939_CAOG_03939T0Capsaspor LQAELADVQVSLDAERNTRSNLEKKQKKFDQQFAEEKARGEEIARERDDAQRDARDSQTKILTSQKELEAAREKISELERAKKTLSAELQDLVASKDGAG
TaMHCa LQCEVDDLNVDLEKGKSTISNLEKRQRKFDQQLAEERSKSEMIANDRDTAEKTVRSNETTIISLKRELEELRDQHEDSERIRRQQQAEINDLMESKDDAG
Asumyo LQQEVEDANIELNNIKAYARDMEKKQRKFDQQLAEERANIQKISNERDTHAQESRDRETRILSLNNELEQLRSQLEESERVKRLLQMELDESVSSKDDVG
Hma_21673571_gi221130996refXP_ LQGDLDDVTVLMERERNNASQMAAKQKKFDQLLSEEKSRSQALASERDNVEKVARANETKILSLQSANEELEDKLAESERVRKSLMAELQELIDSKDGAG
Nve_JGI5820_jgiNemve1118073jgi LQNEVDDLQLNLDKERSTIANLEKRQRKFDQMMAEEKAISERYAMERDNAEREARQNETKVISLSHELEEYQDKLAESERLRKLAQTELEGVMESKDDFG
Mmu_Myo14_gi29336026refNP_0822 LQQELDDATVDLGQQKQLLSTLEKKQRKFDQLLAEEKAAVLRAVEDRERIEAEGREREARALSLTRALEEEQEAREELERQNRALRAELEALLSSKDDVG
MmsmMHC11 LQQELDDLVVDLDNQRQLVSNLEKKQKKFDQLLAEEKNISSKYADERDRAEAEAREKETKALSLARALEEALEAKEELERTNKMLKAEMEDLVSSKDDVG
Mmu_Myo9_gi114326446refNP_0718 LQQELDDLLVDLDHQRQSVSNLEKKQKKFDQLLAEEKTISAKYAEERDRAEAEAREKETKALSLARALEEAMEQKAELERLNKQFRTEMEDLMSSKDDVG
MmuMHC10 LQQELDDLTVDLDHQRQIVSNLEKKQKKFDQLLAEEKGISARYAEERDRAEAEAREKETKALSLARALEEALEAKEEFERQNKQLRADMEDLMSSKDDVG
Lpenm LSEENEDFRVELDQQIAQVNELTRKQAKFDQKLAEEKAISERLSAENEKSQREINEKSTKIMNIMNELEEEREKSEEMEKKWKKSHNELEGLVSSVDSDK
LgnmMHCII1 LQAEVEDLTMELENQRSASTAFEKKSRKFDALLAEEKANAERLAIERDNVERESREKETKILNLTRELDDLQERYDGLERIKQQQQRELDDLVSSKDDVG
Aaesmnm IQSELEDATIELETQRTKVLDLEKKQKNFDKVLAEEKAVSEQHAQERDAAEREAREKETKVLSLTRELDEAYEKIDDLETKRKALQNELDELANTQGTAD
Dme_zipper_gi62471805refNP_001 IQSELEDATIELEAQRTKVLELEKKQKNFDKILAEEKAISEQIAQERDTAEREAREKETKVLSVSRELDEAFDKIEDLENKRKTLQNELDDLANTQGTAD
PpiMHCb1 LMHELEDAAVELERTRSELSNMDKKCKKVDVTILEWKGKYDTASAAAESLTMELQKSNTECLNLKSTCEEYTVSIENYSRNSKKLEATIRDLNSEL-EGG
PpiMHCb2 ITMELEDTSVELDRVKNDLSSMDKKQRKVDQMMSEWKGKCDQETAKNEGLVREIQKISTDHMNLKNTNNEVQEELDSVPRQIKSLQSENNDLTEQLEGGG
Mmu_Myo15_gi261245016refNP_001 LQLELGDALSDLGKARSVAAALGQKQQHSDKALTSWKQKLDETQELLQASQKETRALSSEVLTFRQACEESTEAQETLKRQNQDLQEQICSLTNQVREGI
Mmu_Myo7B_gi145864471refNP_001 LQTESEDVTLELERATSAAAALDKKQRHLERALEERRRQEEEMQRELEAAQREARGLGTELFRLRHSHEEALEALETLKRENKNLQEEISDLTDQVSLSG
MmcMHC7 LQNEIEDLMVDVERSNAAAAALDKKQRNFDKILAEWKQKYEESQSELESSQKEARSLSTELFKLKNAYEESLEHLETFKRENKNLQEEISDLTEQLGSTG
Mmu_Myo6_gi6754774refNP_034986 LQNEIEDLMVDVERSNAAAAALDKKQRNFDKILAEWKQKYEESQSELESSQKEARSLSTELFKLKNAYEESLEHLETFKRENKNLQEEISDLTEQLGEGG
Mmu_Myo13_gi124486959refNP_001 LQGEVDDLMLDLERANTACATLDKKQRNFDKVLAEWKQKLDESQAELEAAQKESRSLSTEIFKMRNAYEEVVDQLETLRRENKNLQEEISDLTEQIAETG
Mmu_Myo3_gi153792649refNP_0010 LQGEVEDLMVDVERANSLAAALDKKQRNFDKVLAEWKTKCEESQAELEAALKESRSLSTELFKLKNAYEEALDQLETVKRENKNLEQEIADLTEQIAENG
MmskMHC8 LQNEVEDLMIDVERTNAACAALDKKQRNFDKVLSEWRQKYEETQAELESCQKESRTLSTELFKVKNAYEESLDHLETLRRENKNLQQEISDLTEQIAEGG
Mmu_Myo2_gi205830428refNP_0010 LQNEVEDLMLDVERTNAACAALDKKQRNFDKILAEWKQKYEETHAELEASQKEARSLGTELFKMKNAYEESLDQLETLKRENKNLQQEISDLTEQIAEGG
Mmu_Myo4_gi67189167refNP_03498 LQNEVEDLMIDVERSNAACAALDKKQRNFDKVLAEWKQKYEETQAELEASQKESRSLSTELFKVKNAYEESLDQLETLKRENKNLQQEISDLTEQIAEGG
Mmu_Myo1_gi82524274refNP_10960 LQNEVEDLMIDVERTNAACAALDKKQRNFDKILAEWKQKYEETHAELEASQKESRSLSTELFKIKNAYEESLDHLETLKRENKNLQQEISDLTEQIAEGG
Hma_21579261_gi221113859refXP_ MNEEVEDLLLDLEKAQAQASNLEKKQKKVDQQINEWKQKCEEIQAEFDKAQRDARSYSTDLLKAKAANEEANEKIEALKKENRILTADIQAISEQVNEGG
Asumyo4 LMGDLDDAQVDVERANSYASQLEKKQKGFDKVVEEWKKKSDDLSAELDAAQREARNLSTDLIKMKTSHEELLETVEGLRRENKGLCNEIKDLSDQLGEGG
AqMHCIIb VAGELEDVMLDLEKERNATAAWEKKKKVIDQQINEWKAKYEGSQQELDASKSESRLYSTELLKLKSENSDLKDDLETATKDNKRLQNDLADLEKQLTEGG
Aaesmnmmy LSTEVEDLQLEVDRATSIANAAEKKQKAFDKIIGEWKLKVDDLAAELDASQKECRNYSTELFRLKGAYEEGQEQLEAVRRENKNLADEVKDLLDQIGEGG
DmeMHCC LSTEVEDLQLEVDRANAIANAAEKKQKAFDKIIGEWKLKVDDLAAELDASQKECRNYSTELFRLKGAYEEGQEQLEAVRRENKNLADEVKDLLDQIGEGG
Lpemyosin LQGELEDLAIDVERSSAHANNLEKKQRNFDKVVSEWQHKCNDLQAELENAQKEARSYSAELFRVRAQCEEVGDTVASLRRENKNLADEIHDLTDQLGEGG
LgMHCII3 LQTENEDMMIEVERTSTTANILEKKQRSFDKTTSEWQTKFSELQLEIETVQRESRALSAELFRVKAKDVESVERMESLRRENKHLADEIRELTDQLSEGG
TaMHCb LSTEMEDLLLDLERAQNQCAAMEKKQRKVDAQTAEWRNKHDTLTIDLENAQREYREQSTELFRVKGSYQETAELVDGLRRENKQLQNELSDLSEQLNSDG
Nve_SB1 ----------------------------------------------------------------------------------------------------

      1910      1920      1930      1940      1950      1960      1970      1980      1990      2000
....|....|....|....|....|....|....|....|....|....|....|....|....|....|....|....|....|....|....|....|
Mmu_MyoX_gi130507685refNP_0623 ----------------------------------------------------------------------------------------------------
Mmu_VIIa_gi115511010refNP_0326 ----------------------------------------------------------------------------------------------------
```

```
Mmu_VIIb_gi261823963refNP_1157      ----------------------------------------------------------------------------------------------------
Ddi_0301_gi166240253refXP_0017      ----------------------------------------------------------------------------------------------------
Ddi_1951_gi66823681refXP_64519      ----------------------------------------------------------------------------------------------------
Sce_9711_gi6324902refNP_014971      ----------------------------------------------------------------------------------------------------
Sce_9373_gi6319290refNP_009373      ----------------------------------------------------------------------------------------------------
Mmu_MyoVc_gi124486759refNP_001      ----------------------------------------------------------------------------------------------------
Mmu_MyoVa_gi115511052refNP_034      ----------------------------------------------------------------------------------------------------
Mmu_MyoVb_gi46399202refNP_9638      ----------------------------------------------------------------------------------------------------
Ddimyo                              NAKAKAERKSKELEVRVAELEESLEDKSGTVNVE--FIRKKDAEIDDLRARLDRETESRIKS-DEDKKNTRKQFADLEAKVEEAQREVVTIDRLKKKLES
Sce_1888_gi6321812refNP_011888      ----------------------------------------------------------------------------------------------------
Sar_1270_SARC_01270T0Sphaerofo      GYVASLEKAKRVLEAQLEEMRTEMEELEDELQQTEDAKVRLEVSFQATKQQLERELAERDDSIEEGRRGLMRQMRDLEEQLDEERRAKSTVGSKITKLEH
Mbr_4119_nullMonosigabrevicoll      KT--ELENAKRVLTQQVEEMKQQVLELEDDNQILNEQNLRMKVDMQALEQKANAVDVERAEEDAKERKKLQRKLHELEEELESERRTRSKLAGERKRLEL
Sro_4749_PTSG_04749T0Salpingoe      QA--ELEAAKRTLQITVDEQKQQIIELEDELQLVEQAKLRLEVNMSAMEKKMKEVDQTAMEEEHKKLKQLNRQIRVLEEELENERRISSKTSAEKRKLDL
PpiMHCa                             KNVHELEKSKRALENLVQEQKVQLEELEDELQATEDAKLRLEVNMQAQKANYERELTSREEASEEKRRQILKQLREQEQEIEDERKQRTSAMTARKKLEG
Aqu_8281_gi340368578refXP_0033      KSVHELAKNKKQLEQQLEEMKQQLEEVDDELQVAEDARLRLEVNMQAAKTSYERELSSKEEQIEDTRRKLTQQIRDLESQLEEEKKQKNIAQIAKRKMEG
Cow_3939_CAOG_03939T0Capsaspor      RNVHELEKSKRALEQVVEEQKTQLEELEDELQATEDAKLRLEVNLQAMKAQLDRELNAKDEQTEDARKATLKQLRELEEELEEERRSKSNILQQKKKLEL
TaMHCa                              KNVHELEKAKQTLAAHVEDLKAQLEELEDELQLAEDGKLRLEVNLQAAKSNLDREVSQRDEQIEEARRSLLKQVRELETEVEDERSQRSQAVSAKKKLEM
Asumyo                              KSVHELEKAKRQLEVELQEQKAQIEELEDAVQIAEDARLRLDVNLQALKAEQERALAAKEQESEEKRKSLFKQIRDLESELENERRGKTGAVSQRKKMET
Hma_21673571_gi221130996refXP_      KNIHELDKAKRLLEQQLAEQKTQVEELEDELQATEDAKLRLEVNMQAQKAQFERELAAKEDSIEEGRKGLIKQLREMELELEEERKVSKAAGASKRKLES
Nve_JGI5820_jgiNemve1118073jgi      KNVHELEKAKRSLEQQLAEMKQTMEELEDELQATEDAKLRMEVNMQAMKTQFERDLAARDDQNEEKRKALLKQLRELESELDEERKLRASAVNARKKQEL
Mmu_Myo14_gi29336026refNP_0822      KNVHELERARKAAEQAASDLRTQVTELEDELTAAEDAKLRLEVTVQALKAQHERDLQGRDDAGEERRRQLAKQLRDAEVERDEERKQRALAMAARKKLEL
MmsmMHC11                           KNVHELEKSKRALETQMEEMKTQLEESEDDVQATEDAKLRLEVNMQALKGQFERDLQARDEQNEEKRRQLQRQLHEYETELEDERKQRALAAAAKKKLEG
Mmu_Myo9_gi114326446refNP_0718      KSVHELEKSKRALEQQVEEMKTQLEELEDELQATEDAKLRLEVNLQAMKAQFERDLQGRDEQSEEKKKQLVRQVREMEAELEDERKQRSMAMAARKKLEM
MmuMHC10                            KNVHELEKSKRALEQQVEEMRTQLEELEDELQATEDAKLRLEVNMQAMKAQFERDLQTRDEQNEEKKRLLLKQVRELEAELEDERKQRALAVASKKKMEI
Lpenm                               RSVLDLERQKKQLESQVQEQLGLILESEEDLQKMESSKLRNEVNLQAIISKLQKELSGRDENADEIKRGLQRQLKELEAEVEDARTQKTAAVHGKKKLEH
LgnmMHCII1                          KNVHELEKAKRTFQQQVEEQRQQIEELEDELQLAEDAKLRLEVNMQALKAQYERDIQGKEDQVEEGKKSLLRQLREMEAELEEERKQRGAAVNARNKLQG
Aaesmnm                             KNVHELEKAKRALESQLAELKAQNEELEDDLQLTEDAKLRLEVNMQALRAQFERDVQAKEEQSEEKRRGLVKALRDMEAELDEERKQRAAAVAAKKKLEG
Dme_zipper_gi62471805refNP_001      KNVHELEKAKRALESQLAELKAQNEELEDDLQLTEDAKLRLEVNMQALRSQFERDLLAKEEGAEEKRRGLVKQLRDLETELDEERKQRTAAVASKKKLEG
PpiMHCb1                            RGNHELEKIRRKLESENEELRQQMDELEIAVGSSESKCTKLTLEIANIRNSYERQLADVDEEGDKARKGLLRQIEQLRMQLEDQEKAKGDLAKVKKNLEM
PpiMHCb2                            KATHEIEKLRRKLETDNEELRGQLEETEGLLTMEENKTSKLQLELSGVKMEMERRIGEKEEDSDKDRKNLLRQIDTLHQQLEDEAKVKNDLAKARKMAEG
Mmu_Myo15_gi261245016refNP_001      KNLAEVEKAKKLIEQEKTEVQVRLEETEGALERNESKILRFQLELSEAKAELERKLSEKEEEAERLREKHQQAMGSLQSNLDLEASSRIEATRLRKKMEG
Mmu_Myo7B_gi145864471refNP_001      KSIQELEKAKKALEGEKSELQAALEEAEGALELEETKTLRIQLELSQVKAEVDRKLAEKDEECTNLRRNHQRAVESLQASLDAETRARNEALRLKKKMEG
MmcMHC7                             KSIHELEKIRKQLEAEKLELQSALEEAEASLEHEEGKILRAQLEFNQIKAEIERKLAEKDEEMEQAKRNHLRMVDSLQTSLDAETRSRNEALRVKKKMEG
Mmu_Myo6_gi6754774refNP_034986      KNVHELEKIRKQLEVEKLELQSALEEAEASLEHEEGKILRAQLEFNQIKAEIERKLAEKDEEMEQAKRNHLRMVDSLQTSLDAETRSRNEALRVKKKMEG
Mmu_Myo13_gi124486959refNP_001      KNLQEVEKTKKQVEQEKSDLQAALEEVEGSLEHEESKILRVQLELSQVKSELDRKVTEKDEEIEQIKRNSQRAVEAMQSVLDAEIRSRNDALRLKKKMEG
Mmu_Myo3_gi153792649refNP_0010      KSIHELEKSRKQMELEKADIQMALEEAEAALEHEEAKILRIQLELTQVKSEIDRKIAEKDEEIEQLKRNYQRTVETMQGALDAEVRSRNEAIRLKKKMEG
MmskMHC8                            KHIHELEKIKKQVEQEKCEIQAALEEAEASLEHEEGKILRIQLELNQVKSEIDRKIAEKDEEIDQLKRNHVRVVETMQSTLDAEIRSRNDALRVKKKMEG
Mmu_Myo2_gi205830428refNP_0010      KRIHELEKIKKQVEQEKCELQAALEEAEASLEHEEGKILRIQLELNQVKSEIDRKIAEKDEEIDQLKRNHIRVVESMQSTLDAEIRSRNDAIRIKKKMEG
Mmu_Myo4_gi67189167refNP_03498      KHIHELEKIKKQIDQEKSELQASLEEAEASLEHEEGKILRIQLELNQVKSEIDRKIAEKDEEIDQLKRNHLRVVESMQSTLDAEIRSRNDALRIKKKMEG
Mmu_Myo1_gi82524274refNP_10960      KRIHELEKIKKQIEQEKSELQAALEEAEASLEHEEGKILRIQLELNQVKSEIDRKIAEKDEEIDQLKRNHIRVVESMQSTLDAEIRSRNDAIRLKKKMEG
Hma_21579261_gi221113859refXP_      KNSLEIDKLRRKLGLENEELQVALEEAELALEQEEGKLLKAQLELTQLKQATEKKFAERDEELENMRKNHQRQIESLQNTIDNEQRNRNEQAKQRKQYDA
Asumyo4                             RSAHETQKIIRRLEVEKEELQHGLDEAEAALEAEESKVMRAQVEVAQIRSEVEKRIQEKEEEFDSTRRNHQRALESMQASLEAESKGKTELLRVKKKLEA
AqMHCIIb                            ASVHDLEKAKKKLQQELEEINLMMEENEGQLEIEQNTVLRLQLEISQLKQENERKLSEKDDEMDSLRKNHQRQLDALQQSLEEENKAKNDAVRQKKAAEE
Aaesmnmmy                           RNIHEIEKSRKRLEAEKDELQAALEEAEAALEQEENKVLRAQLELSQVRQEIDRRIQEKEEEFENTRKNHQRALDSMQASLEAEAKGKAEALRMKKKLEA
DmeMHCC                             RNIHEIEKARKRLEAEKDELQAALEEAEAALEQEENKVLRAQLELSQVRQEIDRRIQEKEEEFENTRKNHQRALDSMQASLEAEAKGKAEALRMKKKLEA
Lpemyosin                           RNTHELEKARKHLALEKEELQAALEEAEGALEQEEAKVMRATLEISQIRQEIDRRLQEKEEEFDNTRRNHQRAIESMQASLEAEAKGKAEALRIKKKLEG
LgMHCII3                            RSVHEIEKIRRRVELEKEELQSALEEAHATLEQEEAKVMRLQLEISQVRQEVDRRVHEKDEEFENTRRNHQRAIESMQASLEIEIKNKTEAMRIRKKLEH
TaMHCb                              RNAAEIEKVRRKLEVEKEEIALLLEEAEASLEQEENKVLRMQLEQSQLKQEFERRIHEKEEEMEGLRKNHQRQLEAVNNSLGSESKSKSEQIRLKKKLEA
Nve_SB1                             ----------------------------------------------------------------------------------------------------

                                          2010      2020      2030      2040      2050      2060      2070      2080      2090      2100
                                    ....|....|....|....|....|....|....|....|....|....|....|....|....|....|....|....|....|....|....|....|
Mmu_MyoX_gi130507685refNP_0623      ----------------------------------------------------------------------------------------------------
Mmu_VIIa_gi115511010refNP_0326      ----------------------------------------------------------------------------------------------------
Mmu_VIIb_gi261823963refNP_1157      ----------------------------------------------------------------------------------------------------
Ddi_0301_gi166240253refXP_0017      ----------------------------------------------------------------------------------------------------
Ddi_1951_gi66823681refXP_64519      ----------------------------------------------------------------------------------------------------
Sce_9711_gi6324902refNP_014971      ----------------------------------------------------------------------------------------------------
Sce_9373_gi6319290refNP_009373      ----------------------------------------------------------------------------------------------------
Mmu_MyoVc_gi124486759refNP_001      ----------------------------------------------------------------------------------------------------
Mmu_MyoVa_gi115511052refNP_034      ----------------------------------------------------------------------------------------------------
Mmu_MyoVb_gi46399202refNP_9638      ----------------------------------------------------------------------------------------------------
Ddimyo                              DIIDLSTQLDTETKSRIKIEKSKKKLEQTLAERRAAEEGSSKAADEEIRKQVWQEVDELRAQLDSERAALNASEKKIKSLVAEVDEVKEQLEDEILAKDK
Sce_1888_gi6321812refNP_011888      ----------------------------------------------------------------------------------------------------
Sar_1270_SARC_01270T0Sphaerofo      DLIEAQTSLDMAEKGRDQAAREAKKAVQFV-------------------RDLQQENDMSYSQMQDANNQLREVERKAQKYHSENAMLAVETERN------
Mbr_4119_nullMonosigabrevicoll      EVSEAQQLA-------------------------------------------------------------------------------------------
Sro_4749_PTSG_04749T0Salpingoe      EMAD------------------------------------------------------------------------------------------------
PpiMHCa                             DYQELEMLLEGATHAKEDLLKQNKKLQAAL-------------------KDFQHDLDEARHRADEANENAKSSERRAKDYESQLIGLQEDLTAS------
Aqu_8281_gi340368578refXP_0033      EISEWEARAEADAKAKEEAVKQYRKVQNQL-------------------KDAQLSAEEARAAQDEMISRVKELEKKVRGLEGDLSQKQEELQNA------
Cow_3939_CAOG_03939T0Capsaspor      DLKDSEGHVDEANKNKEEAVRQAKKAQQQV-------------------KDMQREIDESRQTREEIANAQRDAERKAAALAAEVSRLQADLDQS------
TaMHCa                              DVKELESQVELANKSRDDDVRQNKKLQGQV-------------------KEMQNELDELRVSREELYDQSRDLDRKFRTAEGDLRQAQEELANS------
Asumyo                              QMGELTQQLEVANRLKDDYNRQLKKYQQMI-------------------KELQHDSEESRQAKEELAASLREIERKLRSAESENIRLMEANETL------
Hma_21673571_gi221130996refXP_      DVKELTAQLDQANRLKEDSQKQLKKYQVHL-------------------KDVQRDLDEAKAAREELSAQVKDNEKKLKGLESDFVQMQEDLSTA------
Nve_JGI5820_jgiNemve1118073jgi      DLRDLEDQLEAANKVKEDGLRQLKKYQQQL-------------------KDIQRDLDDARASRDEIAEHAKENEHKLKQLEADFIQMQEDLSAA------
Mmu_Myo14_gi29336026refNP_0822      ELEELKAQTSAAGQGKEEAVKQLKKMQVQM-------------------KELWREVEETRSSRDEMFTLSRENEKKLKGLEAEVLRLQEELAAS------
MmsmMHC11                           DLKDLELQADSAIKGREEAIKQLRKLQAQM-------------------KDFQRELDDARASRDEIFATSKENEKKAKSLEADLMQLQEDLAAA------
Mmu_Myo9_gi114326446refNP_0718      DLKDLEAHIDTANKNREEAIKQLRKLQAQM-------------------KDCMRELDDTRASREEILAQAKENEKKLKSMEAEMIQLQEELAAA------
MmuMHC10                            DLKDLEAQIEAANKARDEVIKQLRKLQAQM-------------------KDYQRELEEARASRDEIFAQSKESEKKLKSLEAEILQLQEELASS------
Lpenm                               DLNQLSEQLIAVTKQKEDAVKQARKANSQM-------------------KEYQRETEEARAAQHDANTAFKDMKKKVKSLEDELVVMQEDVAAA------
LgnmMHCII1                          DLNDYEQQVQMANKVKDDAVKQYKRMVTQM-------------------KEVTREVDEIRISRDEMSQAQKDNEKRVKTLEAEVLRLSEDLAAS------
Aaesmnm                             DLKDIEATLEMNNKVKEDALKQAKKLQAQV-------------------KDAIRDAEEAKAAKEELAAVSKEAERKAKALEAEVMQLSEDFASS------
Dme_zipper_gi62471805refNP_001      DLKEIETTMEMHNKVKEDALKHAKKLQAQV-------------------KDALRDAEEAKAAKEELQALSKEAERKVKALEAEVLQLTEDLASS------
PpiMHCb1                            EIGNLNEDLGNADKTIAALNANIGKHQKTV-------------------QEMTMALDEAHRALDDHKTALLRSDKRASELTHIKEELEGNLDGS------
PpiMHCb2                            EVGGLHEDLDNAEKTIAALNNNLGKQQKGI-------------------QELQLALEDANKNVENLKAALLKSDKKATTLATQKTDIEGSLERA------
Mmu_Myo15_gi261245016refNP_001      DLKEMEIQLCAANRQVSQMTRALGQLQGQM-------------------KDLHQQLDDSIYQNKDLKEQVALAEQRTVLLQSELEELRTLQEQT------
Mmu_Myo7B_gi145864471refNP_001      DLNDLELQLGHATRQAMEAQAATRLLQAQL-------------------KEEQAGRDEEQRLAAELREQGQALERRAALLAAELEELRAALEQG------
MmcMHC7                             DLNEMEIQLSHANRMAAEAQKQVKSLQSLL-------------------KDTQIQLDDAVRANDDLKENIAIVERRNNLLQAELEELRAVVEQT------
Mmu_Myo6_gi6754774refNP_034986      DLNEMEIQLSQANRIASEAQKHLKNSQAHL-------------------KDTQLQLDDAVHANDDLKENIAIVERRNNLLQAELEELRAVVEQT------
Mmu_Myo13_gi124486959refNP_001      DLNEMEIQLSHANRQVAETQKHLRTVQGQL-------------------KDSQLHLDDAQRSNEDLKEQLAIVERRNGLLQEELEEMKVALEQT------
Mmu_Myo3_gi153792649refNP_0010      DLNEIEIQLSHANRQAAETIKHLRSVQGQL-------------------KDTQLHLDDALRGQEDLKEQLAIVERRANLLQAEVEELRATLEQT------
MmskMHC8                            DLNEMEIQLNHANRLAAESLRNYRNTQGIL-------------------KDTQLHLDDALRGQEDLKEQLAIVERRANLLQAEIEELRATLEQT------
Mmu_Myo2_gi205830428refNP_0010      DLNEMEIQLNHSNRMAAEALRNYRNTQGIL-------------------KDTQLHLDDALRGQEDLKEQLAMVERRANLLQAEIEELRATLEQT------
Mmu_Myo4_gi67189167refNP_03498      DLNEMEIQLNHANRQAAEAIRNLRNTQGML-------------------KDTQLHLDDALRGQDDLKEQLAMVERRANLMQAEIEELRASLEQT------
Mmu_Myo1_gi82524274refNP_10960      DLNEMEIQLNHSNRMAAEALRNYRNTQGIL-------------------KDTQLHLDDALRGQEDLKEQLAMVERRANLLQAEIEELRATLEQT------
Hma_21579261_gi221113859refXP_      EMNELESQVETANRQSSEYQKQIKKMAAQI-------------------KELQTMIDDESNNRDNLRDTANRAERRANELAVQLDEAKTALEQA------
Asumyo4                             DINELEIALDHANKANADAQKNLRRYNEQI-------------------REIQLQIEEEQRQRDEMKEQYYGAEKRAALLQSEKEELAAACEQA------
AqMHCIIb                            QLDDLQANLESAQKSSTDLGKLVKKLQAQV-------------------KDLSGRIDEETRQKDEQHQLAAKAEKRANDLTVEVEELRSSTEQL------
Aaesmnmmy                           DINELEIALDHANKANAEAQKNIKRYQQQL-------------------KDLQGALEEEQRARDDAREQLGISERRANALQNELEESRTLLEQA------
DmeMHCC                             DINELEIALDHANKANAEAQKNIKRYQQQL-------------------KDIQTALEEEQRARDDAREQLGISERRANALQNELEESRTLLEQA------
Lpemyosin                           DINELEIALDATNRGKAELEKNVKKYQGQI-------------------RELQSQVEEEQAQRDEAKEHYQMAERRCAAINGELEELRTILEQA------
LgMHCII3                            DINELEIALDAANRNRVEAEKTTKKFQLQI-------------------TEIQTMIEDERRQKEEAREAYNMAERRCGVISCEVEELRTSLEQS------
TaMHCb                              EITELELTLEAANKSNADLNRSVKKYQSQV-------------------KELQSMIDDEQRVHEDYRDKVQKAERRANDVLGELEDLRASLEQS------
Nve_SB1                             ----------------------------------------------------------------------------------------------------
```

```
                                             2110      2120      2130      2140      2150      2160      2170      2180      2190      2200
                                    ....|....|....|....|....|....|....|....|....|....|....|....|....|....|....|....|....|....|....|....|
Mmu_MyoX_gi130507685refNP_0623      ----------------------------------------------------------------------------------------------------
Mmu_VIIa_gi115511010refNP_0326      ----------------------------------------------------------------------------------------------------
Mmu_VIIb_gi261823963refNP_1157      ----------------------------------------------------------------------------------------------------
Ddi_0301_gi166240253refXP_0017      ----------------------------------------------------------------------------------------------------
Ddi_1951_gi66823681refXP_64519      ----------------------------------------------------------------------------------------------------
Sce_9711_gi6324902refNP_014971      ----------------------------------------------------------------------------------------------------
Sce_9373_gi6319290refNP_009373      ----------------------------------------------------------------------------------------------------
Mmu_MyoVc_gi124486759refNP_001      ----------------------------------------------------------------------------------------------------
Mmu_MyoVa_gi115511052refNP_034      ----------------------------------------------------------------------------------------------------
Mmu_MyoVb_gi46399202refNP_9638      ----------------------------------------------------------------------------------------------------
Ddimyo                              LVKAKRALEVELEEVRDQLEEEEDSRSELEDSKRRLTTEVEDIKKKYDAEVEQNTKLDEAKKKLTDDVDTLKKQLEDEKKKLNESERAKKRLESENEDFL
Sce_1888_gi6321812refNP_011888      ----------------------------------------------------------------------------------------------------
Sar_1270_SARC_01270T0Sphaerofo      -EKARRAAELARDEAVTELETLRRDGTGSSETTRRLQADLEAKEEELEELQTELDIATDRARRAGLEKSEAECALSQEKDNFAKVEQKLQTLERANAELR
Mbr_4119_nullMonosigabrevicoll      ----------------------------------------------------------------------------------------------------
Sro_4749_PTSG_04749T0Salpingoe      ----------------------------------------------------------------------------------------------------
PpiMHCa                             -ERARRSAQQERDDLMEEMNSTALGKSSMSEENKKLLHRIAQLEDDLEEEQNDAQKHMERSKRLQQTTDELKEELNIEKATSNRMEADRLTFERQTRELQ
Aqu_8281_gi340368578refXP_0033      -DRARRSAESDRDEAQEELSTVSSKINGLSEEKRRIETRLATLEEDFEEEQLNSEAATEKARKAQQTADSLSQEISSLQSNYNQSENNRLTLEKQVKELR
Cow_3939_CAOG_03939T0Capsaspor      -ERARKAAETERDELHDEVSGGANSRSSLAEAKRRLENEIQRLQAELEDETTNAEAATDKARKLQINLDALQLEFASEQSNHRKAEEARAALDRQSKDLK
TaMHCa                              -ERARRNAEAERDELIEDSASASSLRQSWTDEKKRLESRISELEDELEDEQTENSSQQDKYRKMQMQMEQLNSDLAVEKSVSQKLEAGKTQLERQNKDLQ
Asumyo                              -MNQKRQLEADRDELEE---LHGRGGGMNAEEKRRLESKIAALEEEVEEEQSNTELALDKQRKAQLQVEQLTTELSMERSLTQKLDAEKQVLERTNRELK
Hma_21673571_gi221130996refXP_      -ERARRTIEAERDELLEELNNNTSAKTAVAEERKRWEAQIAALEEELEEERTQTELMQDKVSRGNLQIDQMSAELAEERAALQSLDNSRMTLERANKELQ
Nve_JGI5820_jgiNemve1118073jgi      -ERSRKVLERERDELAEELATLSGQRGATAEEKRKYEARIAALEEEVEEERTQSELLQEKAKRAMMQMEQMQADLNAERANAQNNESARASLEKQNKDLK
Mmu_Myo14_gi29336026refNP_0822      -DRARRQAQQDRDEMAEEVASGNLSKAATLEEKRQLEGRLSQLEEELEEEQNNSELLKDHYRKLVLQVESLTTELSAERSFSAKAESGRQQLERQIQELR
MmsmMHC11                           -ERARKQADLEKEELAEELASSLSGRNTLQDEKRRLEARIAQLEEELEEEQGNMEAMSDRVRKATLQAEQLSNELATERSTAQKNESARQQLERQNKELR
Mmu_Myo9_gi114326446refNP_0718      -ERAKRQAQQERDELADEIANSSGKGALALEEKRRLEARIAQLEEELEEEQGNTELINDRLKKANLQIDQINTDLNLERSHAQKNENARQQLERQNKELK
MmuMHC10                            -ERARRHAEQERDELADEIANSASGKSALLDEKRRLEARIAQLEEELEEEQSNMELLNDRFRKTTLQVDTLNTELAAERSAAQKSDNARQQLERQNKELK
Lpenm                               -ERARKNAETERDELQEEIANGTVSKSSLNEEKRRLELRISELEDLLDEEQNNSEILIDKNKKVNAHYEQLLTELNAEKSTSQTLENQRLSLERQNKDLK
LgnmMHCII1                          -ERARRNAESERDELQEEIGGSATSKNSLLDEKRRLEARISELEDDVDDERNNCELMAEKARKAQLQVDQIMTDLTSERSVSQKLENQRMQLERQNKEMR
Aaesmnm                             -ERARRAAEAERDELLEEINSNSNKGSLMIDEKRRLEARIATLEEELEEEQSNAELLVDRNRKAQITIEQITTELQSEKSNSQKFENVKTGLERQNKELK
Dme_zipper_gi62471805refNP_001      -ERARRAAETERDELAEEIANNANKGSLMIDEKRRLEARIATLEEELEEEQSNSEVLLDRSRKAQLQIEQLTTELANEKSNSQKNENGRALLERQNKELK
PpiMHCb1                            -DRARRGAESEKSEMAAHISDLERQISQLNAAKRKAEVDVAGLRESLEEFEAQAMSAEEKVKRHADMCSKLQSDLTREADARSGLERVNSKLSNDIFALN
PpiMHCb2                            -ERQRRTLENEKGEFTSLVSTLETQKASAESGRKKLDAECHQLRDFVEDLEGEKRDVEILLQRANDALNRCQSELNLEQESKGNQERACQTLTRRIVELE
Mmu_Myo15_gi261245016refNP_001      -ERGRKLAEKELLEATERINLFHTQNTSLLSQKKKLEADVAQVQKEAGEMLQACQKAEEKAKKTAAEAANMSEELKKEQDTNAHLERMRKNMEQTIKDLQ
Mmu_Myo7B_gi145864471refNP_001      -ERSRRLAEQELLEATERLNLLHSQNTGLLNQKKKLEVDLAQLSGEVEEAAQERREAEEKAKKAITDAAMMAEELKKEQDTSAHLERMKKTLEQTVRELQ
MmcMHC7                             -ERSRKLAEQELIETSERVQLLHSQNTSLINQKKKMDADLSQLQTEVEEAVQECRNAEEKAKKAITDAAMMAEELKKEQDTSAHLERMKKNMEQTIKDLQ
Mmu_Myo6_gi6754774refNP_034986      -ERSRKLAEQELIETSERVQLLHSQNTSLINQKKKMESDLTQLQTEVEEAVQECRNAEEKAKKAITDAAMMAEELKKEQDTSAHLERMKKNMEQTIKDLQ
Mmu_Myo13_gi124486959refNP_001      -ERTRRLSEQELLDSSDRVQLLHSQNTSLINTKKKLEADLAQCQAEVENSIQESRNAEEKAKKAITDAAMMAEELKKEQDTSAHLERMKKNLEQTVKDLQ
Mmu_Myo3_gi153792649refNP_0010      -ERARKLAEQELLDSNERVQLLHTQNTSLIHTKKKLETDLTQLQSEVEDACRDARNAEEKAKKAITDAAMMAEELKKEQDTSAHLERMKKNLEQTVKDLQ
MmskMHC8                            -ERSRKIAEQELLDASERVQLLHTQNTSLINTKKKLENDVSQLQSEVEEVIQESRNAEEKAKKAITDAAMMAEELKKEQDTSAHLERMKKNMEQTVKDLQ
Mmu_Myo2_gi205830428refNP_0010      -ERSRKIAEQELLDASERVQLLHTQNTSLINTKKKLETDISQIQGEMEDIVQEARNAEEKAKKAITDAAMMAEELKKEQDTSAHLERMKKNMEQTVKDLQ
Mmu_Myo4_gi67189167refNP_03498      -ERSRRVAEQELLDASERVQLLHTQNTSLINTKKKLETDISQIQGEMEDIVQEARNAEEKAKKAITDAAMMAEELKKEQDTSAHLERMKKNMEQTVKDLQ
Mmu_Myo1_gi82524274refNP_10960      -ERSRKIAEQELLDASERVQLLHTQNTSLINTKKKLETDISQIQGEMEDIVQEARNAEEKAKKAITDAAMMAEELKKEQDTSAHLERMKKNLEQTVKDLQ
Hma_21579261_gi221113859refXP_      -VRARKLAENDKSENSDRIAELQLLYNNMSNAKRKAESDYHALQEELEELDNEVKASEEKAAKATAEVARLSNELNQAQEAASTSEKARAILSKTILELQ
Asumyo4                             -ERARKQAELDAADGRERANELIAQTSSLNATKRKLEGELQAIHADLDETLNEFRTSEENCKKATADVERLADEVRQEQEHAAHIDRLRQGLEAQIRELQ
AqMHCIIb                            -QRQFKSADAERVELQDRLSELQSSKESLQTSKRKAEQQLSSLQEEHEELETESRENAEKLRKAMEQVSRLQTENMSQKDELNVAQKAKSSLESQLKEMT
Aaesmnmmy                           -DRGRRQAEQELSDAHEQLNEVSGQNASIGAAKRKLESELQTLHSDLDELLNEAKNSEEKAKKAMVDAARLADELRAEQDHAQTQEKLRKALEQQIKELQ
DmeMHCC                             -DRGRRQAEQELADAHEQLNEVSAQNASISAAKRKLESELQTLHSDLDELLNEAKNSEEKAKKAMVDAARLADELRAEQDHAQTQEKLRKALEQQIKELQ
Lpemyosin                           -ERARKAAENELADASDRVNELQAQVSTVGSQKRKLEGDVTAMQSDLDELNNELKDADERAKHAMADATRLADELRQEQDHGLSVEKMRKSLESQVKELQ
LgMHCII3                            -ERSRKTVEVEVTDIHIRISEMTAQFNSINSQKRKLECDIAAMTADIDELHTELRSSEDKCKKAICDVARLTEELRSEQEHCMHIEKIRKSLEATTKELQ
TaMHCb                              -ERVRKQLEVDQIDLQERVQDLTTSNSAALATRKKLETEIESLHEEIEELEGEAKSAEDKYKRANEQIARLTSEIATEHD-------------EQMKEME
Nve_SB1                             ----------------------------------------------------------------------------------------------------

                                             2210      2220      2230      2240      2250      2260      2270      2280      2290      2300
                                    ....|....|....|....|....|....|....|....|....|....|....|....|....|....|....|....|....|....|....|....|
Mmu_MyoX_gi130507685refNP_0623      ----------------------------------------------------------------------------------------------------
Mmu_VIIa_gi115511010refNP_0326      ----------------------------------------------------------------------------------------------------
Mmu_VIIb_gi261823963refNP_1157      ----------------------------------------------------------------------------------------------------
Ddi_0301_gi166240253refXP_0017      ----------------------------------------------------------------------------------------------------
Ddi_1951_gi66823681refXP_64519      ----------------------------------------------------------------------------------------------------
Sce_9711_gi6324902refNP_014971      ----------------------------------------------------------------------------------------------------
Sce_9373_gi6319290refNP_009373      ----------------------------------------------------------------------------------------------------
Mmu_MyoVc_gi124486759refNP_001      ----------------------------------------------------------------------------------------------------
Mmu_MyoVa_gi115511052refNP_034      ----------------------------------------------------------------------------------------------------
Mmu_MyoVb_gi46399202refNP_9638      ----------------------------------------------------------------------------------------------------
Ddimyo                              AKLDAEVKNRSRAEKDRKKYEKDLKDTKYKLNDEAATKTQTEIGAAKLEDQIDELRSKLEQEQAKATQADKSKKTLEGEIDNLRAQIEDEGKIKMRLEKE
Sce_1888_gi6321812refNP_011888      ----------------------------------------------------------------------------------------------------
Sar_1270_SARC_01270T0Sphaerofo      DEVE---------------------------NAEREGDRKYKSKVVPLQTRLASLQTELEGESRKAAEAANKYKRSDRKLKETMRLLEDAEKAAIEHKSN
Mbr_4119_nullMonosigabrevicoll      -----------------------------------------------------------EEERLRRDKDNSSVRKYEKRIQMLMQERDEAVHLAELDTAA
Sro_4749_PTSG_04749T0Salpingoe      ------------------------------------------------------LKERLEEEQRGRTRDSRAVKKLEKRIALLYEEREEDSKLAESSSQE
PpiMHCa                             AKLE---------------------------EVESVVKAKQGAAIASLQNKIASLEEQLDNESKEKQNLSKNSRRSDRKIKELTLQIEDERRQCEQYKDQ
Aqu_8281_gi340368578refXP_0033      DRLE---------------------------EAESAGGRKMKAQVAAMESRVAALEEERDMALKDNSQIKRSLRRQEKKLKEYAGAIEDERKQSEQYKQQ
Cow_3939_CAOG_03939T0Capsaspor      TRLD---------------------------ELEAATRGKSKTQIAQLEQRVKDLEAQVEEENRQKQELQRVNKKLDRKAKDAVAQIDEERRLAESNKAE
TaMHCa                              AKVS---------------------------ELETSSRAKSKALTQTWESRINNLEEQLESEARERTSLQKNNRRAEKRLKELTIQVEEERRHADEYKEQ
Asumyo                              SKIA---------------------------ELETSTQSRSKAQIAALEAKIQYLEDQYNAEMQERTNAARQCRRMEKRLSDAILQLEDERRNNEQHKEH
Hma_21673571_gi221130996refXP_      NKLA---------------------------DLESSLRSRTKNTVATLEAKISNLEAQLDQEAKEKQNITKLLRRTEKRAKELQVLVDEERGHTESYKQQ
Nve_JGI5820_jgiNemve1118073jgi      QKLA---------------------------DLESSTRSRHKMAIASLESKLTNMQEQLDLETKERALQSKANRKLERKIKELMLQNEEERRHSDQYREQ
Mmu_Myo14_gi29336026refNP_0822      ARLG---------------------------EEDAGARARQKMLIAALESKLAQAEEQLEQESRERILSGKLVRRAEKRLKEVVLQVDEERRVADQVRDQ
MmsmMHC11                           SKLQ---------------------------EVEGAVKAKLKSTVAALEAKIAQLEEQVEQEAREKQAATKSLKQKDKKLKEVLLQVEDERKMAEQYKEQ
Mmu_Myo9_gi114326446refNP_0718      AKLQ---------------------------EMESAVKSKYKASIAALEAKIAQLEEQLDNETKERQAASKQVRRTEKKLKDVLLQVEDERRNAEQFKDQ
MmuMHC10                            AKLQ---------------------------ELEGAVKSKFKATISALEAKIGQLEEQLEQEAKERAAANKLVRRTEKKLKEIFMQVEDERRHADQYKEQ
Lpenm                               AKVQ---------------------------ELENQAKIKQKTMIAALEGKIANLEDQLDQDNKEKNTAMRVNRKLDKKIKELMLQVEEERRHADQYKEQ
LgnmMHCII1                          EKLQ---------------------------ELEGQNRARIKATIAALESKVLNLEEQLDLEAKDRATLSRNNRKLEKRIKELILQGEDERRHADQYKEQ
Aaesmnm                             AKLS---------------------------ELETAQRTKVKAAMASLEAKIANLEEQLENETKERLGGQKANRKLEKKIKELTMNIEDERRHADQYKEQ
Dme_zipper_gi62471805refNP_001      AKLA---------------------------EIETAQRTKVKATIATLEAKIANLEEQLENEGKERLLQQKANRKMDKKIKELTMNIEDERRHVDQHKEQ
PpiMHCb1                            ARIE---------------------------EVEVSARNSAKNKVSQLEIRIRQLENDCSAEAKGRADAQHALKNLDRVIKEKEFNADEDMKTVQRLQGQ
PpiMHCb2                            GQLE---------------------------DAETAVRSAGKGKIQVMETKIRGLETDIDGEMKRRVDAEKALKKAERAIKENAFNAEEDHKTIQRTQGT
Mmu_Myo15_gi261245016refNP_001      KRLD---------------------------EAEQTAVLGSKKQIQKLESRVRDLEGELESEVRRSAEAQREARRLERGIKELTYQAEEDKKNLSRMQAL
Mmu_Myo7B_gi145864471refNP_001      ARLE---------------------------EAEQAALRGGKKQVQKLEAKVRELEAELDAEQKKHAEALKGVRKHERRVKELVYQTEEDRKNLARMQDL
MmcMHC7                             HRLD---------------------------EAEQIALKGGKKQLQKLEARVRELENELEAEQKRNAESVKGMRKSERRIKELTYQTEEDRKNLLRLQDL
Mmu_Myo6_gi6754774refNP_034986      HRLD---------------------------EAEQIALKGGKKQLQKLEARVRELENELEAEQKRNAESVKGMRKSERRIKELTYQTEEDKKNLMRLQDL
Mmu_Myo13_gi124486959refNP_001      HRLD---------------------------EAEQLALKGGKKQIQKLEARVRELESELDAEQKRGAEALKGAHKYERKVKEMTYQAEEDRKNILRLQDL
Mmu_Myo3_gi153792649refNP_0010      HRLD---------------------------EAEQLALKGGKKQIQKLETRIRELEFELEGEQKRNTESVKGLRKYERRVKELTYQSEEDRKNVLRLQDL
MmskMHC8                            HRLD---------------------------EAEQLALKGGKKQIQKLEARVRELEGEVENEQKRNAEAVKGLRKHERRVKELTYQTEEDRKNVLRLQDL
Mmu_Myo2_gi205830428refNP_0010      LRLD---------------------------EAEQLALKGGKKQIQKLEARVRELEGEVESEQKRNAEAVKGLRKHERRVKELTYQTEEDRKNILRLQDL
Mmu_Myo4_gi67189167refNP_03498      HRLD---------------------------EAEQLALKGGKKQIQKLEARVRELENEVENEQKRNIEAVKGLRKHERRVKELTYQTEEDRKNVLRLQDL
Mmu_Myo1_gi82524274refNP_10960      HRLD---------------------------EAEQLALKGGKKQIQKLEARVRELEGEVENEQKRNVEAIKGLRKHERRVKELTYQTEEDRKNVLRLQDL
Hma_21579261_gi221113859refXP_      ARLD---------------------------EAESQGAKGYKTQIKKLETRISELESELDTEVRRSAEVVKNARKNEKKVKELLFQIDDEKKAVDIAVDN
Asumyo4                             VQLD---------------------------EAEQAALKGGKKVIAKLEQRAHELEGELDGEQRHYQESIKNVAKAERQVRELQFQVDEDKKNFERMQDL
AqMHCIIb                            TKLE---------------------------EEVSVAAKSAKREAAKLQQRVTDLEMELETETKGRSDAQRNLKRSDRRYKEAVSQVEEEKANAQRLQDQ
Aaesmnmmy                           VRLD---------------------------DAETNALKGGKKAIQKLEQRVREIETELDSEQRRHADAQKNLRKSERRIKELTFQSEEDRKNHERMQDL
```

```
DmeMHCC                        VRLD---------------------------EAEANALKGGKKAIQKLEQRVRELENELDGEQRRHADAQKNLRKSERRVKELSFQSEEDRKNHERMQDL
Lpemyosin                      VRLD---------------------------ESEAAALKGGKKMIQKLESRVRELEAELDSEQRRHAETQKSMRKVDRRVKELSFQQEEDRKNYERMQEL
LgMHCII3                       IRID---------------------------EAEAQGLRGGKRVVQKLEVKIRELQSELETEQMRHSETDKNMRKIERRLKEVLFQADEDKRSQDRLQAL
TaMHCb                         AKLN---------------------------DSELAGGKASKRHVTKLEARIRELEADLEAETKQSQEATKQLRKNERRLKELQFSAEEDRKNLERTLAA
Nve_SB1                        ----------------------------------------------------------------------------------------------------

                                     2310      2320      2330      2340      2350      2360      2370      2380      2390      2400
                               ....|....|....|....|....|....|....|....|....|....|....|....|....|....|....|....|....|....|....|....|
Mmu_MyoX_gi130507685refNP_0623 ----------------------------------------------------------------------------------------------------
Mmu_VIIa_gi115511010refNP_0326 ----------------------------------------------------------------------------------------------------
Mmu_VIIb_gi261823963refNP_1157 ----------------------------------------------------------------------------------------------------
Ddi_0301_gi166240253refXP_0017 ----------------------------------------------------------------------------------------------------
Ddi_1951_gi66823681refXP_64519 ----------------------------------------------------------------------------------------------------
Sce_9711_gi6324902refNP_014971 ----------------------------------------------------------------------------------------------------
Sce_9373_gi6319290refNP_009373 ----------------------------------------------------------------------------------------------------
Mmu_MyoVc_gi124486759refNP_001 ----------------------------------------------------------------------------------------------------
Mmu_MyoVa_gi115511052refNP_034 ----------------------------------------------------------------------------------------------------
Mmu_MyoVb_gi46399202refNP_9638 ----------------------------------------------------------------------------------------------------
Ddimyo                         KRALEGELEELRETVEEAEDSKSEAEQSKRLVELELEDARRNLQKEIDAKEIAEDAKSNLQREIVEAKGRLE--EESIARTNSDRSRKRLEAEIDALTAQ
Sce_1888_gi6321812refNP_011888 ----------------------------------------------------------------------------------------------------
Sar_1270_SARC_01270T0Sphaerofo FDRSNAKARNMQAQNEELEMEVGSLRTKNRRQVTEINE--------------ANEMAEQLRSQLLSTQSRFG--RTARARARS-----------------
Mbr_4119_nullMonosigabrevicoll AKKMQSQIESLKLMVDEAEERAFRAESKMRRAERDRDE--------------LSESLSALESKMGHLQTHAK--NARLR---------------------
Sro_4749_PTSG_04749T0Salpingoe AAKLREKIKKLQAELDTAEDEVTSSMAKARRAEREAQD--------------MQESLAVLQEENTKLRSMLR--RARLRREE------------------
PpiMHCa                        MEKANSRMKTLKRQIDQNFDEMSAANAAKRKLQRELDE--------------QIESNDSLQRENNTLKQRQR--TGSVAGLPIGRKISRQSSERGFSTGG
Aqu_8281_gi340368578refXP_0033 ADKQQSRVRSLKRALEEAEEENARIQAQKRRVQREADE--------------LQEQLEAIQRDRSKSTSARR--QTVSSALGRSGRRGSNKAGPSPLNRS
Cow_3939_CAOG_03939T0Capsaspor LDKATSKLKTQKRAIDEAEEENQRNQAAKRKLKAEFDE--------------QTAELEAAQREVTSLRAKVR--GGGASAVS------------------
TaMHCa                         ADKASSRLRNMKRQLDEAEEETSRQTALKRKMQRDLDE--------------QMEVSEALTREINALKTRMR--LVNYFIAFFLLRF-------------
Asumyo                         ADRANVRARQLRRQLDEMEEEMARERTKARNLQREIDD--------------LNEANDTLARDNTNLRGQRRAPRESLLGGRRIHSIRNSSPHDDLDSIG
Hma_21673571_gi221130996refXP_ VEKANNRVKSIKRQLDESEEEVSRLNGTKRKMQRDLDE--------------NTEALESAQRELTQLKSRMK--TATTPSTRS-----------------
Nve_JGI5820_jgiNemve1118073jgi MEKTSGRMKALKRQVDEAEEESSRLNSSKRKLQRELDD--------------AQAENEELNRTVNSLRDRIR----------------------------
Mmu_Myo14_gi29336026refNP_0822 LEKSNLRLKQLKRQLEEAEEEASRAQAGRRRLQRELED--------------VTESAESMNREVTTLRNRLR--RGPLTFTTRTVRQVFRLEEGVASDEE
MmsmMHC11                      AEKGNTKVKQLKRQLEEAEEESQCINANRRKLQRELDE--------------ATESNEAMGREVNALKSKLR--RGNEASFVPSRRAGGRRVIENTDGSE
Mmu_Myo9_gi114326446refNP_0718 ADKASTRLKQLKRQLEEAEEEAQRANASRRKLQRELED--------------ATETADAMNREVSSLKNKLR--RGDLPFVVTRRIVRKG----------
MmuMHC10                       MEKANARMKQLKRQLEEAEEEATRANASRRKLQRELDD--------------ATEANEGLSREVSTLKNRLR--RGGPISFSSSRSGRRQLHIEGASLEL
Lpenm                          MDKSSARVKALKRQLDEAEEEVTRLNTQRRKLQRDLDE--------------QMEQNQIVSREMSNRRQFRA--QSRTPRTIIS----------------
LgnmMHCII1                     VEKANNRVKALKRNIDEAEEEIARLTANKRKLQRELDD--------------QVEQNETHSREITSLR-KLR--GPGGTTRTTTRTT-------------
Aaesmnm                        IEKMNNRMKILKRNLDEAEEEIQKEKTQKRKAQREYED--------------MLESHEALTREVNSLKTKLR--RGGLGVSSTRLTTPKREN--------
Dme_zipper_gi62471805refNP_001 MDKLNSRIKLLKRNLDETEEELQKEKTQKRKYQRECED--------------MIESQEAMNREINSLKTKLR--RTGGIGLSSSRLTGTPSSKRAGGGGG
PpiMHCb1                       IDFQNNKMKALRRNIDDGEAQIAVMQNKLRKAIGDVEE--------------AEERAAMLETQLVRGGSSGS--RVTKITTRRVMYSSGYPSIRASSRVP
PpiMHCb2                       IDMNNNKIKSFKRQLDEAETQIQQLNSKLRKAQNEAAE--------------AEERADMAESSLSR-ASTRN--RTAPARLGRIGY------------LP
Mmu_Myo15_gi261245016refNP_001 SDKLQLKVQSYKQQVEAAEAQANQYLSKYKKQQHELNE--------------AKERAEAAESQVNKLRAK-A--KELEKKV-------------------
Mmu_Myo7B_gi145864471refNP_001 VDKLQSKVKSYKRQFEEAEQQASTNLAKYRKAQHELDD--------------AEERADMAETQANKLRAR-S--RDALGPK-------------------
MmcMHC7                        VDKLQLKVKAYKRQAEEAEEQANTNLSKFRKVQHELDE--------------AEERADIAESQVNKLRAK-S--RDIGAKGL------------------
Mmu_Myo6_gi6754774refNP_034986 VDKLQLKVKAYKRQAEEAEEQANTNLSKFRKVQHELDE--------------AEERADIAESQVNKLRAK-S--RDIGAKKM------------------
Mmu_Myo13_gi124486959refNP_001 VDKLQAKVKSYKRQAEEAEEQANTQLSRCRRVQHELEE--------------AEERADIAESQVNKLRAK-S--RDVGGQKM------------------
Mmu_Myo3_gi153792649refNP_0010 VDKLQVKVKSYKRQAEEADEQANAHLTKFRKAQHELEE--------------AEERADIAESQVNKLRAK-T--RDFTSSRM------------------
MmskMHC8                       VDKLQAKVKSYKRQAEEAEEQSNANLAKFRKLQHELEE--------------AEERADIAESQVNKLRVK-S--REVHTKIS------------------
Mmu_Myo2_gi205830428refNP_0010 VDKLQAKVKSYKRQAEEAEEQSNTNLSKFRKIQHELEE--------------AEERADIAESQVNKLRVK-S--REVHTKII------------------
Mmu_Myo4_gi67189167refNP_03498 VDKLQTKVKAYKRQAEEAEEQSNVNLAKFRKIQHELEE--------------AEERADIAESQVNKLRVK-S--REVHTKVI------------------
Mmu_Myo1_gi82524274refNP_10960 VDKLQSKVKAYKRQAEEAEEQSNVNLAKFRKIQHELEE--------------AEERADIAESQVNKLRVK-S--REVHTKII------------------
Hma_21579261_gi221113859refXP_ ANKSEQKFKKLKVQFDESEQQVSQWQSKFKKASAELED--------------ALERCEAAEQALQKARQRNS--SAASTPGTRGPST-------------
Asumyo4                        VEKLQSKIKTQKKQLEDAEELANLNLQKYRQIQHQLED--------------AEERAENAESAVAKMRAK-S--RTTTSAAP------------------
AqMHCIIb                       VNSLNTKVRNLRRDKEDAESEVESIQKKLRQAKSQLEE--------------AEEESSSLKAQVSKLRSGAG--ATRKAKLP------------------
Aaesmnmmy                      VDKLQQKIKTYKRQIEEAEEIAALNLAKFRKAQQELEE--------------AEERADLAEQAATKFRSKGG--RGGSAQRG------------------
DmeMHCC                        VDKLQQKIKTYKRQIEEAEEIAALNLAKFRKAQQELEE--------------AEERADLAEQAISKFRAK-G--RAGSVGRG------------------
Lpemyosin                      VDKLQNKIKTYKRQVEEAEEIAAINLAKFRKVQQELED--------------AEERADQSEGALQKLRAK-N--RSSVSAAR------------------
LgMHCII3                       VEKLQDKTRTYKKQVEEAEEVASLNLAKFRKVQSELED--------------AEERAETAESYSNKLRAK-N--RSSVSQSR------------------
TaMHCb                         AEKNNHKMRVLRRQLDEAEEQTTQYQSKVRKLQHEIEE--------------CEERAEAAESALAKAKSR-A--RIALILFR------------------
Nve_SB1                        ----------------------------------------------------------------------------------------------------

                                     2410      2420      2430      2440      2450      2460      2470
                               ....|....|....|....|....|....|....|....|....|....|....|....|....|....|....|....
Mmu_MyoX_gi130507685refNP_0623 -------------------------------------------------------------------------------
Mmu_VIIa_gi115511010refNP_0326 -------------------------------------------------------------------------------
Mmu_VIIb_gi261823963refNP_1157 -------------------------------------------------------------------------------
Ddi_0301_gi166240253refXP_0017 -------------------------------------------------------------------------------
Ddi_1951_gi66823681refXP_64519 -------------------------------------------------------------------------------
Sce_9711_gi6324902refNP_014971 -------------------------------------------------------------------------------
Sce_9373_gi6319290refNP_009373 -------------------------------------------------------------------------------
Mmu_MyoVc_gi124486759refNP_001 -------------------------------------------------------------------------------
Mmu_MyoVa_gi115511052refNP_034 -------------------------------------------------------------------------------
Mmu_MyoVb_gi46399202refNP_9638 -------------------------------------------------------------------------------
Ddimyo                         VDAEQKAKNQQIKENKKIETELKEYRKKFGESEKTKTKEFLVVEKLETDYKRAKKEAADEQQQRLTVENDLRKHLSEIS
Sce_1888_gi6321812refNP_011888 -------------------------------------------------------------------------------
Sar_1270_SARC_01270T0Sphaerofo --KGLTEDDADMDVDTPSTTEITTAGEE---------------------------------------------------
Mbr_4119_nullMonosigabrevicoll -------------------------------------------------------------------------------
Sro_4749_PTSG_04749T0Salpingoe --LVGDTEQVLDTTLSESVVDDE--------------------------------------------------------
PpiMHCa                        GGNFRSKGSRERGTENDELSDEEL-------------------------------------------------------
Aqu_8281_gi340368578refXP_0033 DESPDDDEDI---------------------------------------------------------------------
Cow_3939_CAOG_03939T0Capsaspor --SRRAARDEPADDATDA-------------------------------------------------------------
TaMHCa                         --GYIISSDVHIYEI----------------------------------------------------------------
Asumyo                         TEGSDLTDDLKRGPPLKE-------------------------------------------------------------
Hma_21673571_gi221130996refXP_ --TGRRRKDDDEQNDEDGTD-----------------------------------------------------------
Nve_JGI5820_jgiNemve1118073jgi -------------------------------------------------------------------------------
Mmu_Myo14_gi29336026refNP_0822 EAEGAEPGSAPGQEPEAPPPATPQ-------------------------------------------------------
MmsmMHC11                      EEMDARDSDFNGTKASE--------------------------------------------------------------
Mmu_Myo9_gi114326446refNP_0718 --TGDCSDEEVDGKADGADAKAAE-------------------------------------------------------
MmuMHC10                       SDDDTESKTSDVNDTQPPQSE----------------------------------------------------------
Lpenm                          --TVRSSDASVEKEESDEEIEPAAEK-----------------------------------------------------
LgnmMHCII1                     -ITSKILDDSIDGDEDDKNDTAET-------------------------------------------------------
Aaesmnm                        --DSMVQDESLDGEDNSN-------------------------------------------------------------
Dme_zipper_gi62471805refNP_001 GDDSSVQDESLDGEDSAN-------------------------------------------------------------
PpiMHCb1                       SRATSPAPKAEE-------------------------------------------------------------------
PpiMHCb2                       NARTQGGEENEE-------------------------------------------------------------------
Mmu_Myo15_gi261245016refNP_001 ------REE----------------------------------------------------------------------
Mmu_Myo7B_gi145864471refNP_001 ------HKE----------------------------------------------------------------------
MmcMHC7                        ------NEE----------------------------------------------------------------------
Mmu_Myo6_gi6754774refNP_034986 ------HDEE---------------------------------------------------------------------
Mmu_Myo13_gi124486959refNP_001 -------EE----------------------------------------------------------------------
Mmu_Myo3_gi153792649refNP_0010 --VVHESEE----------------------------------------------------------------------
MmskMHC8                       ------AE-----------------------------------------------------------------------
Mmu_Myo2_gi205830428refNP_0010 ------SEE----------------------------------------------------------------------
Mmu_Myo4_gi67189167refNP_03498 ------SEE----------------------------------------------------------------------
```

```
Mmu_Myo1_gi82524274refNP_10960  ------SEE-----------------------------------------------------------------------
Hma_21579261_gi221113859refXP_  --PGSRARSRMRTPQHDD--------------------------------------------------------------
Asumyo4                         --GGLLVSQSTSAVSRSGSTRPRTTSFVDY--------------------------------------------------
AqMHCIIb                        --KDDDEDD-----------------------------------------------------------------------
Aaesmnmmy                       --GSPALSTV----------------------------------------------------------------------
DmeMHCC                         --ASPAPRATSVRPQFDGLAFPPRFDLAPENEF-----------------------------------------------
Lpemyosin                       --TSPM--------------------------------------------------------------------------
LgMHCII3                        --STSVTRTSVVRMS-----------------------------------------------------------------
TaMHCb                          --LYCYCMVLFLHPLSVTKSPIL---------------------------------------------------------
Nve_SB1                         --------------------------------------------------------------------------------
```
