# Supplementary figures and images for: Independent specialisation of myosin II paralogues in muscle vs. non-muscle functions during early animal evolution: a ctenophore perspective
Source: BMC Evol Biol. 2012 Jul 2;12:107. doi: 10.1186/1471-2148-12-107 (PMC3502136; doi:10.1186/1471-2148-12-107)

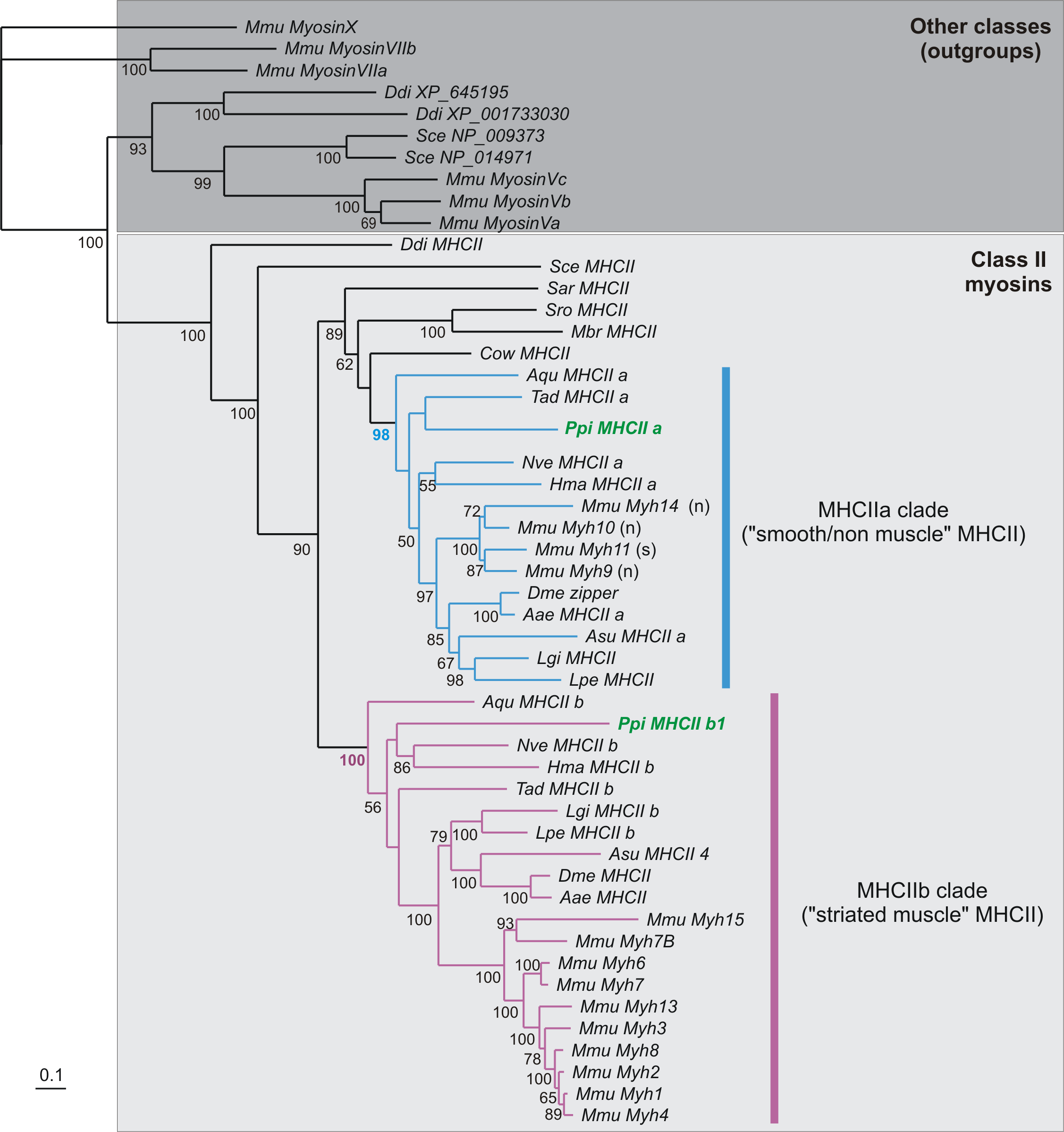

Supplement: Additional file 3 — Phylogenetic analysis rooted on non-class II myosins. Amino-acid sequences were analysed using the Maximum likelihood (ML) method. Numbers associated with the branches are ML bootstrap values (1000 replicates). Sequences from Pleurobrachia pileus are indicated in bold and green. Branches within the MHCIIa clade are in blue and within the MHCIIb clade in purple. The tree was rooted with sequences of myosin classes V, VII and X (outgroups). The letter between parentheses after the name of mouse genes of the MHCa clade indicate whether the gene is expressed in non-muscle cells (n) or in smooth muscle cells (s). The PpiMHCIIb2 gene was excluded because its partial sequence contains only the tail and therefore it has no alignable residues with non-class II myosins (only the head being homologous between class II and non-class II myosins). Abbreviations for species names: Aae: Aedes aegypti; Aqu: Amphimedon queenslandica; Asu: Ascaris suum; Cow: Capsaspora owczarzaki; Ddi: Dictyostelium discoideum; Dme: Drosophila melanogaster; Hma: Hydra magnipapillata; Lgi: Lottia gigantea; Lpe: Loligo pealei; Mbr: Monosiga brevicollis; Mmu: Mus musculus; Nve: Nematostella vectensis; Ppi: Pleurobrachia pileus; Sar: Sphaeroforma arctica; Sce: Saccharomyces cerevisiae; Sro: Salpingoeca rosetta; Tad: Trichoplax adhaerens. [file 1471-2148-12-107-S3.tiff]

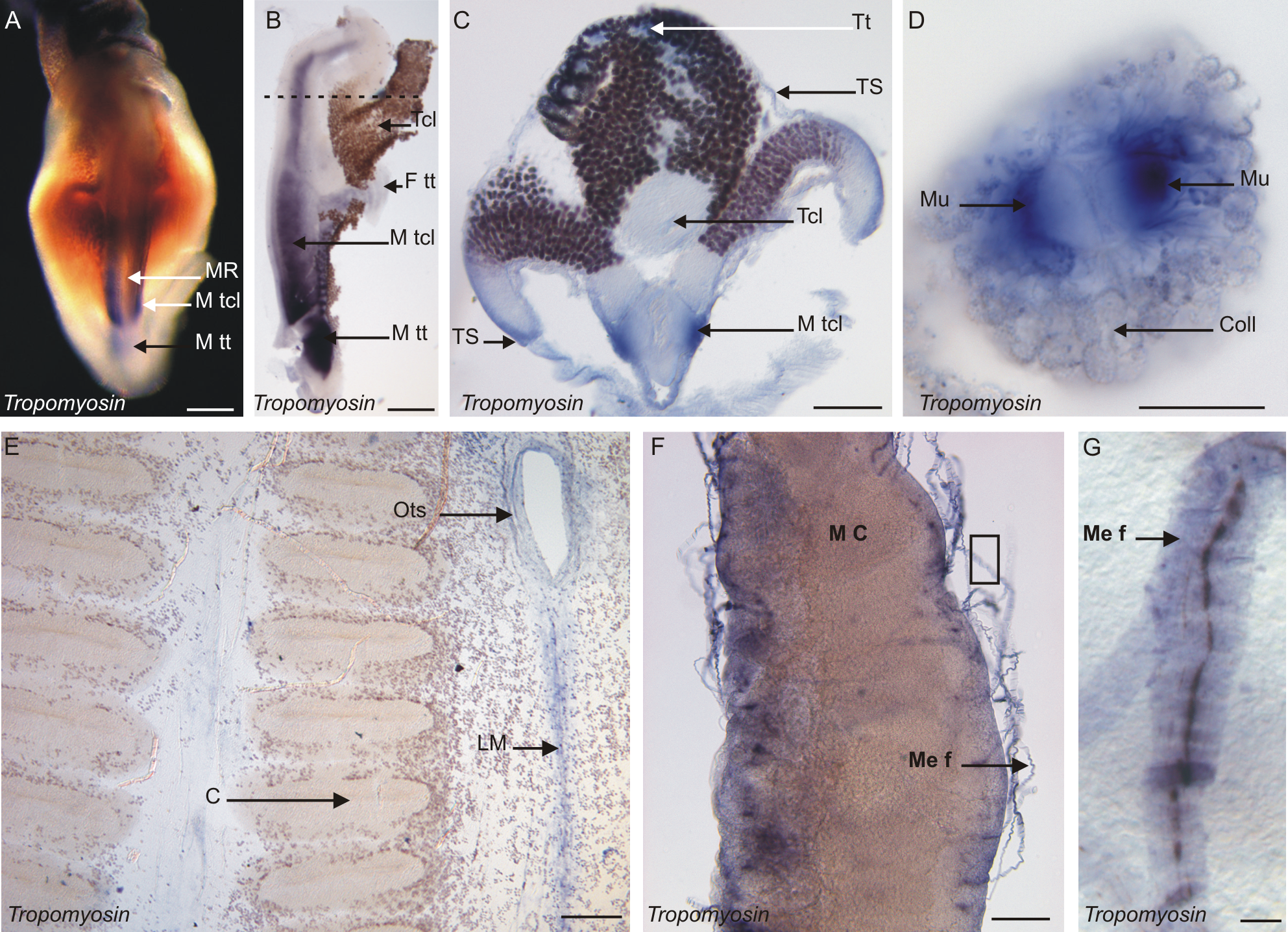

Supplement: Additional file 5 — Expression pattern of PpiTropomyosin gene in Pleurobrachia pileus muscles. (A) Internal view of a dissected tentacle root stained with the PpiTropomyosin antisense probe (CU420922.1). (B) Longitudinal view of the tentacle root (after removal of lateral expansions) showing PpiTropomyosin expression in tentacle and tentilla muscle progenitors. Forming tentillae are also stained. (C) Transverse cryosection (according to the dotted line on (B)) of a tentacle root after PpiTropomyosin whole-mount ISH. The tentacle base is also sectioned transversally (Tcl). Note that the musculature of the tentacle sheath lining is also stained (TS). (D) Transverse cryosection of a tentilla after PpiTropomyosin whole-mount ISH (compare with Figure 4V). (E) Expression of PpiTropomyosin in longitudinal muscle fibres (LM) in the tentacular plane. (F) Expression of PpiTropomyosin in mesogleal muscle fibres connected to a meridional canal. (G) Higher magnification of PpiTropomyosin expression in a mesogleal muscle fibre. C: Comb; Coll: Colloblasts; F tt: Forming tentillae; LM: Longitudinal Muscle fibres; MC: Meridional Canal; Me f: Mesogleal muscle fibre; MR: Median Ridge; M tcl: Tentacle Muscle progenitors; M tt: Tentilla Muscles progenitors; Mu: Muscle fibres; Ots: Orifice of tentacle sheath; Tcl: Tentacle; TS: Tentacular Sheath; Tt: Tentilla. Scale bars: A, B, C, E: 200 μm; F: 100 μm; D: 25 μm; G: 10 μm. [file 1471-2148-12-107-S5.tiff]

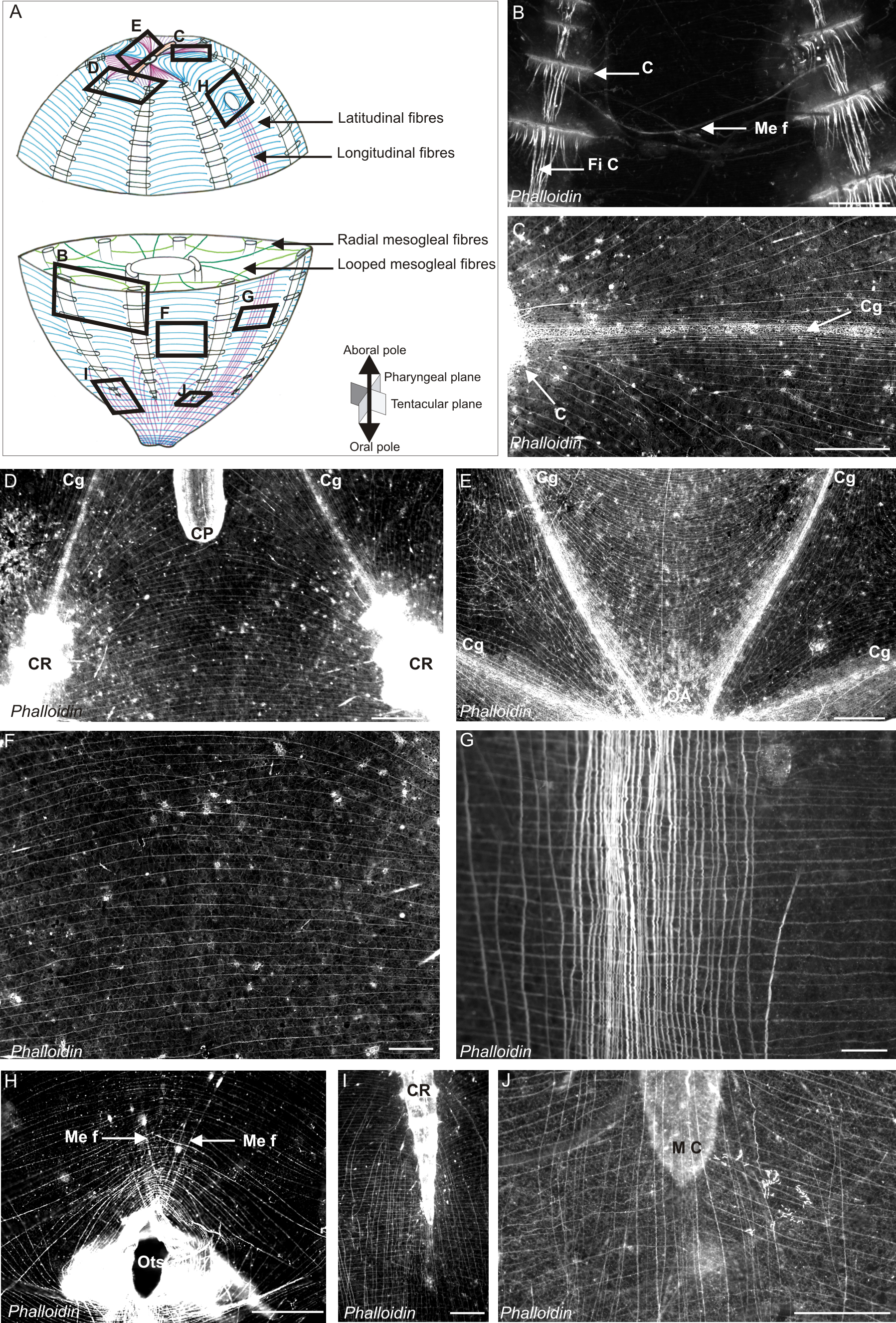

Supplement: Additional file 6 — Pleurobrachia pileus musculature (phalloidin staining). (A) Drawing of the arrangement of Pleurobrachia pileus musculature, with areas corresponding to pictures (B-I) indicated by black boxes. (B-J) Phalloidin staining of muscle fibres in selected regions of the body (boxes in (A)). (B) A large mesogleal muscle fibre connecting two meridional canals. Inter-comb fibre cells are also visible. (C) Dense parietal muscle fibres along a ciliated groove. (D) Parietal muscle fibres in the aboral region, in the pharyngeal plane. (E) Parietal muscle fibres in the aboral region, in the tentacular plane. (F) Parietal muscle fibres in the epidermis between two comb rows. All fibres have circular orientation. (G) Parietal muscles fibres in the epidermis between two comb rows in the tentacular plane: a dense band of longitudinal fibres is superimposed on the circular fibres. (H) Epithelial muscle fibres around the opening of the tentacle sheath. Note that some mesogleal fibres are visible connecting the apical organ area to the tentacle sheath. (I) Epithelial muscle fibres around the oral extremity of the comb row. (J) Higher magnification of epithelial muscles fibres at the oral extremity of the comb row. The light coloured structure visible at the top of the picture is the oral extremity of the meridional canal underlying the comb row. C: Comb; Cg: Ciliated groove; CR: Comb row; Fi C: inter-comb fibrous cells; M C: Meridional canal; Me f: Mesogleal muscle fibre; Ots: Opening of tentacle sheath. Scale bars: B-J: 100 μm. [file 1471-2148-12-107-S6.tiff]
